# Supplementary material for: Combined MEK and PARP inhibition enhances radiation response in rectal cancer
Source: Cell Rep Med. 2025 Aug 8;6(8):102284. doi: 10.1016/j.xcrm.2025.102284 (PMC12432356; doi:10.1016/j.xcrm.2025.102284)
Supplement: Document S1. Figures S1–S13 and Tables S1, S3, and S4 [file mmc1.pdf]

Cell Reports Medicine, Volume *6*

Supplemental information

Combined MEK and PARP inhibition enhances radiation response in rectal cancer

Qiyun Xiao, Julian E. Riedesser, Theresa Mulholland, Zhenchong Li, Jonas Buchloh, Philipp Albrecht, Xinchen Yang, Moying Li, Nachiyappan Venkatachalam, Olga Skabkina, Anna Klupsch, Ella Eichhorn, Li Wang, Sebastian Belle, Nadine Schulte, Daniel Schmitz, Matthias F. Froelich, Kyrhatii Trikhirhisthit, Erica Valentini, Kim E. Boonekamp, Yvonne Petersen, Thilo Miersch, Elke Burgermeister, Carsten Herskind, Marlon R. Veldwijk, Christoph Brochhausen, Robert Ihnatko, Jeroen Krijgsveld, Ina Kurth, Yuxing Zhu, Yanni Ma, Ke Cao, Michael Boutros, Matthias P. Ebert, Tianzuo Zhan, and Johannes Betge

Supplementary Figures

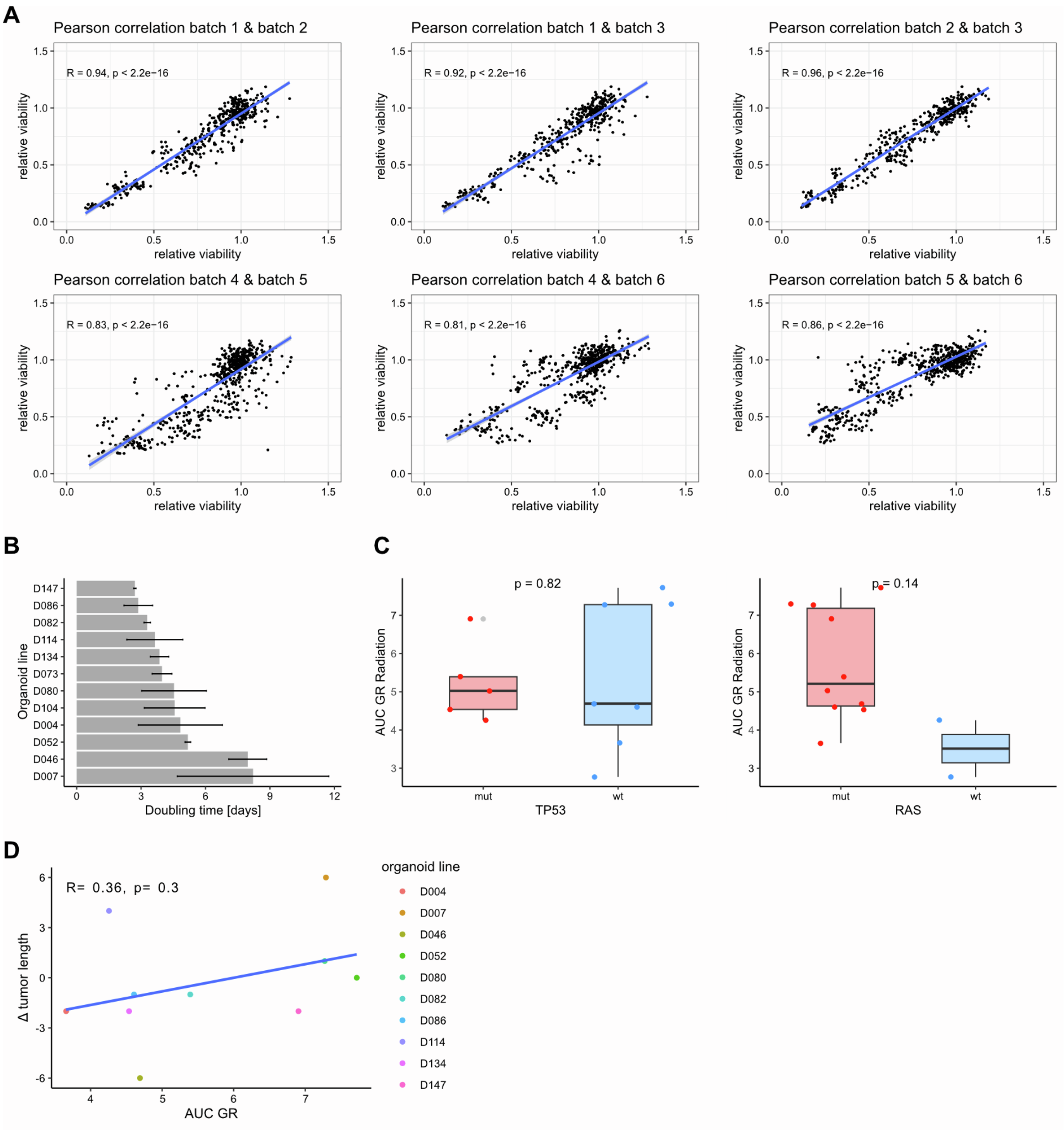

Figure S1: An organoid platform recapitulates clinical responses of rectal cancer to radiation, related to Figure 1. A, Quality controls for the organoid radiation assay, Pearson correlation of biological replicates of the radiation assay are shown in different batches. **B,** Doubling time of organoid lines used in the radiation response assay. Mean ± sd of three biological replicates are presented. **C,** Associations of the response to radiation and TP53 or RAS mutation status, two-tailed t-test. **D,** Pearson correlation of organoid response to radiation (AUC GR) and Δ tumor length before and after radiation therapy, measured in MRI images.

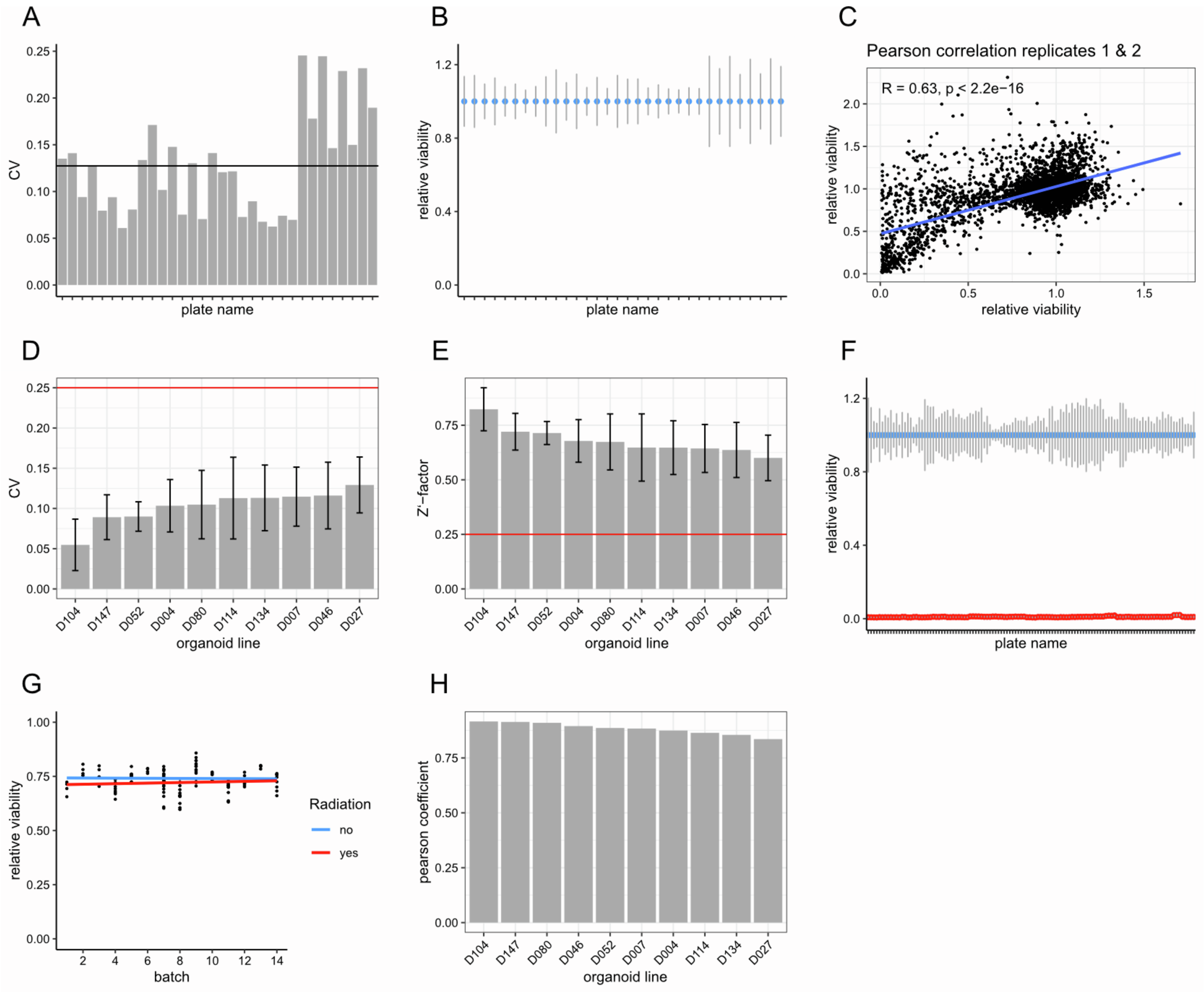

Figure S2: Quality control of drug-radiation screening experiments, related to Figure 2. A-H, Quality controls for the drug-radiation synergism screens. A kinase library of 224 drugs in 4 concentrations was tested in 2 organoid lines. 10 organoid lines were screened with a clinical drug library containing 140 compounds in 5 concentrations. DMSO was used as negative control while high-concentrated bortezomib was the positive control in the clinical library. For each line 2-4 replicates were analyzed. **A,** CV values of the DMSO controls in the kinase library combination screen are plotted for each plate. All CV values were < 2.5. **B,** Normalized luminescence values of DMSO controls for each plate are plotted as mean ± standard deviation. **C,** Pearson correlation of replicates 1 and 2 in the kinase library combination screen. **D,** Mean CV values of the DMSO controls in the clinical library combination screen are plotted as mean ± standard deviation of 2-4 biological replicates. All mean CV values were < 0.25. **E,** Z'-factor was calculated of the raw luminescence values for each plate using the formula described in the Methods section. Mean Z`-factors ± standard deviation of 2-4 biological replicates are shown. F, Distribution of normalized luminescence values of positive and negative controls in the clinical library combination screen. **G,** Mean viability for each plate for different batches. No tendencies were detected. **H,** Average Pearson correlation coefficient of normalized values of 2-4 biological replicates is plotted for each line.

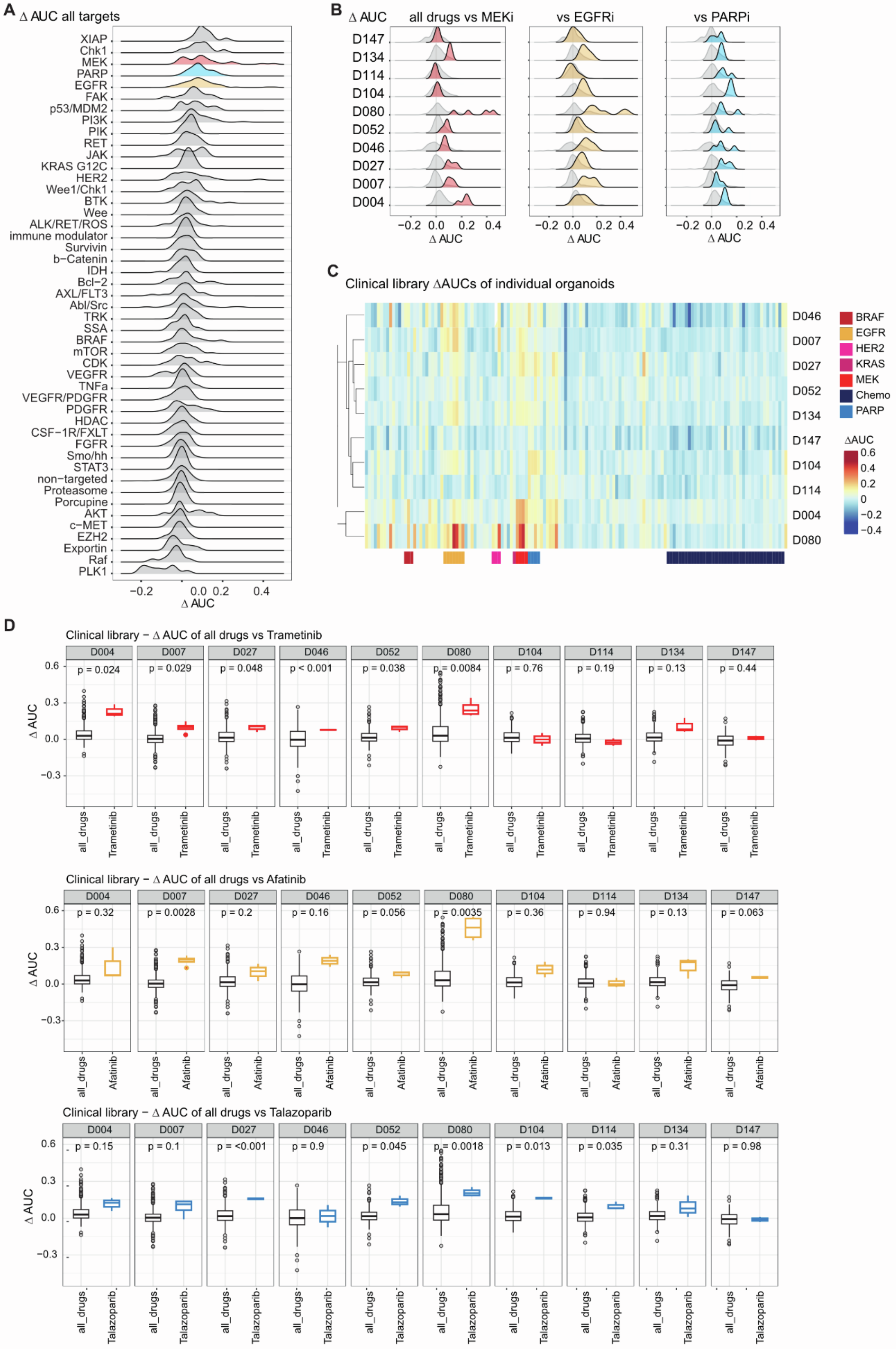
A
Δ AUC all targets
XIAP
Chk1
MEK
PARP
EGFR
FAK
p53/MDM2
PI3K
PIK
RET
JAK
KRAS G12C
HER2
Wee1/Chk1
BTK
Wee
ALK/RET/ROS
immune modulator
Survivin
b−Catenin
IDH
Bcl−2
AXL/FLT3
Abl/Src
TRK
SSA
BRAF
mTOR
CDK
VEGFR
TNFa
VEGFR/PDGFR
PDGFR
HDAC
CSF−1R/FXLT
FGFR
Smo/hh
STAT3
non−targeted
Proteasome
Porcupine
AKT
c−MET
EZH2
Exportin
Raf
PLK1
−0.2
0.0 0.2
0.4
Δ AUC
B
Δ AUC
all drugs vs MEKi
vs EGFRi
vs PARPi
D147
D134
D114
D104
D080
D052
D046
D027
D007
D004
−0.2 0.0 0.2 0.4
Δ AUC
C
Clinical library ΔAUCs of individual organoids
D046
D007
D027
D052
D134
D147
D104
D114
D004
D080
BRAF
EGFR
HER2
KRAS
MEK
Chemo
PARP
ΔAUC
0.6
0.4
0.2
0
−0.2
−0.4
D
Clinical library − Δ AUC of all drugs vs Trametinib
D004
D007
D027
D046
D052
D080
D104
D114
D134
D147
p = 0.024
p = 0.029
p = 0.048
p < 0.001
p = 0.038
p = 0.0084
p = 0.76
p = 0.19
p = 0.13
p = 0.44
Δ AUC
0.6
0.3
0.0
−0.3
all_drugs
Trametinib
Clinical library − Δ AUC of all drugs vs Afatinib
p = 0.32
p = 0.0028
p = 0.2
p = 0.16
p = 0.056
p = 0.0035
p = 0.36
p = 0.94
p = 0.13
p = 0.063
all_drugs
Afatinib
Clinical library − Δ AUC of all drugs vs Talazoparib
p = 0.15
p = 0.1
p = <0.001
p = 0.9
p = 0.045
p = 0.0018
p = 0.013
p = 0.035
p = 0.31
p = 0.98
all_drugs
Talazoparib

Figure S3: Screening for synergistic effects between drugs and radiation, related to Figure 2. A, Distribution of ΔAUCs of inhibitors stratified by mechanism of action of tested drugs in the clinical library. **B,** Distribution of ΔAUCs of MEKi, EGFRi and PARPi in individual organoid lines. **C,** Heatmap of ΔAUCs from the clinical library screen with ten rectal cancer organoids. **D,** Comparisons of ΔAUC of trametinib (MEK inhibitor) with ΔAUCs of all drugs in ten tested organoid lines (top), comparisons of ΔAUC of afatinib (EGFR inhibitor) with ΔAUCs of all drugs in ten tested organoid lines (middle) and comparisons of ΔAUC of talazoparib (PARP inhibitor) with ΔAUCs of all drugs in ten tested organoid lines (top). T-tests were done to assess statistical significance.

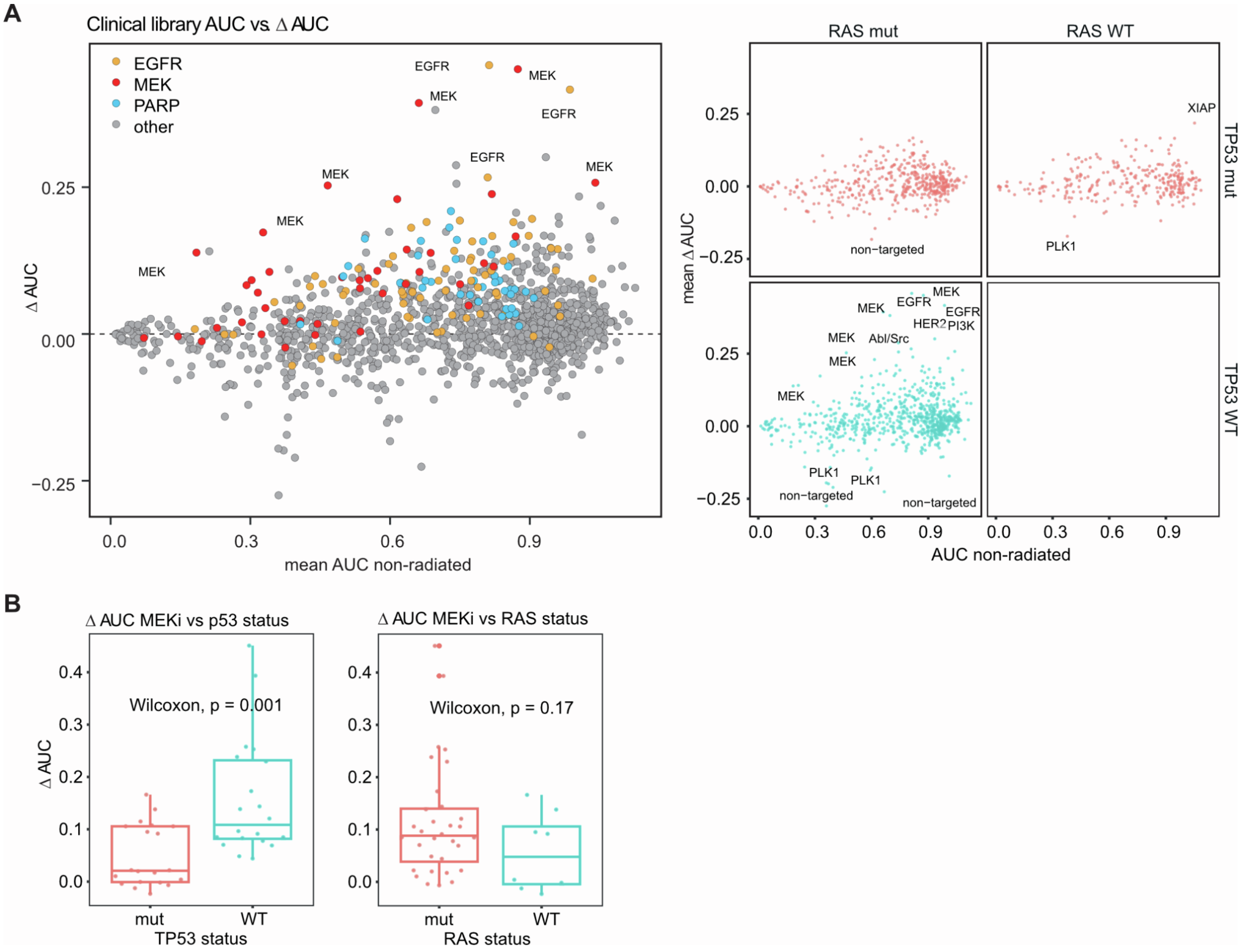

Figure S4: Influence of mutations on drug-radiation-combinations, related to Figure 2. A, Mean ΔAUCs vs. non-radiated AUCs of all lines and drugs tested in the clinical library screen with ten organoid lines according to TP53 and RAS status. **B,** Association of RAS and TP53 mutation status with MEKi ΔAUCs.

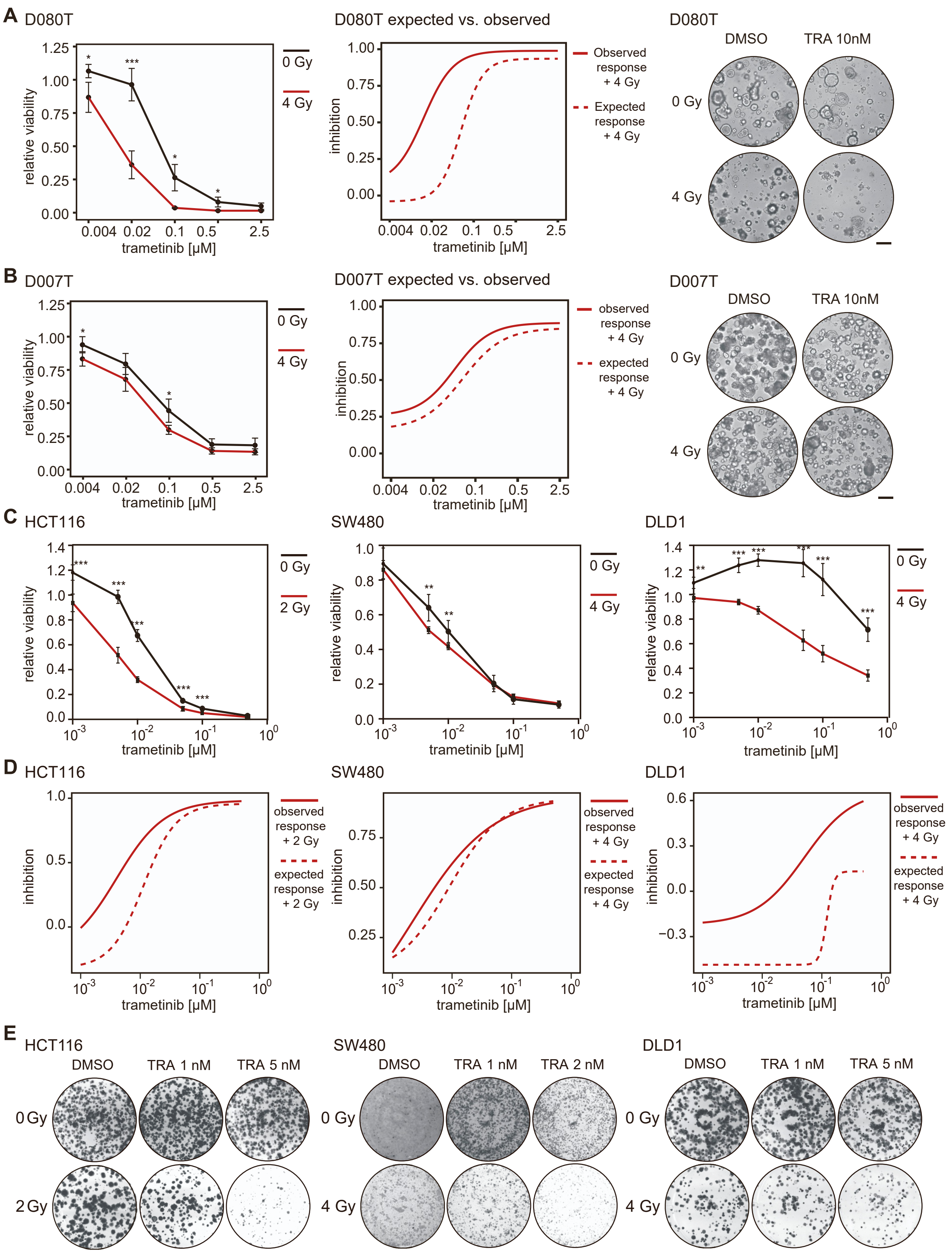

A
D080T
relative viability
trametinib [µM]
0 Gy
4 Gy
D080T expected vs. observed
inhibition
Observed response + 4 Gy
Expected response + 4 Gy
DMSO
TRA 10nM
B
D007T
D007T expected vs. observed
observed response + 4 Gy
expected response + 4 Gy
C
HCT116
SW480
DLD1
2 Gy
D
observed response + 2 Gy
expected response + 2 Gy
E
TRA 1 nM
TRA 5 nM
TRA 2 nM

Figure S5: MEK inhibition is synergistic with radiation in colorectal cancer cell lines and organoids, related to Figure 2. A, Left: viability assay of organoid line D080T treated with increasing concentrations of MEK inhibitor trametinib with and without radiation, data of the irradiated and non-irradiated plates were normalized to DMSO controls on the same plates in this analysis, respectively, to visualize the additional effect of trametinib in the irradiated condition. Middle: Dose-inhibition relationship of the same data. A Bliss' independence model of trametinib and radiation was used for each concentration to calculate the expected inhibition. For this analysis, all treatments were normalized to non-irradiated DMSO controls. Right: example images of organoids, scale bar: 50 µm. **B,** Left: viability assay of organoid line D007T treated with increasing concentrations of MEK inhibitor trametinib with- and without radiation, data of the irradiated and non-irradiated plates were normalized to DMSO controls on the same plates in this analysis, respectively, to visualize the additional effect of trametinib in irradiated condition. Middle: Dose-inhibition relationship of the same data. A Bliss' independence model of trametinib and radiation was used for each concentration to calculate the expected inhibition. For this analysis, all treatments were normalized to non-irradiated DMSO controls. Right: example images of organoids, scale bar: 50 µm. **C,** Viability assays of CRC cell lines treated with increasing concentrations of MEK inhibitor trametinib with and without radiation. Cell viability was determined after 5-6 days of treatment by CellTiter-Glo. Data of the irradiated and non-irradiated plates were normalized to DMSO controls on the same plates in this analysis, respectively, to visualize the additional effect of trametinib in irradiated condition. **D,** Dose-inhibition relationship of cell lines treated with increasing concentrations of trametinib and radiation. A Bliss' independence model of trametinib and radiation was used for each concentration to calculate the expected inhibition. For this analysis, all treatments were normalized to non-irradiated DMSO controls. **E,** Colony forming assay of CRC cell lines treated with trametinib (TRA) with and without radiation for 10-12 days. Scans of complete wells of standard six-well plates (9.6 cm^2 per well) are shown. A-B, E, representative images of at least three independent biological replicates are shown. A-D, Data from three (cell lines) and four (organoids) biological replicates are presented as mean ± SD. $^*p < 0.05$, $^{**}p < 0.01$, $^{***}p < 0.001$, two-tailed t-test.

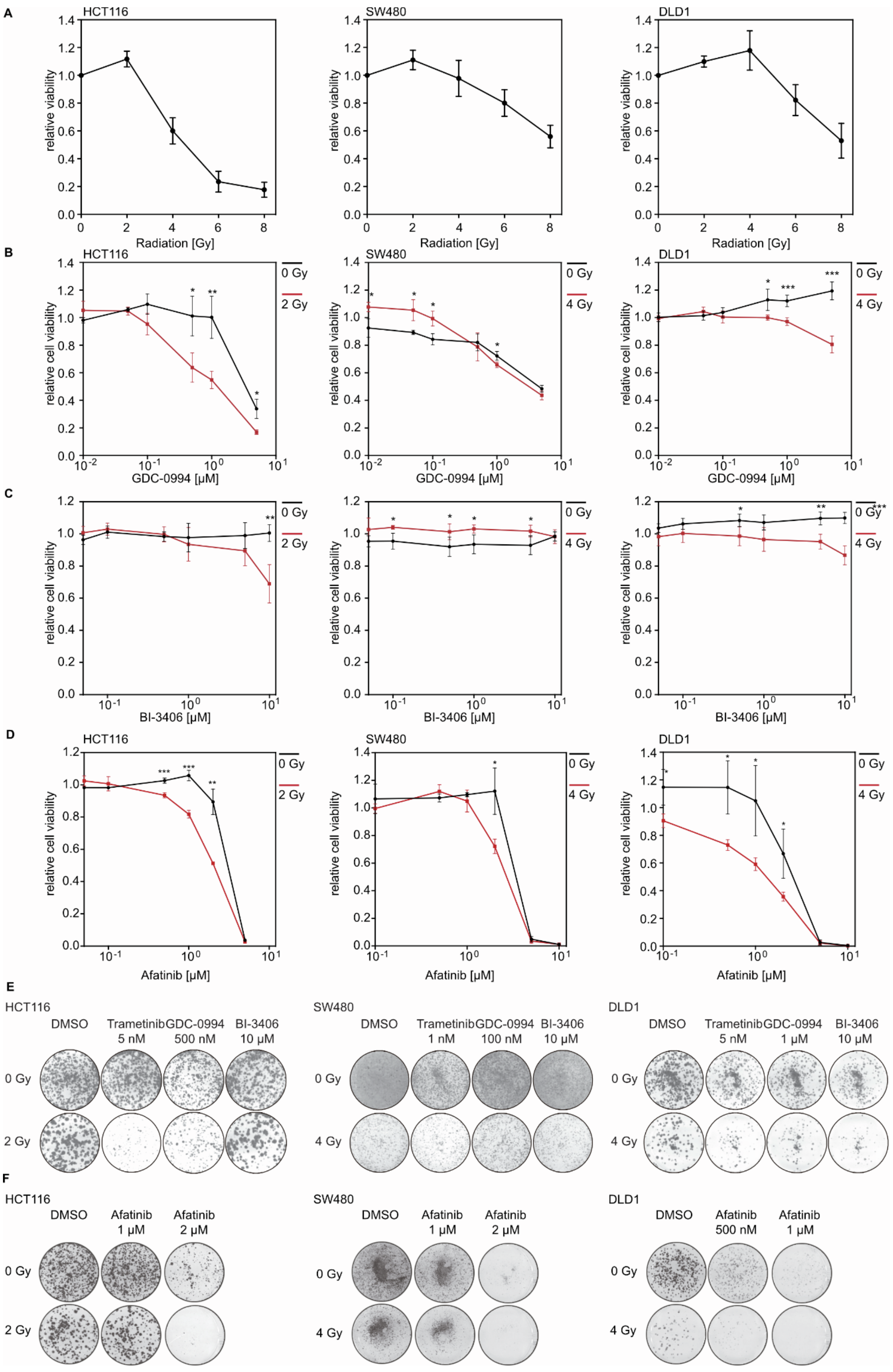
A
HCT116
SW480
DLD1
relative viability
Radiation [Gy]
B
relative cell viability
GDC-0994 [µM]
0 Gy
2 Gy
4 Gy
C
BI-3406 [µM]
D
Afatinib [µM]
E
DMSO
Trametinib 5 nM
GDC-0994 500 nM
BI-3406 10 µM
Trametinib 1 nM
GDC-0994 100 nM
GDC-0994 1 µM
F
Afatinib 1 µM
Afatinib 2 µM
Afatinib 500 nM

Figure S6: Effect of KRAS:SOS1, ERK1/2 and EGFR inhibitors on radiosensitivity of CRC cell lines, related to Figure 2. A, Intrinsic radiosensitivity of CRC cell lines. **B-D,** Viability assay of CRC cell lines treated with increasing concentrations of ERK inhibitor GDC-0994 (B), KRAS:SOS1 inhibitor BI-3406 (C) and EGFR inhibitor afatinib (D) with and without radiation. Cell viability was determined after 5-6 days treatment by CellTiter-Glo. **E,** Colony forming assay of CRC cell lines treated with trametinib, GDC-0994 or BI-3406 -/+ radiation. Scans of complete wells of standard six-well plates are shown (9.6 cm^2 per well). **F,** Colony forming assay of CRC cell lines treated with afatinib -/+ radiation. Scans of complete wells of standard six-well plates are shown (9.6 cm^2 per well).

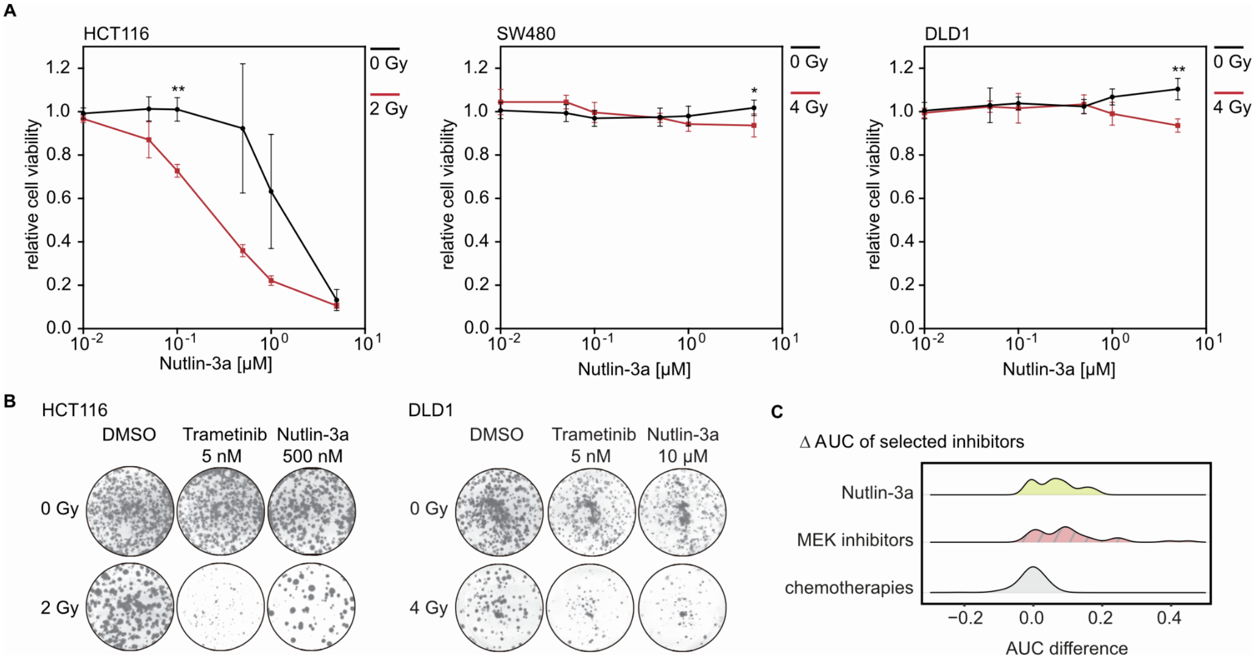

Figure S7: Effect of MDM2 inhibitor Nutlin-3a on radiosensitivity of CRC cell lines, related to Figure 2. A, Viability assay of CRC cell lines treated with increasing concentrations of MDM2 inhibitor Nutlin-3a with and without radiation. Cell viability was determined after 5-6 days of treatment by CellTiter-Glo. **B,** Colony forming assay of CRC cell lines treated with Nutlin-3a or trametinib -/+ radiation. Scans of complete wells of standard six-well plates are shown (9.6 cm^2 per well) **C,** Comparison of distribution of ΔAUC between Nutlin-3a, MEK inhibitors and chemotherapy drugs in organoid drug-radiation screen with the clinical library (Fig 2).

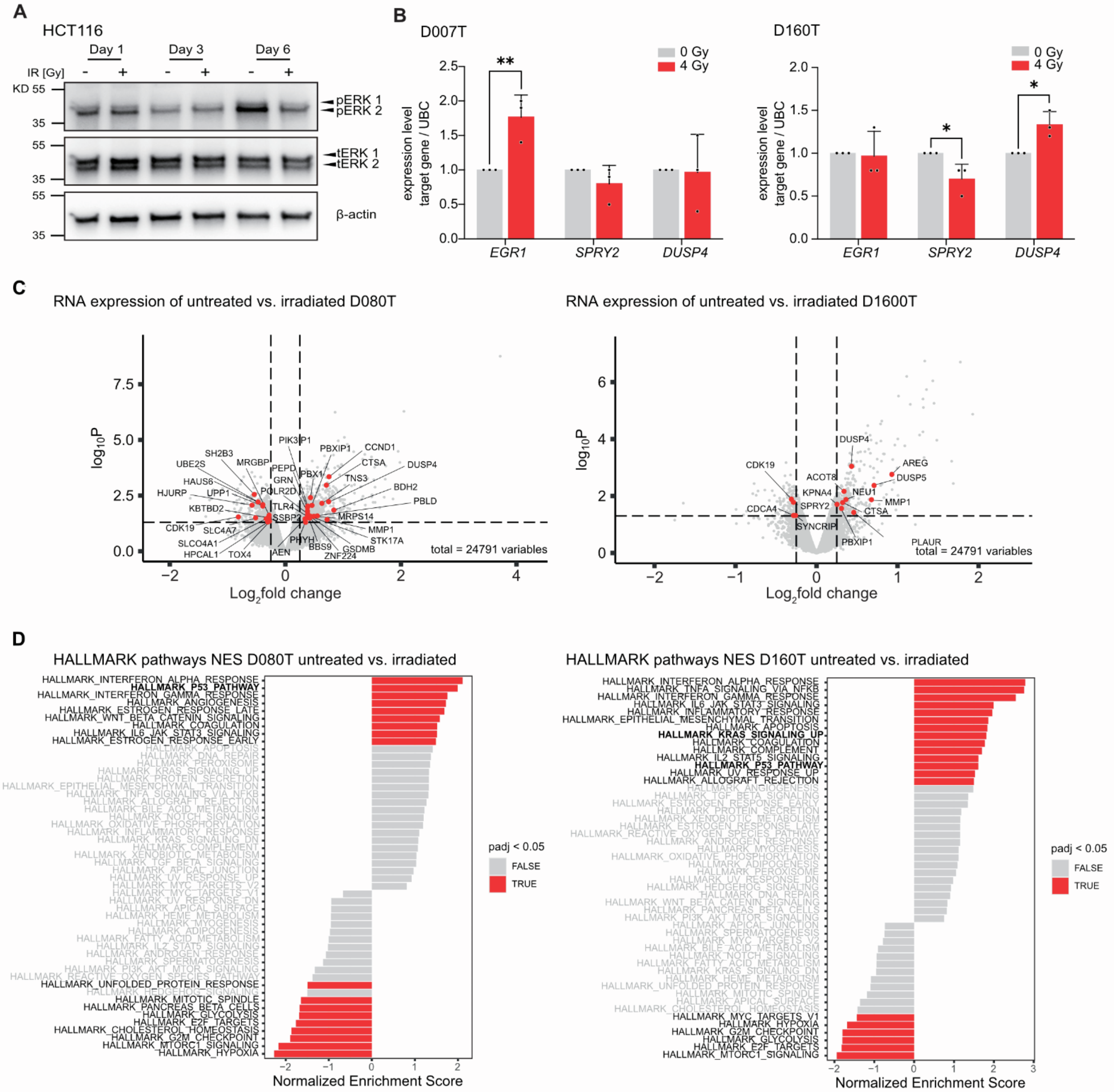
A
HCT116
Day 1
Day 3
Day 6
IR [Gy]
KD 55
35
55
35
55
35
pERK 1
pERK 2
tERK 1
tERK 2
β-actin
B
D007T
D160T
0 Gy
4 Gy
expression level target gene / UBC
EGR1
SPRY2
DUSP4
C
RNA expression of untreated vs. irradiated D080T
RNA expression of untreated vs. irradiated D1600T
log10P
Log2fold change
total = 24791 variables
D
HALLMARK pathways NES D080T untreated vs. irradiated
HALLMARK pathways NES D160T untreated vs. irradiated
HALLMARK_INTERFERON_ALPHA_RESPONSE
HALLMARK_P53_PATHWAY
HALLMARK_INTERFERON_GAMMA_RESPONSE
HALLMARK_ANGIOGENESIS
HALLMARK_ESTROGEN_RESPONSE_LATE
HALLMARK_WNT_BETA_CATENIN_SIGNALING
HALLMARK_COAGULATION
HALLMARK_IL6_JAK_STAT3_SIGNALING
HALLMARK_ESTROGEN_RESPONSE_EARLY
HALLMARK_UNFOLDED_PROTEIN_RESPONSE
HALLMARK_MITOTIC_SPINDLE
HALLMARK_PANCREAS_BETA_CELLS
HALLMARK_GLYCOLYSIS
HALLMARK_E2F_TARGETS
HALLMARK_CHOLESTEROL_HOMEOSTASIS
HALLMARK_G2M_CHECKPOINT
HALLMARK_MTORC1_SIGNALING
HALLMARK_HYPOXIA
HALLMARK_TNFA_SIGNALING_VIA_NFKB
HALLMARK_IL6_JAK_STAT3_SIGNALING
HALLMARK_INFLAMMATORY_RESPONSE
HALLMARK_EPITHELIAL_MESENCHYMAL_TRANSITION
HALLMARK_APOPTOSIS
HALLMARK_KRAS_SIGNALING_UP
HALLMARK_COMPLEMENT
HALLMARK_IL2_STAT5_SIGNALING
HALLMARK_UV_RESPONSE_UP
HALLMARK_ALLOGRAFT_REJECTION
HALLMARK_MYC_TARGETS_V1
padj < 0.05
FALSE
TRUE
Normalized Enrichment Score

Figure S8: Radiation-induced effects on signaling pathways, related to Figure 3. A, Phosphorylation of ERK1/2 in CRC line HCT116 at different time points after irradiation [IR]. **B,** Target gene expression of RAS-MAPK pathway after radiation in CRC organoid lines D007T and D160T determined by quantitative PCR. Data from three independent experiments are presented as mean ± SD. *$p < 0.05$, two-tailed Student's t-test. **C,** RNA expression profiling of rectal cancer organoid lines D080T and D160T 96 h after radiation treatment with 4 Gy. Volcano plot of differentially expressed genes in irradiated vs. non-irradiated organoids. Target genes of the EGFR signaling pathway according to PROGENY are highlighted. D, Gene set enrichment analysis of HALLMARK gene sets in irradiated vs. non-irradiated organoids D080T and D160T. C-D, data from five (D080T) and four (D160T) biological replicates are shown.

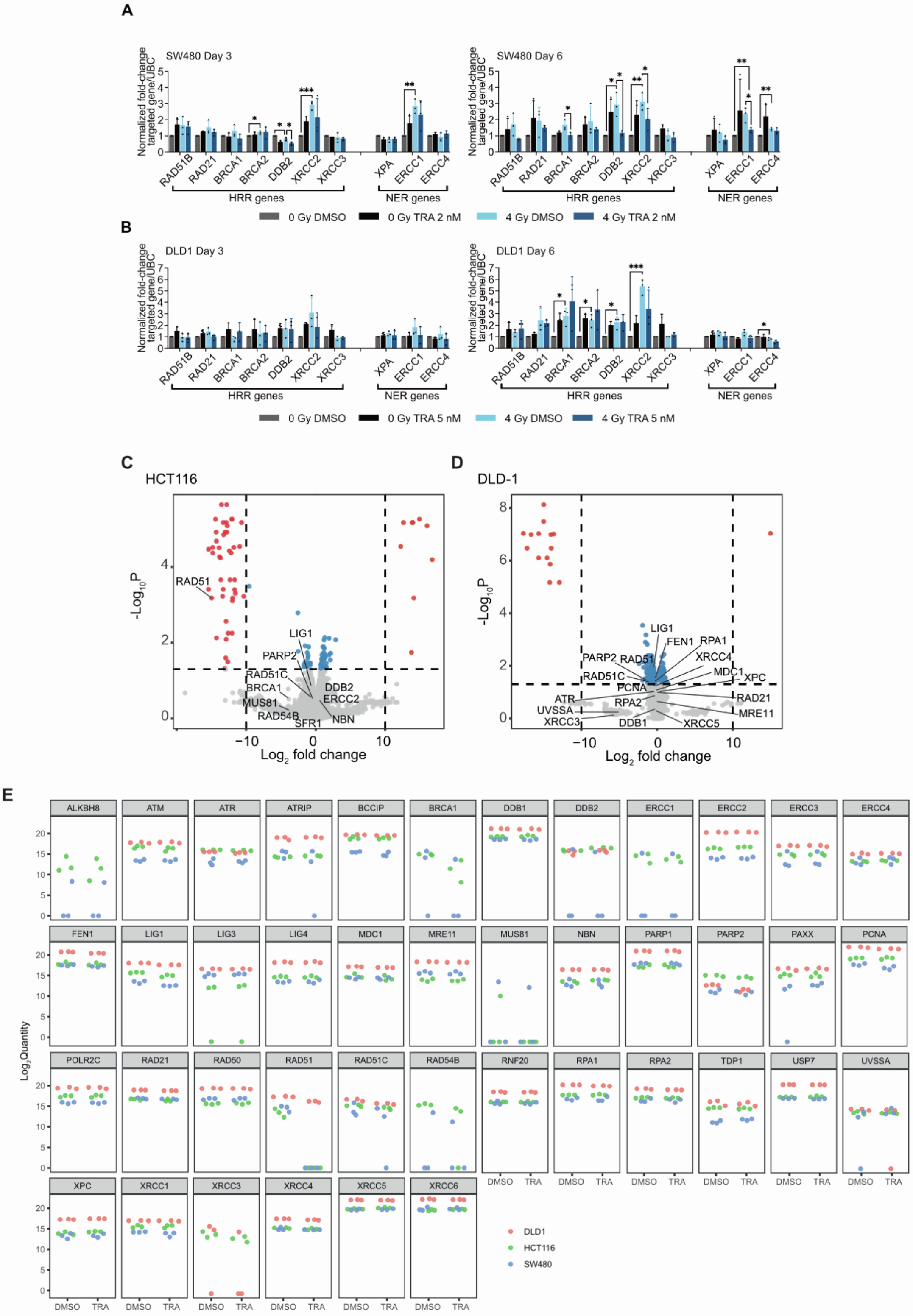
A
SW480 Day 3
SW480 Day 6
Normalized fold-change targeted gene/UBC
RAD51B
RAD21
BRCA1
BRCA2
DDB2
XRCC2
XRCC3
XPA
ERCC1
ERCC4
HRR genes
NER genes
0 Gy DMSO
0 Gy TRA 2 nM
4 Gy DMSO
4 Gy TRA 2 nM
B
DLD1 Day 3
DLD1 Day 6
0 Gy TRA 5 nM
4 Gy TRA 5 nM
C
HCT116
D
DLD-1
-Log10P
Log2 fold change
RAD51
LIG1
PARP2
RAD51C
BRCA1
MUS81
RAD54B
SFR1
NBN
DDB2
ERCC2
FEN1
RPA1
XRCC4
MDC1
XPC
RAD21
MRE11
XRCC5
DDB1
XRCC3
UVSSA
ATR
RPA2
PCNA
E
Log2Quantity
ALKBH8
ATM
ATRIP
BCCIP
ERCC3
LIG3
LIG4
PARP1
PAXX
POLR2C
RAD50
RAD51C
RAD54B
RNF20
TDP1
USP7
XRCC1
XRCC6
DMSO
TRA
DLD1
HCT116
SW480

Figure S9: Proteomics analysis of radiation-induced effects on DNA repair pathways in CRC, related to Figure 4. A-B, Radiation-induced transcriptional changes of homologous recombination repair (HRR) genes and nucleotide excision repair (NER) genes in CRC cell lines. Expression of genes is determined by qPCR. Data from three independent experiments are presented as mean ± SD. $*p < 0.05$, $**p < 0.01$, $***p < 0.001$, two-tailed Student's t-test. **C,** Global proteome profiling by mass spectrometry of HCT116 cells after treatment with 100 nM trametinib vs. DMSO for 24 h. **D,** Global proteome profiling by mass spectrometry of DLD1 cells after treatment with 100 nM trametinib vs. DMSO for 24 h. **E,** Abundance of selected DNA damage response pathway proteins in the three tested cell lines in the proteomics profiling experiments. **C-D,** three biological replicates were analyzed for each cell line.

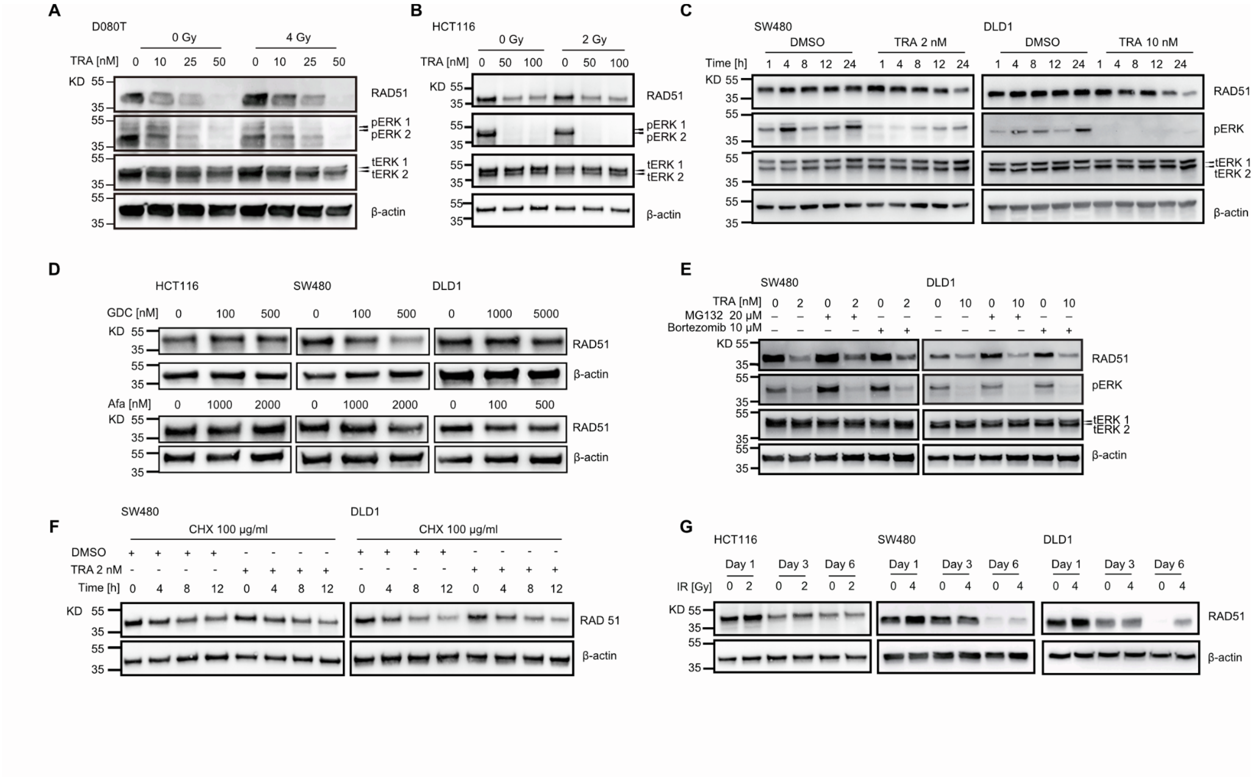

Figure S10: Radiation and MEK inhibition-induced effects on DNA repair pathway in CRC, related to Figure 4. A, Dose-dependent loss of RAD51 upon MEK inhibition with trametinib (TRA) after 48 h in the organoid line D080T. B, Dose-dependent loss of RAD51 upon MEK inhibition +/- radiation after 24 h in the CRC cell line HCT116. **C,** Temporal kinetics of RAD51 loss in SW480 and DLD1 cell lines after MEK inhibition. **D,** Differential effect of RAS-MAPK inhibitors (ERK1/2 inhibitor GDC-0994 [GDC] and EGFR inhibitor Afatinib [Afa]) on RAD51 levels in HCT116, SW480 and DLD1 cells. Cells were treated for 24 hours with the inhibitors. **E, I**nhibition of the proteasomal inhibitors bortezomib and MG132 does not prevent RAD51 loss upon MEK inhibition in the CRC cell lines SW480 and DLD1. CRC cell lines were pre-cultured for 24 h, and treated with trametinib or DMSO for 20 h, and then co-treated with proteasomal inhibitor (MG132 or bortezomib) or DMSO for 4 h. **F,** Cycloheximide chase assays with CRC cell lines. CRC cell lines were pre-incubated with trametinib for 8 h, following treatment with or without cycloheximide (CHX). No enhanced protein loss is observed upon treatment with the inhibitors. **G,** Radiation (IR) increases RAD51 protein levels in CRC cell lines at different time points. A-G, Representative images of three independent biological replicates are shown.

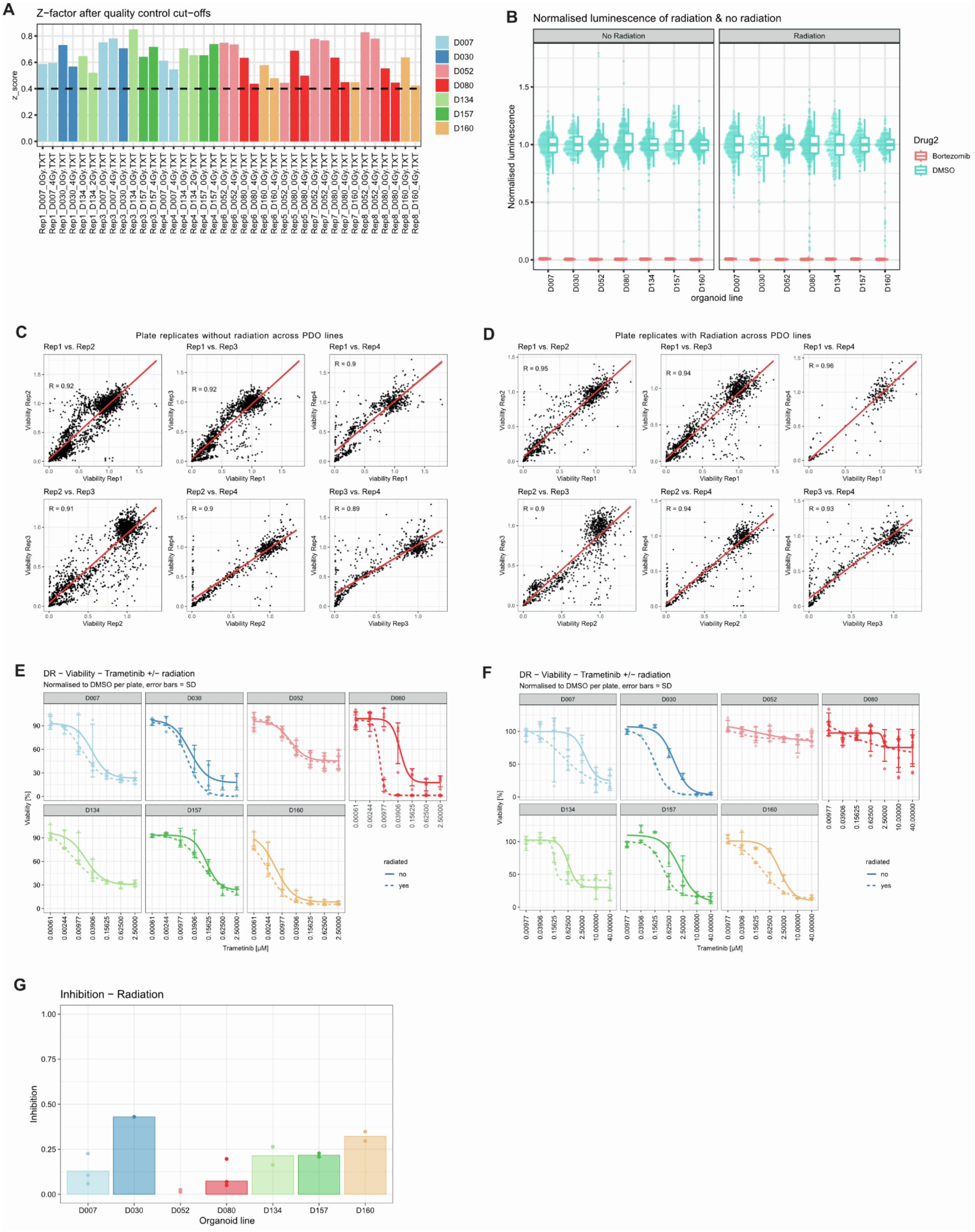

A
Z−factor after quality control cut−offs
z_score
D007
D030
D052
D080
D134
D157
D160
B
Normalised luminescence of radiation & no radiation
No Radiation
Radiation
Normalised luminescence
organoid line
Drug2
Bortezomib
DMSO
C
Plate replicates without radiation across PDO lines
Rep1 vs. Rep2
Rep1 vs. Rep3
Rep1 vs. Rep4
Rep2 vs. Rep3
Rep2 vs. Rep4
Rep3 vs. Rep4
R = 0.92
R = 0.92
R = 0.9
R = 0.91
R = 0.9
R = 0.89
D
Plate replicates with Radiation across PDO lines
Rep1 vs. Rep2
Rep1 vs. Rep3
Rep1 vs. Rep4
Rep2 vs. Rep3
Rep2 vs. Rep4
Rep3 vs. Rep4
R = 0.95
R = 0.94
R = 0.96
R = 0.9
R = 0.94
R = 0.93
E
DR − Viability − Trametinib +/− radiation
Normalised to DMSO per plate, error bars = SD
Viability [%]
Trametinib [µM]
radiated
no
yes
F
DR − Viability − Trametinib +/− radiation
Normalised to DMSO per plate, error bars = SD
Viability [%]
Trametinib [µM]
radiated
no
yes
G
Inhibition − Radiation
Inhibition
Organoid line

Figure S11: Quality controls of drug-drug combination and drug-drug-radiation combination tests, related to Figures 5-6. A-D, Quality controls of drug-drug-radiation profiling experiments. **A,** z-score and of all tested plates after quality control cut-offs were applied: four plates (D030T Rep. 3, 4 Gy, D134T Rep. 3, 4 Gy, D052T Rep. 1, 4 Gy and D160T Rep. 2, 4 Gy) were excluded from further analysis. **B,** Normalized luminescence values of positive (bortezomib) and negative (DMSO) controls in radiation and non-radiation assays. **C-D,** Plate replicate correlations of drug-drug combination tests (C) and drug-drug-radiation tests (D). **E-G,** Single(/dual) response of tested organoid lines in drug-drug and drug-drug-radiation experiments. E, Response of all tested organoid lines to trametinib alone (with/without irradiation). **F,** Response of all tested organoid lines to talazoparib alone (with/without irradiation). **G,** Growth inhibition of all tested organoids after irradiation treatment alone. 2 biological replicates were analyzed for D030T, D157T, 3 replicates were analyzed for D007T, D052T, D134T and D160, and 4 replicates were analyzed for D080T.

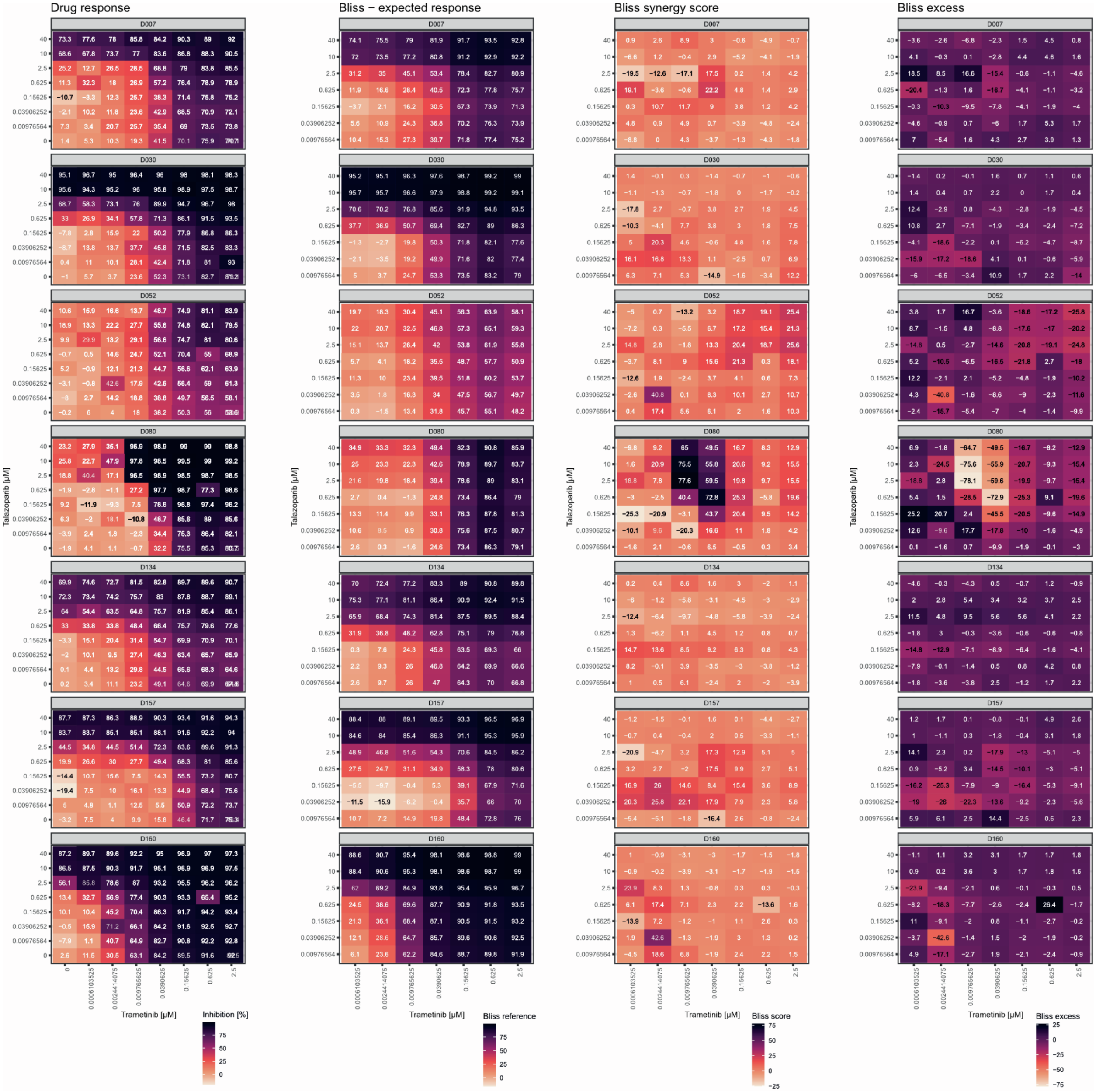
Drug response
Bliss − expected response
Bliss synergy score
Bliss excess
D007
D030
D052
D080
D134
D157
D160
Talazoparib [µM]
Trametinib [µM]
Inhibition [%]
Bliss reference
Bliss score
Bliss excess

Figure S12: PARP inhibitors synergize with MEK inhibitors in CRC models, related to Figure 5. Drug response, Bliss expected response, Bliss synergy score and Bliss excess calculated for all dose combinations of talazoparib and trametinib in all seven tested organoid lines. All data shown in this figure were obtained in absence of radiation treatment. 2 biological replicates were analyzed for D030T, D157T, 3 replicates were analyzed for D007T, D052T, D134T and D160, and 4 replicates were analyzed for D080T. Of note, due to a pipetting error in one of the master plates, six individual wells had to be excluded from analysis of organoid lines D052T, D080T and D160T, including 3 wells containing DMSO controls and 3 wells containing drug combinations. Missing values in matrices were imputed by bootstrapping within the synergyfinder package.

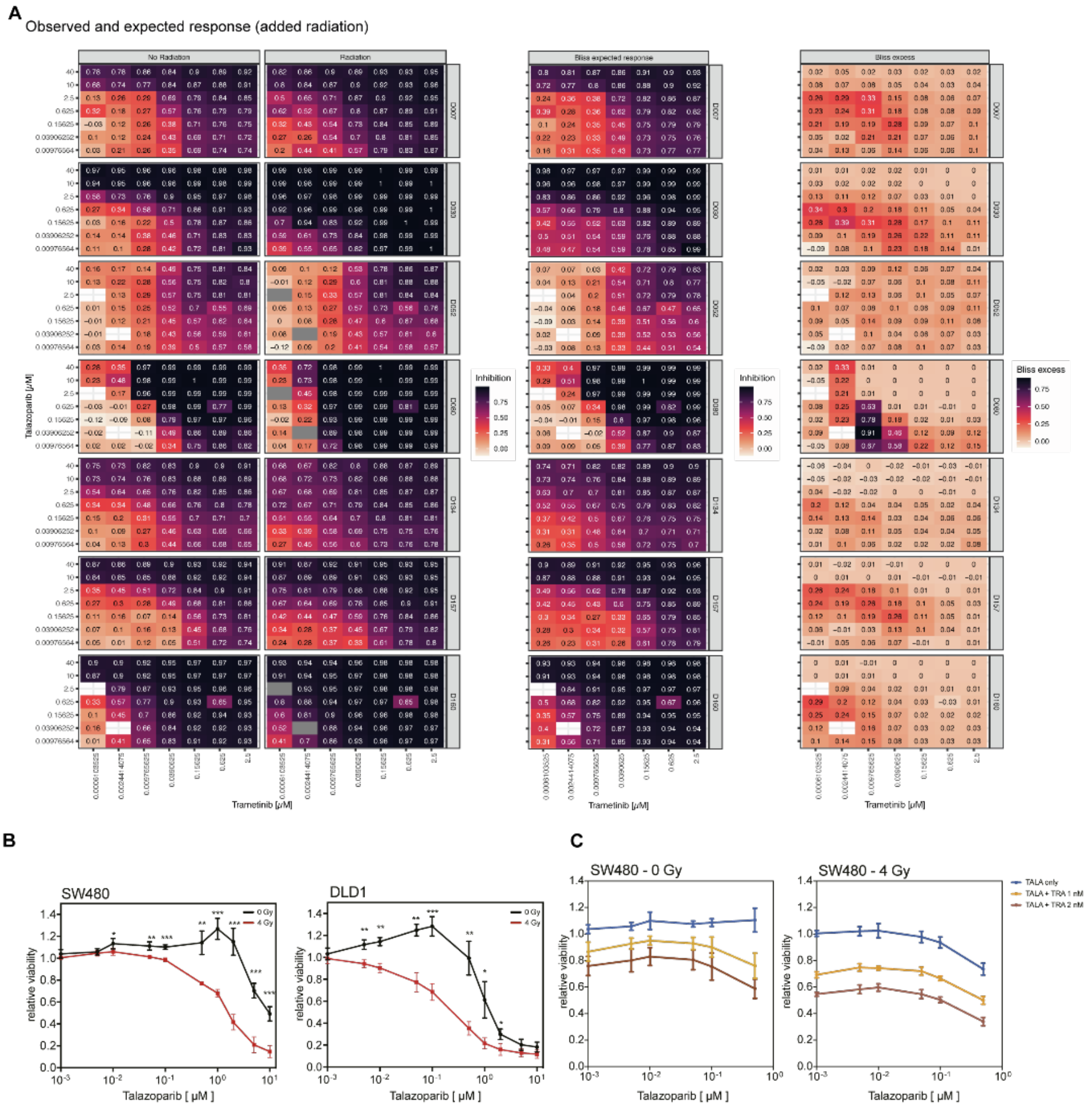
A
Observed and expected response (added radiation)
No Radiation
Radiation
Bliss expected response
Bliss excess
Talazoparib [μM]
Trametinib [μM]
Inhibition
Bliss excess
D007
D030
D052
D080
D134
D157
D160
B
SW480
DLD1
0 Gy
4 Gy
relative viability
Talazoparib [μM]
C
SW480 - 0 Gy
SW480 - 4 Gy
TALA only
TALA + TRA 1 nM
TALA + TRA 2 nM
relative viability
Talazoparib [μM]

Figure S13: PARP inhibitors synergize with MEK inhibitors to enhance radiation response, related to Figure 6. A, Response/inhibition matrix derived from talazoparib - trametinib combinations for all seven tested organoid lines: Non-irradiated, irradiated, Bliss expected response, according to a model of added radiation to fixed combinations of trametinib and talazoparib, as well as Bliss excess (observed response - expected response) are shown. Data were normalized to non-irradiated DMSO controls. The complete matrices tested are shown for each drug. 2 biological replicates were analyzed for D030T, D157T, 3 replicates were analyzed for D007T, D052T, D134T and D160, and 4 replicates were analyzed for D080T. Of note, due to a pipetting error in one of the master plates, six individual wells had to be excluded from analysis of organoid lines D052T, D080T and D160T, including 3 wells containing DMSO controls and 3 wells containing drug combinations. **B,** Viability assays of CRC cell lines treated with increasing concentrations of PARP inhibitor talazoparib with and without radiation. Cell viability was determined after 60 hrs of treatment by CellTiter-Glo. **C,** Viability assays of CRC cell line SW480 treated with increasing concentrations of PARP inhibitor talazoparib in combination with selected low-dose trametinib treatments, with and without radiation. Cell viability was determined after 60 h of treatment by CellTiter-Glo. **B-C,** Data from three independent experiments are presented as mean ± SD. $^*p < 0.05$, $^{**}p < 0.01$, $^{***}p < 0.001$, two-tailed t-test.

Supplementary Tables

Table S1: Clinical characteristics of patient donors of colorectal cancer organoids, related to STAR Methods

Organoid	Sex	Location	Biopsy	T	N	M	Stage (UICC)	Grading (WHO)	Neoadjuvant Treatment	Dworak	mrTRG	Δ tumor length
D004T	f	rectum	primary	3	2	0	3	2	50,4 Gy + capecitabine	1	3	-2
D007T	m	rectum	primary	3	3	0	3	2	50,4 Gy + capecitabine	2	3	6
D027T	m	rectum	primary	4	1	1	4	2	N/A	N/A	N/A	N/A
D030T	f	colon	primary	3	0	0	2	2	N/A	N/A	N/A	N/A
D046T	f	rectum	primary	2	1	0	3	2	50,4 Gy + capecitabine	4	1	-6
D052T	m	rectum	primary	3	0	0	2	2	50,4 Gy + capecitabine	2	5	0
D073T	m	rectum	primary	3	+	0	3	N/A	50,4 Gy + FOLFOX	1	3	N/A
D080T	f	rectum	primary	3	3	0	3	1	50,4 Gy + capecitabine	1	5	1
D082T	f	rectum	primary	4	3	0	3	2	50,4 Gy + capecitabine	1	4	-1
D086T	f	rectum	primary	3	3	0	3	2	50,4 Gy + capecitabine	1	3	-1
D104T	f	rectum	primary	3	4	1	4	2	5 x 5 Gy	2	N/A	N/A
D114T	f	rectum	primary	3	3	0	3	2	50,4 Gy + capecitabine	4	2	4
D134T	f	rectum	primary	3	2	0	2	2	50,4 Gy + capecitabine	4	2	-2
D147T	m	rectum	metastasis	3	4	1	4	2	50,4 Gy + capecitabine	1	4	-2
D157T	f	colon	primary	3	4	1	4	2	N/A	N/A	N/A	N/A
D160T	m	rectum	primary	3	3	0	3	2	N/A	N/A	N/A	N/A

Abbr: T, Tumor; N, node; M, Metastasis; N/A, not available; Gy, Gray; +, positive; Dworak, Dworak pathological regression grade; mrTRG, MRI tumor regression grade.

Table S3: Library design of the clinical library, related to STAR Methods

Drug name	Category	Target	Cmax [µM]	Cmin [µM]	Cat#
5-FU	Chemotherapy	DNA-antimetabolite	100	0,160	S1209
Abemaciclib	Targeted	CDK4/6	10	0,016	S5716
Acalabrutinib	Targeted	BTK	50	0,080	S8116
Adagrasib	Targeted	KRAS G12C	50	0,080	S8884
Adavosertib	Targeted	Wee1	125	0,200	S1525
Afatinib	Targeted	EGFR, HER2, ErbB3, ERbB4	100	0,160	S1011
Alectinib	Targeted	ALK, RET	5	0,008	S2762
Alpelisib	Targeted	PI3Ka	125	0,200	S2814
Altretamine	Chemotherapy	DNA-crosslinks	50	0,080	S1278
Avapritinib	Targeted	PDGFRa D842V-mutation, c-Kit	50	0,080	S8553
Axitinib	Targeted	VEGFR1, VEGFR2, VEGFR3, PDGFR3, c-Kit	75	0,120	S1005
AZD 4547	Targeted	FGFR, VEGFR2 (KDR), IGFR, CDK2, p38	125	0,200	S2801
Belinostat	Targeted	HDAC	50	0,080	S1085
Binimetinib	Targeted	MEK1/2	25	0,040	S7007
Birinapant	Targeted	XIAP, cIAP1	25	0,040	S7015
Bleomycin	Chemotherapy	DNA-polymerase	50	0,080	S1214
Bortezomib	Targeted	proteasome	0,5	0,001	S1013
Bosutinib	Targeted	Abl/Src, PI3K/AKT/mTor, MAP/ERK, JAK/STAT3	50	0,080	S1014
Brigatinib	Targeted	ALK/ROS1, IGF-1R, FLT3, EGFR	5	0,008	S8229
Cabazitaxel	Chemotherapy	microtubules	2,5	0,004	S3022
Cabozantinib	Targeted	VEGFR2, c-Met, RET, Kit, Fit-1/3/4, Tie2, AXL	100	0,160	S1119
Capivasertib	Targeted	AKT	125	0,200	S8019
Capmatinib	Targeted	c-MET, RONb, EGFR, HER3, Wnt/b-catenin	12,5	0,020	S2788
Carboplatin	Chemotherapy	DNA-crosslinks	5	0,008	S1215
Carfilzomib	Targeted	proteasome	50	0,080	S2853
Carmustine	Chemotherapy	DNA-alkylating	75	0,120	S3669
Ceritinib	Targeted	ALK, IGF-1R, InsR, STK22d, FLT3	10	0,016	S7083
Cisplatin	Chemotherapy	DNA-crosslinks	75	0,120	S1166
Cobimetinib	Targeted	MEK1	50	0,080	S8041
Copanlisib	Targeted	PI3Ka/d	5	0,008	S2802
Crizotinib	Targeted	ALK/ROS1, c-MET	62,5	0,100	S1068
Cyclophosphamide	Chemotherapy	DNA-alkylating	75	0,120	S1217
Dabrafenib	Targeted	BRAFV600E	50	0,080	S2807
Dacarbazine	Chemotherapy	DNA-alkylating	50	0,080	S1221
Dacomitinib	Targeted	EGFR, ErbB2, ERbB4	50	0,080	S2727
Dactinomycin	Chemotherapy	DNA-Synthesis, Transcription, Topoisomerase II	75	0,120	S8964
Dasatinib	Targeted	Abl, Src, c-Kit	100	0,160	S1021
Defactinib	Targeted	FAK	37,5	0,060	S7654
Docetaxel	Chemotherapy	microtubules	2,5	0,004	S1148
Doxorubicin	Chemotherapy	DNA-topoisomerase II	50	0,080	S1208
Duvelisib	Targeted	PI3Kd/g	100	0,160	S7028
Enasidenib	Targeted	IDH-2	50	0,080	S8205
Encorafenib	Targeted	BRAFV600E	50	0,080	S7108
Entrectinib	Targeted	TRKA/B/C, ROS1, ALK	50	0,080	S7998
Epirubicin	Chemotherapy	DNA-topoisomerase	75	0,120	S1223
Erdafitinib	Targeted	FGFR, RET, CSF-1R, PDGFR-a/b, FLT4, KIT, VEGFR-2	100	0,160	S8401
Erlotinib	Targeted	EGFR	100	0,160	S7786
Etoposide	Chemotherapy	DNA-topoisomerase II	100	0,160	S1225
Everolimus	Targeted	mTor/FKBP12	25	0,040	S1120
Fedratinib	Targeted	JAK2, FLT3, Ret	50	0,080	S2736
Floxuridine	Chemotherapy	DNA-antimetabolite	50	0,080	S1299
Gedatolisib	Targeted	PI3Ka/g, mTor	5	0,008	S2628
Gefitinib	Targeted	EGFR	100	0,160	S1025
Gemcitabine	Chemotherapy	DNA-antimetabolite	12,5	0,020	S1714
Gilteritinib	Targeted	FLT3/AXL	15	0,024	S7754

Glasdegib	Targeted	Smoothened	100	0,160	S7160
Homoharringtonine	Targeted	STAT3, translation	50	0,080	S9015
Hydroxyurea	Chemotherapy	DNA-synthesis	75	0,120	S1896
Ibrutinib	Targeted	BTK	50	0,080	S2680
Idelalisib	Targeted	p110d	100	0,160	S2226
Ifosfamide	Chemotherapy	DNA-alkylating	75	0,120	S1302
Imatinib	Targeted	Abl, c-KIT, PDGFR	100	0,160	S2475
Ivosidenib	Targeted	IDH-1	50	0,080	S8206
Ixabepilone	Chemotherapy	microtubules	2,5	0,004	S7930
Ixazomib	Targeted	proteasome	2,5	0,004	S2180
Lapatinib	Targeted	EGFR, ErbB2 (HER2)	100	0,160	S2111
Larotrectinib	Targeted	TRKA/B/C	50	0,080	S5860
Lenalidomide	Targeted	CRBN, TNF-a	100	0,160	S1029
Lenvatinib	Targeted	VEGFR, FGFR, PDGFR, RET, c-Kit	75	0,120	S1164
LGK974	Targeted	PORCN	100	0,160	S7143
Lomustine	Chemotherapy	DNA-alkylating	75	0,120	S1840
Lorlatinib	Targeted	ALK/ROS1	50	0,080	S7536
Lurbinectedin	Chemotherapy	DNA-binding	5	0,008	S9603
Mechlorethamine	Chemotherapy	DNA-alkylating	75	0,120	S4252
Melphalan	Chemotherapy	DNA-alkylating	30	0,048	S8266
Methotrexat	Chemotherapy	DNA-antimetabolite	50	0,080	S1210
Midostaurin	Targeted	PKCa/b/g, Syk, c-Kit, c-Fgr, c-Src, FLT3, PDFRb, VEGFR1/2	50	0,080	S8064
Mitomycin C	Chemotherapy	DNA-crosslinks	12,5	0,020	S8146
Mitoxantrone	Chemotherapy	DNA-crosslinks, topoisomerase II	10	0,016	S1889
MK-8776	Targeted	Chk1	37,5	0,060	S2735
Napabucasin	Targeted	STAT3	25	0,040	S7977
Navitoclax	Targeted	Bcl-2, Bcl-xL, Bcl-w	75	0,120	S1001
Neratinib	Targeted	EGFR/HER2	25	0,040	S2150
Nilotinib	Targeted	Bcr-Abl	50	0,080	S1033
Niraparib	Targeted	PARP1/2	100	0,160	S2741
Nutlin3a	Targeted	p53/MDM2	100	0,160	S8059
Octreotide	Targeted	Somatostatine analogon	20	0,032	P1017
Olaparib	Targeted	PARP1/2	100	0,160	S1060
Osimertinib	Targeted	EGFR	100	0,160	S7297
Oxaliplatin	Chemotherapy	DNA-crosslinks	75	0,120	S1224
Paclitaxel	Chemotherapy	microtubules	2,5	0,004	S1150
Palbociclib	Targeted	CDK4/6	25	0,040	S1116
Panobinostat	Targeted	HDAC	2,5	0,004	S1030
Pazopanib	Targeted	VEGFR1/2/3, PDGFR, FGFR, c-Kit, c-Fms	100	0,160	S3012
PD0166285	Targeted	Wee1, Chk1	25	0,040	S8148
Pemetrexed	Chemotherapy	DNA-antimetabolite	50	0,080	S1135
Pemigatinib	Targeted	FGFR1/2/3/4	50	0,080	S0088
Pexidartinib	Targeted	CSF-1R, Kit, FLT3	100	0,160	S7818
Pomalidomide	Targeted	CRBN, TNF-a	100	0,160	S1567
Ponatinib	Targeted	Abl, PDGFR, VEGFR2, FGFR1, Src	125	0,200	S1490
Poziotinib	Targeted	HER1,2,4	50	0,080	S7358
Pralsetinib	Targeted	c-RET	50	0,080	S8716
PRI-724	Targeted	b-catenin/CBP/p300	75	0,120	S8968
Regorafenib	Targeted	VEGFR1/2/3, PDGFR, Kit, Ret, Raf-1	100	0,160	S1178
Ribociclib	Targeted	CDK4/6	25	0,040	S7440
Ripretinib	Targeted	PDGFR-a, c-Kit	75	0,120	S8757
Romidepsin	Targeted	HDAC1/2	75	0,120	S3020
Rucaparib	Targeted	PARP	100	0,160	S1098
Ruxolitinib	Targeted	JAK1/2	100	0,160	S1378
Selinexor	Targeted	CRM1/Exportin1	100	0,160	S7252
Selpercatinib	Targeted	c-RET	75	0,120	S8781
Selumetinib	Targeted	MEK1/2	12,5	0,020	S1008
SN-38 (Irinotecan)	Chemotherapy	DNA-topoisomerase I	5	0,008	S4908
Sonidegib	Targeted	Smoothened	100	0,160	S2151
Sorafenib	Targeted	BRAF, RAF, VEGFR2/3, PDGFR-b, Fit-3, c-Kit	100	0,160	S7397

Streptozocin	Chemotherapy	DNA-alkylating	75	0,120	S1312
Sunitinib	Targeted	VEGFR2, PDGFR-b	50	0,080	S7781
Talazoparib	Targeted	PARP1/2	50	0,080	S7048
TAS-102	Chemotherapy	DNA-antimetabolite	100	0,160	S8539
Taselisib	Targeted	PI3Ka/d/g	25	0,040	S7103
Tazemetostat	Targeted	EZH2	75	0,120	S7128
Temozolomide	Chemotherapy	DNA-alkylating	75	0,120	S1237
Temsirolimus	Targeted	mTOR	75	0,120	S1044
Thiotepa	Chemotherapy	DNA-alkylating	75	0,120	S1775
Topotecan	Chemotherapy	DNA-topoisomerase I	5	0,008	S9321
Trametinib	Targeted	MEK1/2	2,5	0,004	S2673
Tucatinib	Targeted	HER2	100	0,160	S8362
Valrubicin	Chemotherapy	DNA-topoisomerase II	20	0,032	S9522
Vandetanib	Targeted	VEGFR2, VEGFR3, EGFR, RET	50	0,080	S1046
Vemurafenib	Targeted	BRAFV600E	100	0,160	S1267
Venetoclax	Targeted	Bcl-2	25	0,040	S8048
Vinblastin	Chemotherapy	microtubules	0,5	0,001	S4505
Vinorelbine	Chemotherapy	microtubules	2,5	0,004	S4269
Vismodegib	Targeted	Smoothened	100	0,160	S1082
Vistusertib	Targeted	mTOR	25	0,040	S2783
Volasertib	Targeted	PLK1	5	0,008	S2235
Vorinostat	Targeted	HDAC	100	0,160	S1047
XAV-939	Targeted	Wnt/b-catenin	100	0,160	S1180
YM155	Targeted	Survivin	5	0,008	S1130
Zanubrutinib	Targeted	BTK	50	0,080	S8791

Table S4: Primers for quantitative PCR, related to STAR Methods

Target gene	Species	Forward primer	Reverse primer
BRCA1	human	TTGTTGATGTGGAGGAGCAA	GATTCCAGGTAAGGGGTTCC
BRCA2	human	GAAAATCAAGAAAAATCCTTAAAG GCT	GTAATCGGCTCTAAAGAA ACATGATG
EGR1	human	AGCCCTACGAGCACCTGAC	GGTTTGGCTGGGGTAACTG
DDB2	human	CTCCTCAATGGAGGGAACAA	GTGACCACCATTCGGCTACT
DUSP4	human	GGCGGCTATGAGAGGTTTTCC	TGGTCGTGTAGTGGGGTCC
RAD21	human	AATTTGGCTAGCGGCCCAT	TGTCCGTAATGCCATTTTCACC
RAD51	human	GGTGAAGGAAAGGCCATGTA	GGGTCTGGTGGTCTGTGTT
RAD51B	human	GCACAAAGGTCTGCTGATTTC	CCCATGTTGGTGGGTAATGT
SPRY2	human	CCTACTGTCGTCCCAAGACCT	GGGGCTCGTGCAGAAGAAT
UBC	human	CTGATCAGCAGAGGTTGATCT TT	TCTGGATGTAGTCAGACAGG
XRCC2	human	TCACCTGTGCATGGTGATATT	TTCCAGGCCACCTTCTGATT
