# Supplementary material for: Combined MEK and PARP inhibition enhances radiation response in rectal cancer
Source: Cell Rep Med. 2025 Aug 8;6(8):102284. doi: 10.1016/j.xcrm.2025.102284 (PMC12432356; doi:10.1016/j.xcrm.2025.102284)
Supplement: Document S2. Article plus supplemental information [file mmc3.pdf]

Cell Reports Medicine

Article

Combined MEK and PARP inhibition enhances radiation response in rectal cancer

Graphical abstract

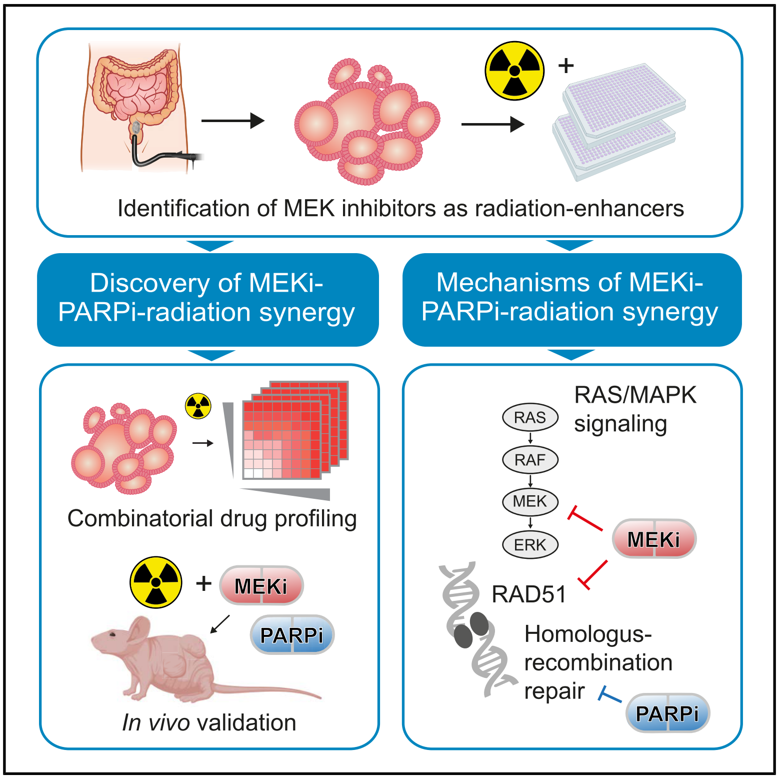

Authors

Qiyun Xiao, Julian E. Riedesser, Theresa Mulholland, ..., Matthias P. Ebert, Tianzuo Zhan, Johannes Betge

Correspondence

tianzuo.zhan@medma.uni-heidelberg.de (T.Z.),
j.betge@dkfz.de (J.B.)

In brief

Xiao et al. use patient-derived rectal cancer organoids to identify that MEK inhibitors enhance radiation sensitivity by downregulating the DNA damage response protein RAD51. They find synergy with combined PARP inhibition in different preclinical models, thereby offering a promising regimen and potential future alternative to conventional chemoradiation in locally advanced rectal cancer.

Highlights

- Clinical radiosensitivity can be modeled with a rectal cancer organoid assay
- A drug-radiation screen identifies MEK inhibitors as potent irradiation enhancers
- MEK inhibitors downregulate the DNA damage response protein RAD51
- Combined MEK-PARP-radiation shows efficacy in organoid and xenograft models

Xiao et al., 2025, Cell Reports Medicine *6*, 102284
August 19, 2025 © 2025 The Author(s). Published by Elsevier Inc.
https://doi.org/10.1016/j.xcrm.2025.102284

Cell Reports Medicine

CellPress
OPEN ACCESS

Article

Combined MEK and PARP inhibition enhances radiation response in rectal cancer

Qiyun Xiao,[1,2,16,17] Julian E. Riedesser,[1,3,17] Theresa Mulholland,[1,3,17] Zhenchong Li,[1,3] Jonas Buchloh,[1,3] Philipp Albrecht,[1,3] Xinchen Yang,[1] Moying Li,[1] Nachiyappan Venkatachalam,[1] Olga Skabkina,[1] Anna Klupsch,[1] Ella Eichhorn,[1] Li Wang,[1] Sebastian Belle,[1] Nadine Schulte,[1] Daniel Schmitz,[4] Matthias F. Froelich,[5] Kyrhatii Trikhirhisthit,[3] Erica Valentini,[6] Kim E. Boonekamp,[6] Yvonne Petersen,[1,3] Thilo Miersch,[6] Elke Burgermeister,[1] Carsten Herskind,[7,14] Marlon R. Veldwijk,[7,14] Christoph Brochhausen,[8] Robert Ihnatko,[9] Jeroen Krijgsveld,[9] Ina Kurth,[10,12] Yuxing Zhu,[13] Yanni Ma,[13] Ke Cao,[13] Michael Boutros,[6,11,12] Matthias P. Ebert,[1,14,15,16] Tianzuo Zhan,[1,14,15,16,17,*] and Johannes Betge[1,3,12,14,15,18,17,*]

[1]Department of Medicine II, University Medical Center Mannheim, Medical Faculty Mannheim, Heidelberg University, Mannheim, Germany
[2]Department of Medical Oncology, Sun Yat-sen University Cancer Center, State Key Laboratory of Oncology in South China, Guangdong Provincial Clinical Research Center for Cancer, Sun Yat-sen University, Research Unit of Precision Diagnosis and Treatment for Gastrointestinal Cancer, Chinese Academy of Medical Sciences, Guangzhou, China
[3]Junior Clinical Cooperation Unit Translational Gastrointestinal Oncology and Preclinical Models, German Cancer Research Center (DKFZ), Heidelberg, Germany
[4]Department of Gastroenterology and Infectiology, Helios Kliniken Schwerin, University Campus of Medical School Hamburg (MSH), Schwerin, Germany
[5]Institute of Clinical Radiology and Nuclear Medicine, University Medical Center Mannheim, Medical Faculty Mannheim, Heidelberg University, Mannheim, Germany
[6]Division of Signaling and Functional Genomics, German Cancer Research Center (DKFZ), Heidelberg, Germany
[7]Department of Radiation Oncology, University Medical Center Mannheim, Medical Faculty Mannheim, Heidelberg University, Mannheim, Germany
[8]Institute of Pathology, University Medical Center Mannheim, Medical Faculty Mannheim, Heidelberg University, Mannheim, Germany
[9]Division of Proteomics of Stem Cells and Cancer, German Cancer Research Center (DKFZ), Heidelberg, Germany
[10]Division of Radiooncology/Radiobiology, German Cancer Research Center (DKFZ), Heidelberg, Germany
[11]Institute of Human Genetics, Medical Faculty Heidelberg, Heidelberg University, Heidelberg, Germany
[12]German Cancer Consortium (DKTK), Heidelberg, Germany
[13]Department of Oncology, Third Xiangya Hospital, Central South University, Changsha, China
[14]DKFZ Hector Cancer Institute at University Medical Center Mannheim, Mannheim, Germany
[15]Mannheim Cancer Center, Medical Faculty Mannheim, Heidelberg University, Mannheim, Germany
[16]Molecular Medicine Partnership Unit, European Molecular Biology Laboratory, Heidelberg, Germany
[17]These authors contributed equally
[18]Lead contact
*Correspondence: tianzuo.zhan@medma.uni-heidelberg.de (T.Z.), j.betge@dkfz.de (J.B.)
https://doi.org/10.1016/j.xcrm.2025.102284

SUMMARY

Rectal cancer is frequently diagnosed at a locally advanced stage and treated by neoadjuvant chemoradiation. Current efforts to improve treatment outcome are focused on intensifying neoadjuvant chemotherapy, which is associated with higher levels of toxicity. To discover alternative strategies, we establish patient-derived rectal cancer organoids that reflect clinical radiosensitivity and use these organoids to screen 1,596 drug-radiation combinations. We find that inhibitors of rat sarcoma virus/mitogen-activated protein kinase (RAS-MAPK) signaling, especially mitogen-activated protein kinase kinase (MEK) inhibitors, strongly enhance radiation response. Mechanistically, MEK inhibitors suppress radiation-induced activation of RAS-MAPK signaling and selectively downregulate RAD51, a component of the homologous recombination DNA repair pathway. Through testing drug-drug-radiation combinations in organoids and cell lines, we identify that a combined poly ADP-ribose polymerase (PARP) and MEK inhibition can further enhance radiosensitivity of colorectal cancers, which we confirm in mouse xenograft models. Our data support clinical testing of MEK and PARP combination therapy with radiation in locally advanced rectal cancers as an alternative to chemoradiation.

© 2025 The Author(s). Published by Elsevier Inc.
This is an open access article under the CC BY license (http://creativecommons.org/licenses/by/4.0/).

INTRODUCTION

Colorectal cancer (CRC) stands as a leading cause of cancer-related mortality.[1] Over one-third of CRC originates in the rectum, often presenting at a locally advanced stage, which is defined as Union for International Cancer Control (UICC) classification T3/T4 (invasion beyond muscular layers) and/or node-positive disease. The current standard of care for most locally advanced rectal cancers is neoadjuvant chemoradiotherapy, followed by surgical resection of the tumor.[2] Introduction of neoadjuvant chemoradiotherapy led to improved local tumor control rates in clinical trials.[3] The most commonly applied regimens include a combination of long-course radiation with intravenous or oral fluoropyrimidine and, more recently, the addition of consolidation chemotherapy with 5-fluorouracil and oxaliplatin, termed total neoadjuvant therapy (TNT).[4] The response to neoadjuvant chemoradiotherapy varies significantly between individuals, ranging from complete responses without detectable tumor residues to non-response. Patients who achieve complete response have an improved overall survival and may avoid surgical resection of the rectum, which can be severely debilitating. While standard neoadjuvant chemoradiotherapy has resulted in complete response rates of ∼15%,[5] intensified regimens such as TNT can significantly increase the rates of complete response. However, this success is achieved at the expense of increased toxicity, which is caused by the broad mode of action of conventional chemotherapeutic agents, notably neurotoxicity induced by oxaliplatin.[6] Furthermore, resistance to radiotherapy in rectal cancer has been linked to several molecular mechanisms, including enhanced DNA damage repair, apoptosis escape, regulation of cancer stemness pathways, metabolic reprogramming, and others.[7] Approaches that target such mechanisms or tumor-specific alterations to enhance radiosensitivity have not been introduced into clinical practice, despite promising preclinical results with different small-molecule drugs and antibodies.[8] One of the underlying reasons is the absence of suitable tumor models that adequately reflect the biological characteristics of rectal cancers, which are dominated by particularly high frequencies of RAS-MAPK (rat sarcoma virus/mitogen-activated protein kinases) and WNT pathway mutations.[9] To this end, traditional 2D cell culture models often fail to fully capture the complexity of human rectal cancer biology and therapeutic responses. Recently, patient-derived organoids have been introduced as models that can recapitulate the tumor biology of many cancer types and their response to different therapeutic modalities.[10] In particular, studies have shown associations of rectal cancer organoids' response to radiation with response of corresponding tumors in patients.[11–13] So far, however, rectal cancer organoid platforms have not been exploited to systematically screen for drug candidates that can enhance the response of rectal cancers to radiotherapy.

In this study, we establish a rectal cancer organoid platform that recapitulates clinical radiosensitivity and use it to perform large-scale drug screens to identify drugs synergizing with radiation therapy. We observed that inhibitors of the RAS-MAPK pathway, in particular mitogen-activated protein kinase kinase 1/2 (MEK1/2) inhibitors, can strongly increase the sensitivity of rectal cancer organoids and CRC cell lines to radiation. Mechanistically, we find that radiotherapy induces an activation of RAS-MAPK signaling, which could be suppressed by MEK inhibition. Moreover, MEK inhibitors (MEKi) downregulate RAD51 recombinase in protein levels, a key component of the DNA repair machinery. Accordingly, we find that MEKi synergize with poly ADP-ribose polymerase 1/2 (PARP1/2) inhibitors in reducing tumor cell viability, and the combination of these two agents can further enhance the effectiveness of radiotherapy in CRC cell lines, organoids, and murine xenograft models.

RESULTS

An organoid platform recapitulates essential aspects of rectal cancer

To model cancer biology and identify treatment options for rectal cancer, we established an organoid-based platform and living biobank. Organoids were generated from pre-treatment endoscopic biopsies from patients with rectal cancers of different UICC/tumor-node-metastasis stages (Figure 1A; Table S1). They showed heterogeneous morphologies and molecular alterations that are characteristic for CRC (Figure 1B).[14] Previous studies have demonstrated associations between clinical signs of radiation response in patients with rectal cancer and radiation response of corresponding rectal cancer organoids, using various protocols.[11–13] We established a standardized, robot-assisted radiation protocol for organoids, based on our previously published high-throughput screening platform.[15] By exposing tumor organoid cultures to different doses of radiation, we observed a clear dose dependency of organoid viability and a high degree of variation in radiosensitivity between different patient donors (Figures 1C–1F, S1A, and S1B). The variation in response was not explained by frequent mutations found in the organoids, consistent with previous findings (Figure S1C).[9] Clinical response to chemoradiotherapy determined by magnetic resonance imaging (MRI) regression grading showed a significant association (almost perfect except for one outlier) with the radiation response of corresponding patient-derived rectal cancer organoids (Figures 1D–1F). Additionally, histopathological analysis of regression grade in post-radiation tumor resection specimens, as well as analysis of post-treatment changes in tumor length in MRI, also showed a strong association (although not statistically significant) with organoid response (Figures 1D–1F and S1D). Finally, endoscopic assessment of therapy response corresponded to the radiosensitivity of organoids in our assay (representative clinical images shown in Figure 1F). These findings are in concordance with observations of previous studies and support the clinical and functional relevance of our organoid radiation assay in modeling the radiosensitivity of rectal cancers.

High-throughput screening in rectal cancer organoids identifies RAS-MAPK signaling inhibitors as enhancers of radiation response

Radiation is usually combined with fluoropyrimidines as chemotherapeutic agents for the treatment of locally advanced rectal

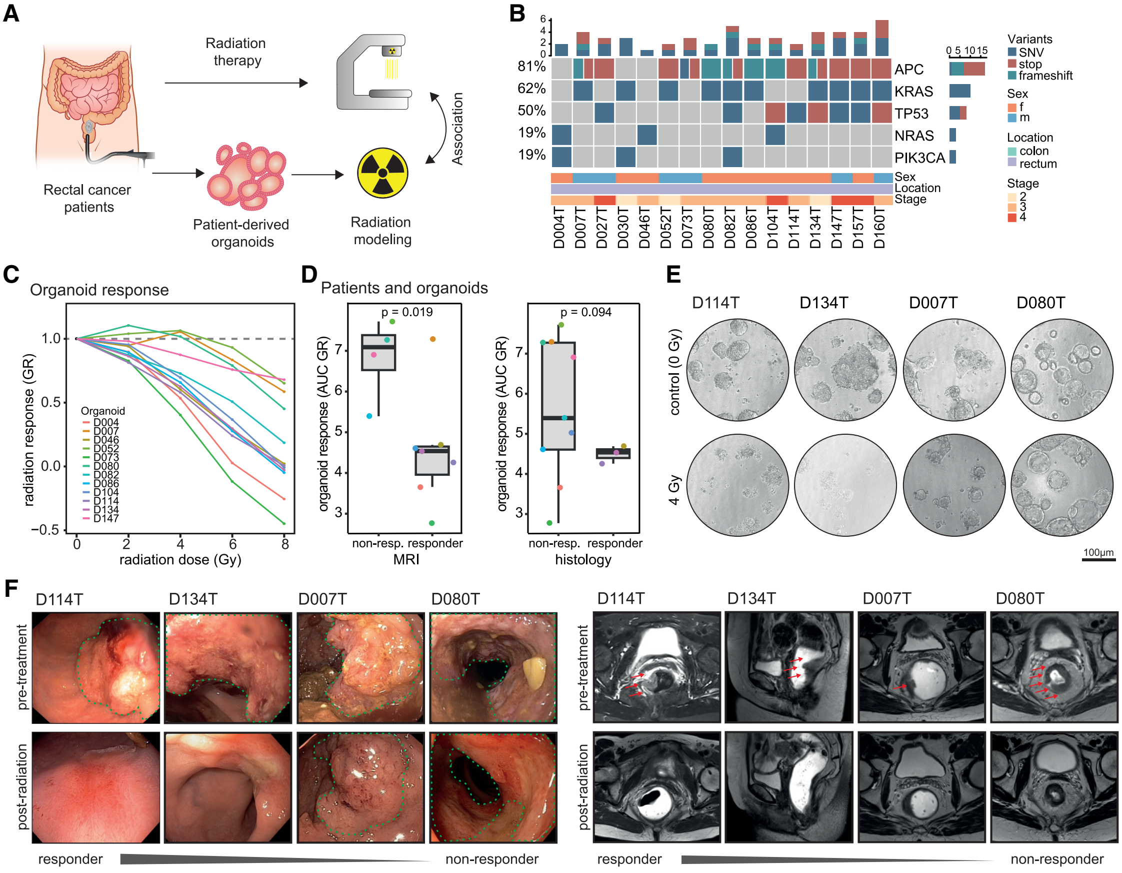

Figure 1. An organoid platform recapitulates essential aspects of rectal cancer

(A) Schematic illustration of the rectal cancer organoid platform and approach for association of patient and organoid response.

(B) Driver mutations identified in patient-derived organoids.

(C) Response of rectal cancer organoids to increasing doses of radiation.

(D) Analysis of organoid response to radiation according to donor patients' rectal cancer response to radiation therapy assessed by MRI-based regression grading (left, 11 evaluable cases, two-tailed Student's t test) and histopathological examination (right, 12 evaluable cases, two-tailed Welch's t test).

(C and D) Each data point represents the mean of 3–6 biological replicates tested per organoid line.

(E) Representative bright-field images of rectal cancer organoid cultures undergoing radiation. Scale bars: 100 μM.

(F) Representative endoscopy (left) and MRI (right) images from selected patients pre-treatment and post radiation therapy, sorted according to organoid response to radiation therapy. Green dotted lines (left) and red arrows (right) indicate location of rectal cancer.

See also Table S1; Figure S1.

cancer. While fluoropyrimidines have been reported to exhibit radiosensitizing properties,[16] their modes of action are broad and non-selective. Hence, drugs with stronger radiosensitizing effects are needed to achieve deeper responses, which may spare patients from debilitating rectal resection. To screen for synergistic treatment combinations of radiation with medical therapies, we used our rectal cancer organoid biobank and further developed our semi-automated radiation workflow toward a high-throughput assay for combined drug and radiation therapy screening. Within this workflow, organoids underwent radiation and drug treatments in 384-well format in a viability assay over 9 days (Figure 2A). We used ΔAUCs (area under the dose-response curve of non-irradiated-irradiated conditions) as a simple metric to screen for drugs that could enhance the radiation effect. Normalizing perturbations to the plate-specific dimethyl sulfoxide (DMSO) controls (i.e., separately for irradiated or non-irradiated plates) revealed radio-enhancing effects and avoided overestimation of effects and bias between the screened plates. We first used two organoid lines that were resistant to radiation therapy (D080T and D007T, compare Figure 1) and applied radiation treatment in combination with a library of 224 drugs, mostly kinase inhibitors, in four concentrations (Figures 2B and S2A–S2C). In both organoid lines, we identified several kinase inhibitors that enhanced radiation effects and also compounds that diminished tumor cell killing upon radiation treatment (Figures 2C–2F). The enhancing effects were generally stronger in organoid line D080T than in D007T (Figures 2C and 2E). Many compounds that conferred high radiation-induced killing in both organoid lines belonged to the class of RAS-MAPK signaling inhibitors (particularly inhibitors of epidermal growth factor receptor [EGFR] and MEK, Figures 2D and 2F). To validate these findings and to identify additional

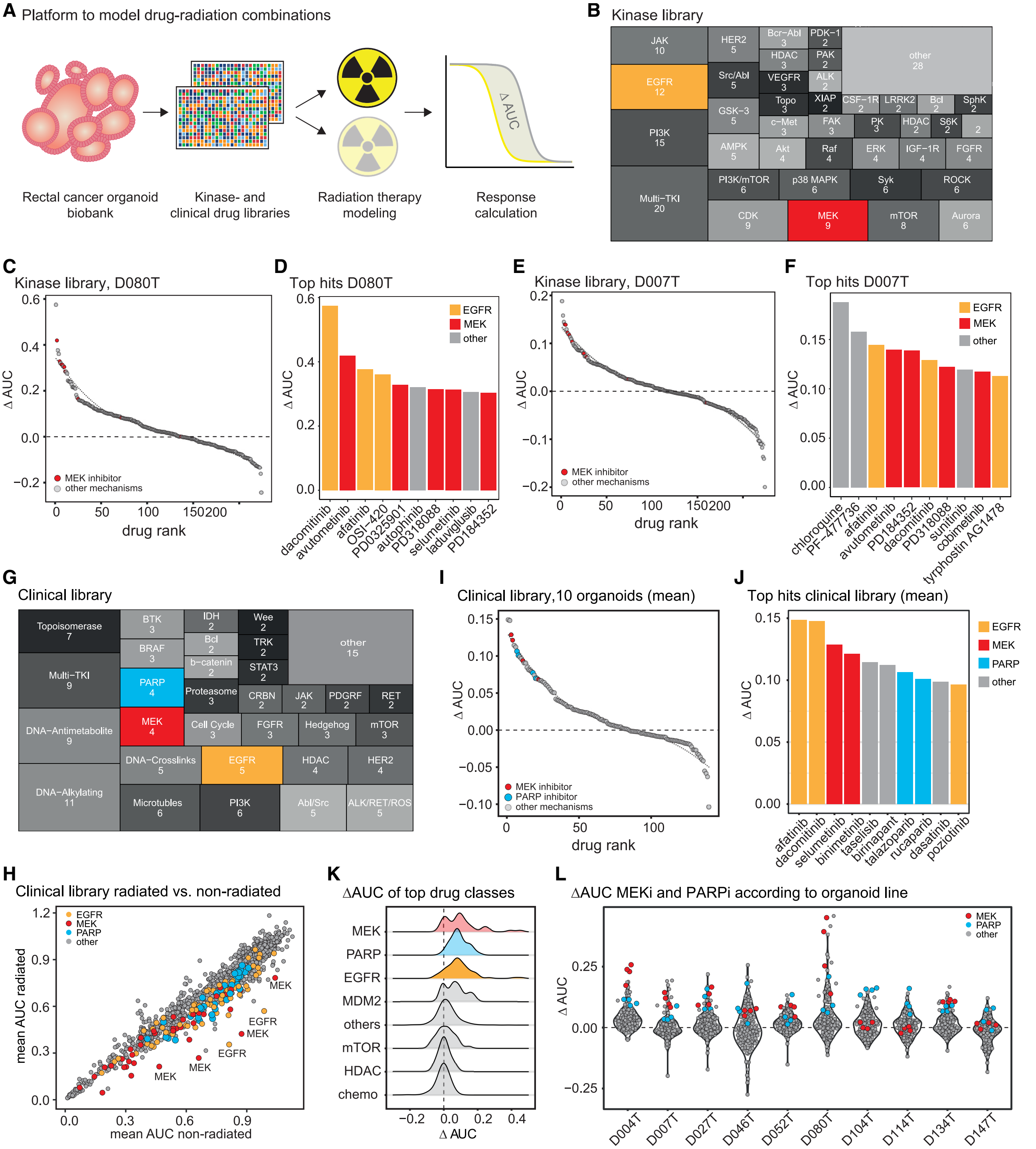

Figure 2. Drug screening identifies RAS-MAPK pathway inhibitors to synergistically enhance radiation in rectal cancer organoids

(A) Schematic representation of the drug-radiation screening workflow. Rectal cancer organoids were seeded in 384-well plates; drug perturbations with 4–5 concentrations and radiation (2–4 Gy) were performed on day 3, before viability was measured on day 9 after seeding. Interactions of drugs and radiation were analyzed by calculating the difference between areas under the dose-response curves (ΔAUC values) between irradiated and non-irradiated conditions, each normalized to respective irradiated and non-irradiated DMSO controls on the same plates.

(B) Composition of the kinase drug library with 224 drugs, tested in 4 concentrations in two organoid lines.

(C) Ranking of differential effects of kinase inhibitors with or without radiation in tumor organoid D080T.

(D) Top 10 hits with highest radiation enhancement (ΔAUC values) in the kinase library screen of organoid line D080T.

(legend continued on next page)

clinically available radiation-enhancing drugs with the potential for fast clinical translation, we performed further screening experiments. We used a library containing 140 cancer drugs, mostly Food and Drug Administration (FDA) approved, that could be meaningfully modeled in organoid experiments (i.e., including drugs with a mechanism directly targeting cancer cells, Figures 2G and S2D–S2H). This library included every drug in five concentrations and was tested in 10 different rectal cancer organoid models, including both RAS-mutated and wild-type cases (Figures 2H–2L). Irradiation was done with 4 Gy for most lines and 2 Gy for lines with higher radiation sensitivity. Most drugs showed similar responses in irradiated and non-irradiated conditions, while mainly inhibitors of the RAS-MAPK signaling pathway recurrently demonstrated enhanced killing together with radiation (Figure 2H). Ranking drugs according to ΔAUC demonstrated that RAS-MAPK (EGFR and MEK) pathway inhibitors, as well as PARP inhibitors (PARPi), which are known radiosensitizers in different tumors,[17] were among the strongest hits (ΔAUC ± SEM trametinib = 0.0934 ± 0.0286, afatinib = 0.1488 ± 0.0388, talazoparib = 0.1064 ± 0.0211, Figures 2I–2K, S3A, and S3B). The degree of radiosensitization varied between the different organoid models, likely representing the molecular heterogeneity of rectal cancers (Figures 2L and S3B–S3D). With respect to MEKi, seven out of ten organoid lines showed enhanced tumor organoid killing to varying degrees when combined with radiation (Figure 2L). In six of them, we observed a significantly increased ΔAUC of MEKi trametinib in irradiated vs. non-irradiated condition compared to the average ΔAUCs of all tested drugs (Figure S3D). EGFR inhibitors exhibited similar trends but not congruent profiles of radio-enhancement across different organoid lines (3 of the lines showed significantly increased ΔAUCs for afatinib, Figure S3D), while the profiles were distinct for PARPi (5 of 10 lines had significantly enhanced ΔAUCs upon treatment with talazoparib; Figures 2L and S3D). We also tested associations of common molecular alterations in organoid lines with the level of radiosensitization. We found that organoid lines with wild-type *TP53* status generally showed drug responses that were more strongly modifiable by radiation, especially with MEKi, while no association of radiation enhancement was observed with RAS mutation status (Figures S4A and S4B).

In conclusion, high-throughput drug-radiation combination screens with both kinase and clinical libraries independently showed a strong enhancement of radiation with inhibitors of RAS-MAPK signaling, especially MEKi, in the majority of tested organoid lines.

MEK inhibition is synergistic with radiation in CRC cell lines and organoids

Among the identified drug candidates, MEKi showed the strongest enhancement by radiation. MEKi targets RAS-MAPK signaling downstream of oncogenic RAS mutations, which are highly prevalent in rectal cancers.[9] In two previously tested organoid lines (D080T and D007T), radiation with 4 Gy combined with the FDA-approved MEKi trametinib resulted in a decrease of cell viability, which was significantly stronger in combination with radiation treatment (Figures S5A and S5B). To prove a synergistic effect of MEKi with radiation, we calculated the expected combination response of both perturbations for each tested concentration using a Bliss independence model, as recently reported for drug-drug combinations.[18] This revealed a clear excess of the experimentally observed combination response over the expected response, proving synergy between radiation and MEKi. Microscopy images of organoids showed corresponding phenotypes, with a reduction of organoid size and number after combination therapy (Figures S5A and S5B). Replicative cell death is a major cause for the antineoplastic effect of radiotherapy. To test radiation effects over several cycles of cell proliferation, we performed complementary experiments in CRC cell lines using viability assays and gold-standard colony forming assays (S5C-E, S6A). We selected three commonly used CRC cell lines SW480, DLD1, and HCT116 with different genetic backgrounds and degrees of intrinsic radiosensitivity (Figure S6A). Selection was also based on the presence of Kirsten rat sarcoma virus (KRAS) mutations in all three cell lines, as KRAS mutations are highly prevalent in rectal cancers and we assumed that MEK1/2 inhibition would be more potent in models with activated RAS-MAPK signaling.[9] Radiation was performed using sublethal doses of 2–4 Gy, depending on the intrinsic radiosensitivity of the cell lines (Figure S6A). Measurement of cell viability 5–6 days post radiation and in the presence of different concentrations of trametinib showed enhanced antineoplastic effects with combination therapy in all three cell lines (S5C-D). The level of radio-enhancement differed between the lines but was uniformly observed at low nanomolar concentrations of trametinib. Again, comparing the observed combination response with the expected response according to the Bliss independence model revealed a clear excess over the Bliss model in all cell lines (Figure S5D). The enhancing effect of combined MEKi and radiation was confirmed in long-term colony-forming assays (10–12 days of treatment) (Figure S5E). We also observed sensitizing effects for three

(E) Ranking of differential effects of kinase inhibitors with or without radiation in cancer organoid D007T.
(F) Top 10 hits with strongest radiation enhancement (ΔAUC values) in the kinase library screen of organoid line D007T.
(C–F) Mean values of two biological replicates are shown.
(G) Composition of the clinical cancer library consisting of 140 drugs. The drugs were administered in 5 concentrations, and 10 organoid lines were tested.
(H) Mean area under the curve of all drugs tested in the clinical library in radiated vs. non-radiated conditions.
(I) Ranking the mean differential effects of clinical cancer drugs with or without radiation in 10 rectal cancer organoids.
(J) Top 10 hits with strongest radiation enhancement (mean ΔAUC values) in the clinical library screen with ten rectal cancer organoids.
(K) Distribution of ΔAUCs of selected groups of inhibitors.
(L) ΔAUCs of individual organoid lines, ΔAUCs of MEKi and PARPi are highlighted.
(I–K) Mean values of 10 tested organoid lines are shown.
(H and L) Each data point represents the mean ΔAUC value of two biological replicates tested for each organoid line.
See also Figures S2–S7.

pharmacological inhibitors of the RAS-MAPK pathway, targeting EGFR, KRAS:SOS1 (Son of Sevenless 1), and extracellular signal-regulated kinase 1/2 (ERK1/2) (Figures S6B–S6F). Compared to MEKi, the radio-enhancing effect of the three compounds was weaker and more cell line dependent. When compared to the murine double minute 2 inhibitor and previously reported radiosensitizer nutlin-3a,[19] MEKi could achieve similar sensitizing effects but at much lower drug concentrations (Figures S7A and S7B) in *TP53* wild-type HCT116 cells. Of note, nutlin-3a also showed radiosensitizing effects in our organoid assays, as compared to MEKi (Figure S7C). These results indicate that targeting aberrant RAS-MAPK signaling, especially by MEKi, significantly increases cellular response to radiation in CRC.

Radiation induces RAS-MAPK signaling in CRC cell lines and organoids

To determine mechanisms underlying the radiosensitizing effects of MEKi, we first assessed the activity of RAS-MAPK signaling after irradiation by measuring pERK levels, as the pathway has previously been associated with radiation response in different tumor models.[20] We observed that radiotherapy induced a transient increase in ERK phosphorylation in DLD1 and SW480 cell lines but not in HCT116 (Figures 3A and S8A). The exact onset of RAS-MAPK activation differed between cell lines and was observed most consistently between day 3 and 6 post radiation (Figure 3A). Activation of RAS-MAPK signaling was also demonstrated at the level of target genes, as irradiation increased the expression of sprouty RTK signaling antagonist 2 or dual specificity phosphatase 4 in CRC cell lines (Figure 3B) and organoids (Figure S8B). Expression profiling of three patient-derived CRC organoid lines after irradiation with 4 Gy showed a number of differentially expressed genes, some of them related to the RAS-MAPK signaling pathway (Figures 3C and S8C). Using pathway enrichment analysis with Molecular Signatures Database HALLMARK gene sets,[21] we found that, for instance, “KRAS SIGNALING UP” was among the significantly upregulated gene sets in irradiated organoids, in addition to signatures such as apoptosis, P53_Pathway, and several inflammatory pathways (Figures 3D and S8D). We found that concomitant treatment with trametinib potently repressed basal and radiation-induced increase in pERK levels and expression of target genes of RAS-MAPK signaling in CRC cell lines (Figures 3E and 3F). This finding was corroborated in two CRC organoid lines, as MEK inhibition markedly suppressed ERK phosphorylation induced by radiation (Figure 3G). In summary, our results suggest that activation of RAS-MAPK signaling presents a mechanism of cellular adaptation of CRC to radiation. Targeting the pathway with MEKi could abolish this adaptive activation, thus providing a mechanism by which the drug sensitizes CRC cells to radiation.

MEK inhibition interferes with DNA damage response via repression of RAD51

The DNA damage response pathway is activated upon radiation-induced DNA double-strand breaks (DSBs). We assessed if targeting MEK1/2 affects this process by first measuring the formation of DSBs upon radiation and the kinetics of their resolution in the presence of the inhibitor. As shown in Figures 4A and 4B, radiation rapidly caused the formation of gamma histone 2AX (γH2AX)-positive foci in the nucleus of CRC cells, which decreased over time. Concomitant treatment with MEKi directly after irradiation neither caused an increase in the number of foci per nuclei nor changed the speed of their resolution (Figure 4B). This observation was confirmed by immunoblot analysis of p-γH2AX levels, which are increased upon irradiation but not reduced by MEKi (Figure 4C). Next, we analyzed if subsequent steps of the DNA repair pathway are affected by MEKi. DSBs can be repaired by two distinct pathways of the DNA repair machinery,[23] and we first measured transcript levels of main components of both pathways. We found that radiation upregulated the transcript levels of many DNA repair genes such as DNA damage-binding protein 2 and X-ray repair cross complementing (*XRCC*) 2 in CRC cell lines (Figures S9A and S9B), consistent with observations from previous studies.[24] We then performed global proteomics profiling of three CRC cancer cell lines. A small set of proteins were strongly downregulated by MEKi, including RAD51, a central component of the homologous recombination DNA repair pathway, which was reduced in all three CRC lines. In DLD1, we observed a marked decrease, while in the other two cell lines, the protein was nearly absent after trametinib treatment (Figures 4D, S9C, and S9E).[25] Interestingly, protein levels of other components crucial for the repair of DSBs remained mostly unchanged, including breast cancer 1 (BRCA1), PARP2, or ataxia telangiectasia mutated (Figures 4D; S9E). Loss of RAD51 upon MEKi was further confirmed in all CRC cell lines and three cancer organoids lines by immunoblot, showing a dose-dependent decrease of RAD51, both in the presence and absence of radiation (Figures 4E, 4F, S10A, and S10B). MEKi-induced reduction of RAD51 began approximately 12 h after addition of trametinib (Figure S10C) and was not caused by a transcriptional repression (Figure 4G). Of note, treatment of CRC cell lines with other inhibitors of the RAS-MAPK pathway at different levels (EGFR inhibition, ERK1/2 inhibition) did not lead to changes in RAD51 levels, suggesting that the mechanism is specific to MEKi (Figure S10D). Co-treatment with two proteasomal inhibitors did not rescue MEKi-induced loss of RAD51, indicating that MEKi elicits proteasome-independent mechanisms to reduce RAD51 levels (Figure S10E). This result was corroborated by cycloheximide chase assays, which did not show a significant acceleration of RAD51 loss upon blockage of *de novo* protein synthesis (Figure S10F). We also observed that radiation itself increased RAD51 protein levels within 24 h, and in some lines, such as DLD1, also at later time points (day 6) (Figures 4E and S10G). Hence, we hypothesized that functional depletion of RAD51 would increase radiosensitivity in our CRC models. To this end, we used RNAi to efficiently knock down RAD51 in CRC cell lines, which resulted in a clear radio-enhancement in colony forming assays (Figure 4H). Moreover, we used the RAD51 inhibitor RI-1 to pharmacologically target the protein function in CRC cell lines and organoids. Similar to the RNAi-mediated knockdown, RI-1 sensitized both tumor models to irradiation (Figures 4I and 4J). In summary, these results indicate that MEKi-induced loss of RAD51 is a central mechanism explaining its effect as a radiosensitizer.

CellPress
OPEN ACCESS

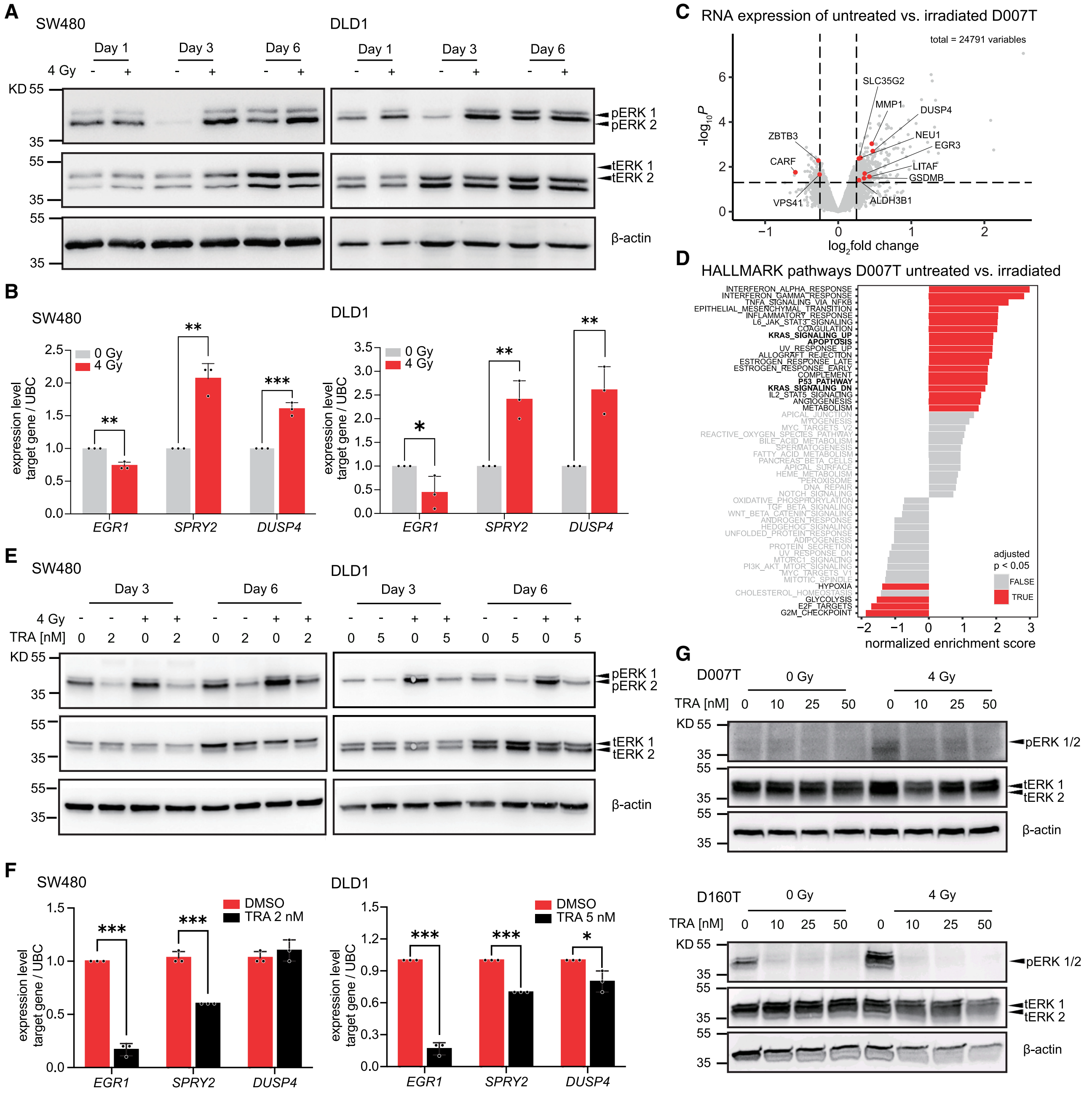

Figure 3. Radiation induces activation of RAS-MAPK signaling

(A) Phosphorylation of ERK1/2 in CRC lines at different time points after irradiation.

(B) Expression of RAS-MAPK pathway target genes is induced in DLD1 and SW480 cell lines 6 days after irradiation.

(C) RNA expression profiling of rectal cancer organoid line D007T, 96 h after irradiation treatment with 4 Gy. Volcano plot of differentially expressed genes in irradiated vs. non-irradiated organoids. Target genes of the EGFR signaling pathway according to PROGENY[22] are highlighted. RNA expression profiling experiments of D080T and D160T can be found in Figure S7C.

(D) Gene set enrichment analysis of HALLMARK[21] gene sets in irradiated vs. non-irradiated organoids D007. Analysis of D080T and D160T can be found in Figure S7D.

(E) Phosphorylation of ERK1/2 in CRC lines after irradiation is reduced by MEKi trametinib (TRA) treatment.

(F) Transcriptional induction of target genes of the RAS-MAPK pathway after irradiation is suppressed by MEK inhibition in CRC cell lines.

(G) Phosphorylation of ERK1/2 in rectal cancer organoids 2 days after irradiation is reduced by concomitant MEKi treatment.

(A, E, G) Representative images of three independent biological replicates are shown.

(C and D) Data from five independent biological replicates are shown.

(B and F) Data from three independent experiments are presented as mean ± SD. *$p < 0.05$, **$p < 0.01$, ***$p < 0.001$ two-tailed Student's t test.

See also Figure S8.

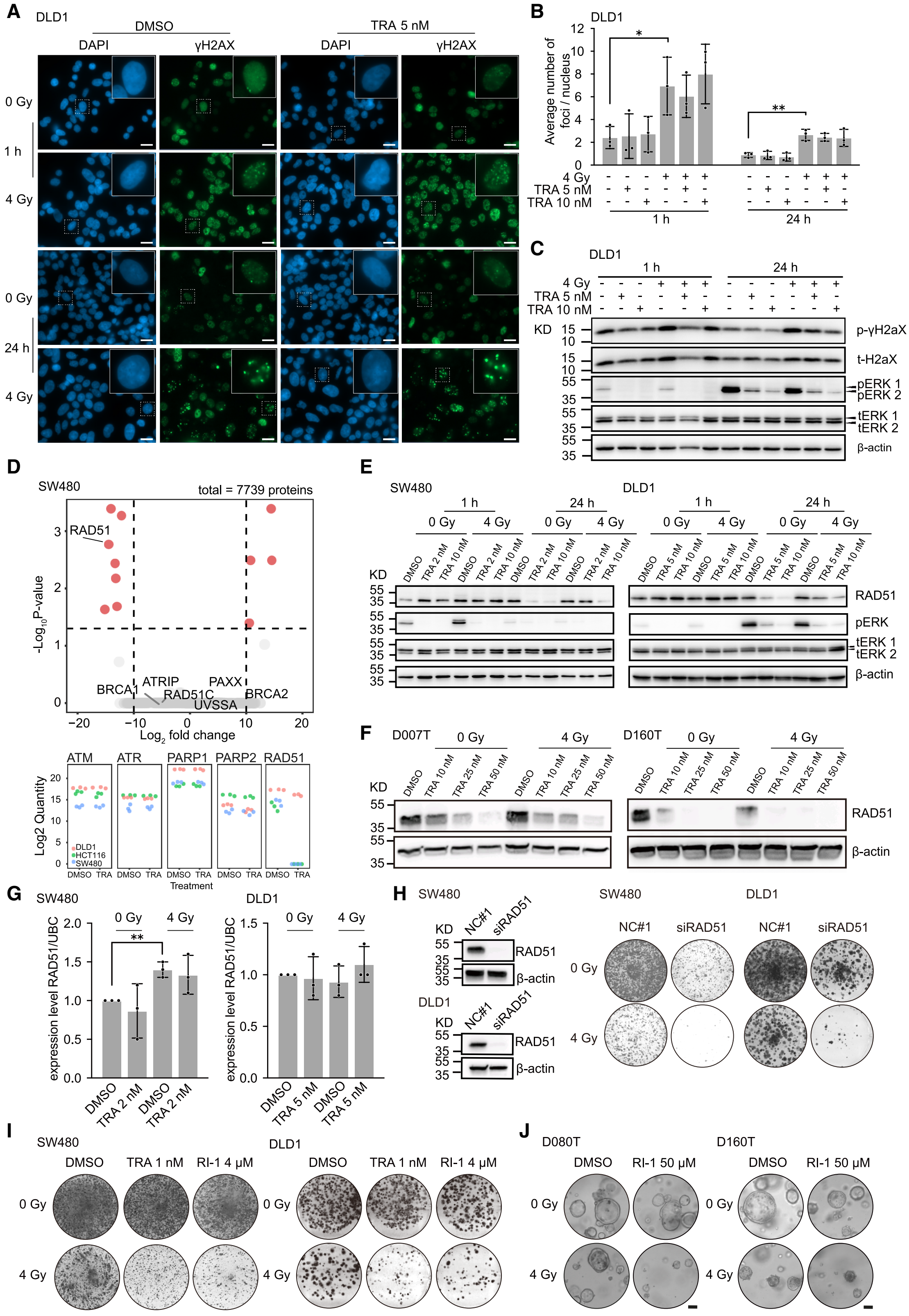

(legend on next page)

CellPress
OPEN ACCESS

MEK and PARP inhibition have synergistic effects on viability in CRC models

Besides MEKi, we also identified inhibitors of additional pathways that strongly enhanced sensitivity to radiation in our primary screen in rectal cancer organoids. We hypothesized that a combination of MEKi with one of these inhibitors could potentiate the antineoplastic effects, particularly when combined with radiation. Specifically, combinations of MEKi with EGFR and phosphatidylinositol 3-kinase (PI3K) inhibitors were previously shown to elicit synergistic antineoplastic effects.[26] Therefore, we performed drug combination experiments in seven rectal cancer organoid lines (five of them previously tested, two new lines as unbiased set, all of them RAS mutated, four *TP53* wild type, and three *TP53* mutated) using high-resolution drug concentration matrices. To this end, MEKi was combined with four drugs representing pathways with strong positive interaction with radiation (PI3K, PARP, EGFR, and CHK1), under irradiated and non-irradiated conditions (Figures 5A and S11A–S11G). Focusing on drug synergy in the absence of radiation first, we determined most relevant combinations, again using the Bliss independence synergy model, and additionally tested further commonly used synergy models (highest single agent, Loewe synergy model, and zero interaction potency [ZIP] model). We found that the combination of MEKi with the PARPi talazoparib and PI3K inhibitor taselisib was consistently synergistic across the tested organoid lines, while EGFR inhibitor dacomitinib and CHK1 inhibitor MK-8776 showed less consistent effects (Figure 5B). Particularly high synergy scores were noted for organoid line D080T. Thus, our assay confirmed previously observed synergies of MEK and PI3K inhibition, as well as EGFR inhibition in CRC models.[26] Since we had previously shown that MEK inhibition interferes with DNA damage response via RAD51, we further focused on PARP inhibition as a combination partner that converged on the DNA repair pathway. We performed in-depth evaluation of combinations of MEKi and PARPi at different concentrations for drug synergism. Using the Bliss synergy model, we found that the drug combination was most synergistic in lower-to-medium concentrations of both PARPi and MEKi, particularly in the range of 0.039–2.5 μM talazoparib and 2.4 nM–0.16 μM trametinib in our organoid assays (Figures 5C–5F and S12). In this concentration range, the increased efficacy of the combination was clearly visible by comparing the observed response to the expected response according to the Bliss synergy model (Figure 5G). We also confirmed these findings using two CRC cell lines, in both short- and long-term viability assays (Figures 5H and 5I). Synergistic antineoplastic effects in short-term proliferation assays were observed in both Bliss and ZIP synergy models (maximum Bliss score: DLD1 18.68, SW480 38.41; maximum ZIP score: DLD1 15.57, SW480 44.47). Together, these results indicate that MEKi and PARPi can synergistically reduce viability in different CRC models at low concentrations, even in the absence of radiation.

Radiation synergizes with MEK-PARP combination therapy

Having shown synergistic viability effects of MEKi with PARPi, we hypothesized that this drug combination would further synergize with radiation in CRC and allow low-dose application of both drugs in this setting, as both compounds target the DNA repair pathway. We therefore analyzed the effect of radiation on combinations of trametinib and talazoparib in cancer organoids using high-density drug concentration matrices, as described earlier. In seven tested organoid lines (including irradiation-resistant lines, lines with stronger irradiation response, and two previously untested lines added as unbiased set), we observed a strong increase in response to combinations of the two drugs when additional radiation was performed (Figures 6A and S13A). Applying a Bliss synergy model, we calculated the expected response to radiation added to combinations of trametinib and talazoparib and found that the observed responses exceeding the calculated Bliss response, particularly at lower doses ranging from 0.6 to 39 nM trametinib combined with 0.039–2.5 μM talazoparib (Figure 6A). This proved the synergy of the two-drug combination with additional radiation. Of note, higher concentrations of the two-drug combination with radiation led to complete killing of almost all organoids, showing the high efficacy of this combination (Figures 6A and S13A). To also prove the synergy of added PARPi to the combination of MEKi with radiation, which we had shown to be synergistic above, we calculated a second Bliss model. This model considered trametinib-radiation and added talazoparib as an independent perturbation (Figures 6B and

Figure 4. MEKi modulates DNA damage response by downregulating DNA repair protein RAD51

(A) Radiation-induced DNA damage as determined by immunofluorescence staining of p-γH2AX. Green, p-γH2AX; blue, DAPI; 63× magnification; scale bars: 20 μm.

(B) Measurement of p-γH2AX foci per nuclei under different treatment conditions.

(C) Immunoblot showing induction of cellular p-γH2AX levels upon radiation.

(D) Global proteome profiling by mass spectrometry of SW480 cells after treatment with 100 nM trametinib vs. DMSO for 24 h, abundance of selected DNA damage response pathway proteins is depicted below.

(E) RAD51 protein expression at different time points after irradiation and MEKi trametinib treatment in CRC cell lines.

(F) RAD51 protein expression 2 days after irradiation and MEKi trametinib treatment in patient-derived rectal cancer organoids.

(G) RNA expression levels of RAD51 in CRC cell lines 24 h after irradiation ± trametinib treatment as determined by qPCR.

(H) Colony-forming assay with CRC cell lines after siRNA-mediated knockdown of RAD51 ± radiation. Staining of cell culture plates was performed 11 days post radiation. Knockdown efficiency of RAD51 after 48 h is shown by western blot (left).

(I) Colony-forming assay in CRC cell lines after treatment with different concentrations of the RAD51 inhibitor RI-1 for 11 days ± radiation. Scans of complete wells of standard 6-well plates are shown (9.6 cm^2 per well) (H and I).

(J) Proliferation of patient-derived rectal cancer organoids after treatment with RI-1 for 2 days and ± radiation, scale bars: 50 μm.

(A, E, F) representative images of three independent biological replicates are shown.

(B and G) Data from three independent experiments are presented as mean ± SD. $^{*}p < 0.05$, $^{**}p < 0.01$, two-tailed t test, *p* values are only shown in case of significant differences.

See also Figures S9 and S10.

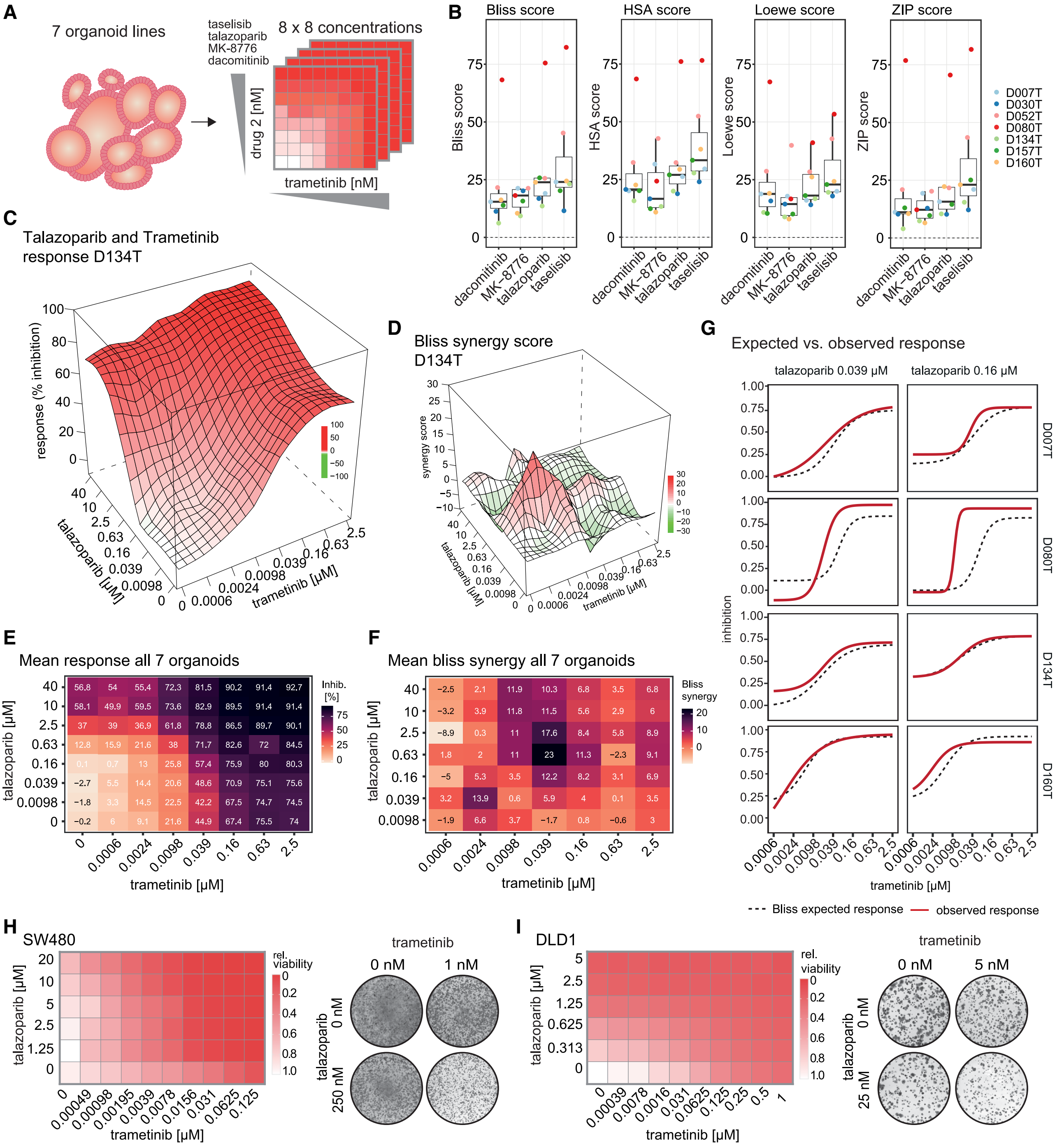

Figure 5. MEK and PARP inhibition have synergistic viability effects in colorectal cancer models

(A) A combination drug screen was performed with MEKi trametinib vs. 4 other top candidates interacting with radiation, derived from the radiosensitization screening experiments shown in Figure 2 (PI3K inhibitor, PARP inhibitor, EGFR inhibitor, and CHK1 inhibitor) in matrices of 7 × 7 concentrations (8 × 8 including DMSO) using 7 organoid lines.

(B) Synergy scores according to Bliss synergy, highest single agent (HSA), Loewe, and zero interaction potency (ZIP) models for the 4 drugs in combination with trametinib are shown. The overall scores represent the highest score of all dose combinations tested, 2 biological replicates were analyzed for D030T and D157T, 3 replicates were analyzed for D007T, D052T, D134T, and D160, and 4 replicates were analyzed for D080T.

(C) Three-dimensional response (% inhibition) surface of the talazoparib and trametinib combination, exemplified by D134T organoids. The surface contains fitted values.

(D) Bliss synergy surface of the talazoparib and trametinib combination in D134 organoids. The surface contains fitted values.

(legend continued on next page)

6C). The observed combination response showed higher potency than the predicted response according to the Bliss model, proving synergy between PARPi and MEKi-radiation (Figure 6C). We also confirmed the markedly enhanced effect of the two-drug-radiation combination by long-term colony-forming assays and short-term viability assays in CRC cell lines (Figures 6D, S13B, and S13C).

Radiation combined with MEK and PARP inhibition leads to significant tumor growth inhibition *in vivo*

To validate these findings and to evaluate potential systemic adverse effects, we performed drug-drug-radiation combination treatments in two xenograft models of CRC. To this end, we performed subcutaneous engraftment of SW480 and DLD1 cell lines in immunocompromised, athymic BALB/c mice. Upon tumor engraftment and after a tumor volume of approximately 100 mm^3 was reached, mice were assigned to receive either a single dose of irradiation (4 Gy), irradiation plus MEKi or PARPi, or irradiation in combination with MEKi and PARPi. Both MEKi and PARPi were administered via oral gavage from day 1– to 4, including the day of irradiation for a total of 4 days (see scheme in Figure 7A). Mice were sacrificed on day 27. Results of these mouse experiments showed that irradiation plus short-term MEKi could not significantly reduce tumor growth when compared to irradiation alone in DLD1 and SW480 xenografts, and irradiation plus PARPi could reduce tumor growth only in DLD1 xenografts (Figures 7B–7G). When MEKi and PARPi were combined, the antiproliferative effect was stronger than with single combinations and significantly exceeded the efficacy of radiation or single drug-radiation combinations. Notably, this short-term drug-radiation combination treatment was well tolerated as it caused only small differences in weight compared to radiation combined with single drug treatment (Figure 7H).

Together, these findings revealed a synergistic effect of radiation with MEKi and PARPi treatment. Mechanistically, this radiosensitizing effect was mediated by blocking radiation-induced RAS-MAPK signaling, as well as by inference with inhibition of DNA damage response through depletion and inhibition of RAD51 by MEKi and additional targeting of this pathway with PARP inhibition (Figure 7I).

DISCUSSION

Chemoradiation is the main therapy for locally advanced rectal cancers, but until now, strategies that specifically target altered signaling pathways in this tumor entity are not established. Our rectal cancer organoid assays can recapitulate clinical responses to chemoradiation similar to previous reports.[11–13] Building on this, we exploited the predictive value of our cancer organoid translational platform to discover drug combinations to enhance radiation response. We performed large-scale radiation-drug screens using this platform and showed that targeting RAS-MAPK signaling by clinically approved MEKi resulted in enhanced response when combined with radiation therapy. Using different CRC models, we revealed that suppression of radiation-induced RAS-MAPK pathway activation and homology-directed DNA repair via RAD51 are major mechanisms by which MEK inhibition enhances radiotherapy. Finally, combinatorial drug-pair plus radiation experiments revealed that the effect of MEKi and radiation in rectal cancer models could be further increased by additional PARP inhibition, which was confirmed *in vivo* by murine xenograft models. Thus, our study provides strong experimental rationale to combine radiotherapy with two clinically approved targeted therapies as a treatment strategy for rectal cancers.

A modulating effect of the RAS-MAPK pathway on radiosensitivity has been described in other cancer entities.[27] MEKi were reported to boost the effect of radiation in pancreatic,[28] lung,[29] and mammary cancer cell lines.[30] Several underlying mechanisms for the radiosensitizing effect of MEKi have been described, most involving DNA damage repair. These include suppression of homologous recombination genes such as DNA-PKcs in different tumor entities,[28,31] resulting, for instance, in a BRCA-like state in melanoma.[32] According to our data, MEK inhibition does not affect the formation or resolution of DNA DSBs in CRC models, as observed in lung and pancreatic cancer cell lines.[29] Instead, our findings suggest that RAD51, but not other important components of the DNA repair pathway, is downregulated in both CRC cell lines and organoids by MEK inhibition. RAD51 is a major component of the homologous recombination repair system and has been considered as a potential target to enhance radiosensitivity.[33] RAD51 was also shown to be a marker of resistance to PARPi in BRCA-mutated breast cancer,[34] and depletion of RAD51 via RNAi could re-sensitize cancer cells to PARP inhibition.[35] A synergy between PARP and MEKi was observed in pancreatic and ovarian cancer, with mechanistic convergence on the homologous recombination repair pathway.[36] Furthermore, a dual PARP-RAD51 inhibitor was developed[37] and showed antineoplastic effects in the absence of radiation. These studies support our observation that downregulation of RAD51 is a potential mechanism for both the radiosensitizing effect of MEKi and its synergy with PARPi.

(E) Heatmap of response (% inhibition) of trametinib-talazoparib combinations. The mean values of all seven tested organoid lines are shown; values for the individual lines were calculated as means of 2–4 biological replicates, as indicated in (B). Results of individual organoid lines are found in Figure S12.

(F) Heatmap Bliss synergy score of trametinib-talazoparib combinations. The average of bliss synergy scores for each dose combination of all seven tested organoid lines is shown. Results of individual organoid lines are found in Figure S12.

(G) Growth inhibition of 4 representative cancer organoid lines treated with increasing concentrations of trametinib in the presence of talazoparib at 0.039 and 0.16 μM. Expected response according to Bliss synergy model and observed response are shown. The mean values of 3 biological replicates are shown for D007T, D134T, and D160, and of 4 replicates for D080T.

(H and I) Viability after combinatorial inhibition of MEK and PARP in short-term and long-term viability assay in CRC cell lines. SW480 (H) and DLD1 (I) were treated for 4 days with trametinib and talazoparib in a concentration matrix, followed by cell viability measurement. Results were normalized to the DMSO control. Means of 3 biological replicates are presented (left). Long-term colony formation assays showing the combinatorial inhibition of MEK and PARP on colony growth compared to any single reagent treatment in CRC cell lines (right). Representative scans of complete wells of standard 6-well plates are shown (9.6 cm^2 per well). See also Figures S11 and S12.

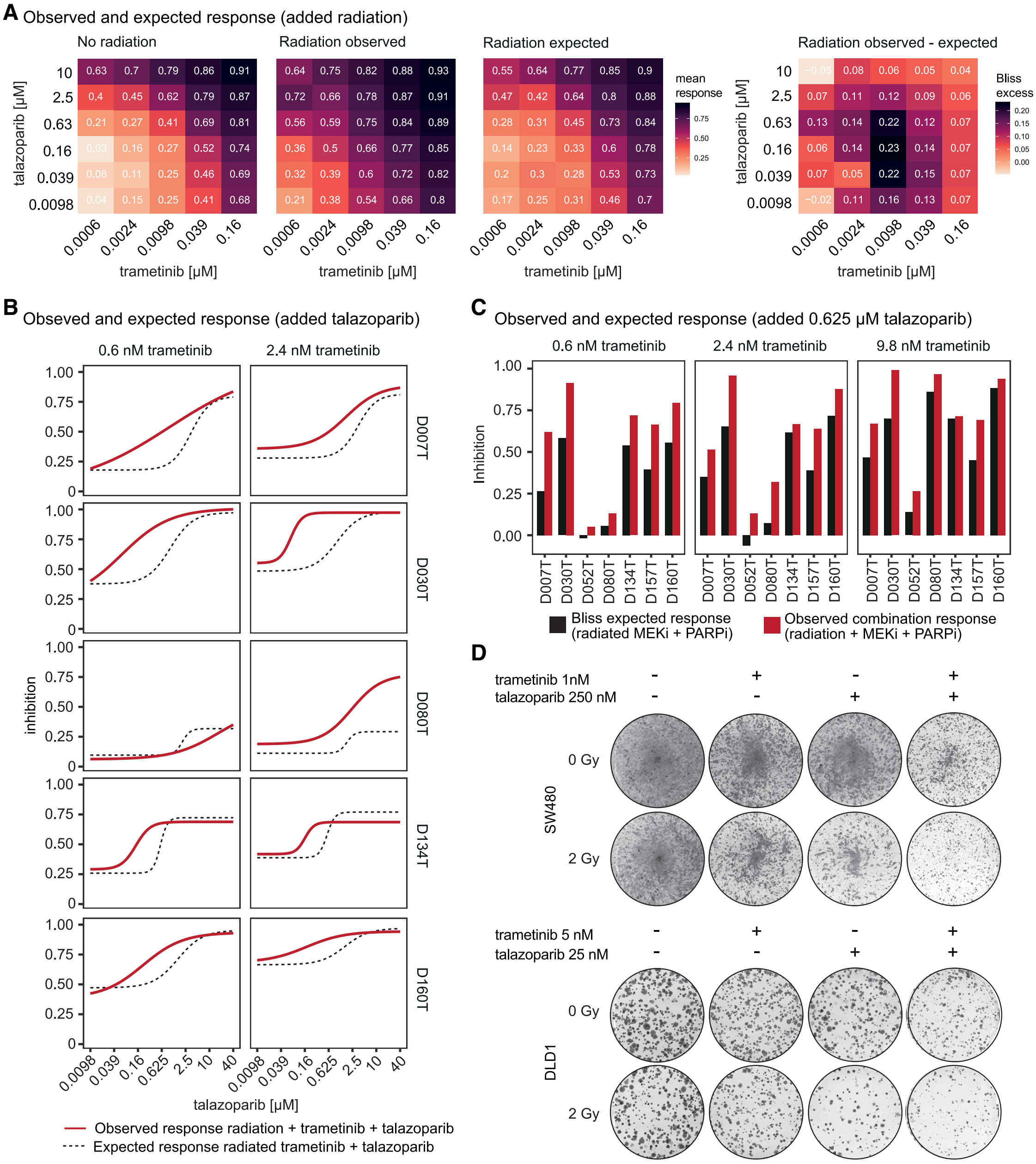

Figure 6. Radiation synergizes with MEK-PARP combination therapy

(A) Response/inhibition matrix derived from talazoparib-trametinib combinations, averaged over all seven tested organoid lines: non-irradiated, irradiated, and Bliss expected response, according to a model of added radiation to fixed combinations of trametinib and talazoparib, as well as Bliss excess (observed response − expected response). Data were normalized to non-irradiated DMSO controls. The lowest 5–6 concentrations tested are shown for each drug.

(B) Dose-inhibition relationships of trametinib-radiation in combination with talazoparib treatments. Bliss expected response was calculated by using trametinib-radiation as one perturbation and adding talazoparib as second perturbation. D007T, D030T, D080T, D134T, and D160T are shown as representative examples of seven tested organoid lines. D007T, D030T, D080T, and D160T were irradiated with 4 Gy, D134T as a more radiation-sensitive line was irradiated with 2 Gy. Mean values of 2 biological replicates are shown for D030T, 3 replicates for D007T, D134T, and D160, and 4 replicates for D080T.

(C) Bar plots of Bliss expected response vs. observed response in organoid lines treated with radiation, 0.625 μM talazoparib and 0.6–9.8 nM trametinib. Bliss expected response was calculated according to the same model as described in (B). Mean values of 2 biological replicates are shown for D030T and D157T, 3 replicates for D007T, D052T, D134T and D160, and 4 replicates for D080T.

(D) Long-term colony formation assays of radiation in combination with MEK inhibition and PARP inhibition on colony growth compared to any single reagent treatment in CRC cell lines. Representative scans of complete wells of standard 6-well plates are shown (9.6 cm^2 per well).

See also Figures S11 and S13.

CellPress
OPEN ACCESS

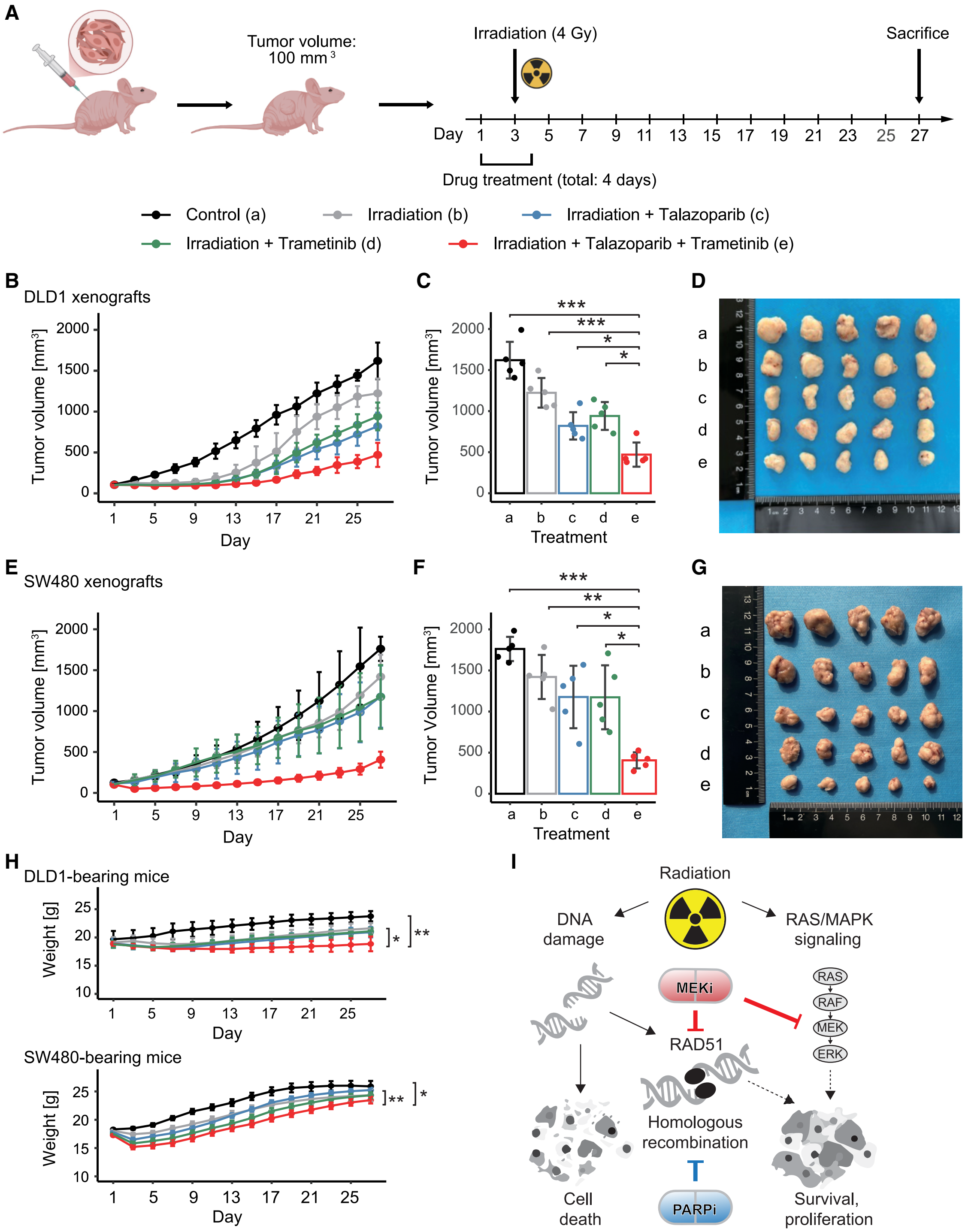

Figure 7. Radiation combined with MEK and PARP inhibition leads to significant tumor growth inhibition *in vivo*

(A) Schematic overview of the *in vivo* experiments.

(B–D) Tumor volume of DLD1 xenografts according to treatment condition over the course of the experiment (B) and at day 27 (C).

(D) Images of the tumors (*n* = 5 per group) after sacrifice of mice, sorted by treatment condition.

(E–G) Tumor volume of SW480 xenografts according to treatment condition over the course of the experiment (E) and at day 27 (F).

(G) Images of the tumors (*n* = 5 per group) after sacrifice of mice, sorted by treatment condition.

(H) Weight of tumor-bearing mice during the experiments, according to experimental conditions. Significant weight differences were observed for the MEKi-PARPi-radiation group versus the untreated and radiation only groups.

(legend continued on next page)

We also observed irradiation-induced activation of RAS-MAPK signaling. RAS-MAPK signaling, as a proliferative signal in cancer cells, can play a role in radio-resistance by promoting tumor growth and overcoming cell-cycle arrest. Accordingly, MEKi abolished radiation-induced activation of RAS-MAPK signaling as an additional mechanism of radiosensitization and a putative way to overcome radio-resistance. Radiation-induced DNA damage can activate RAS-MAPK signaling in untransformed cells such as fibroblasts[38] and keratinocytes,[39] but also in pancreatic or breast cancer cell lines.[40,41] The underlying mechanisms described so far are manifold and include activation of ERK via GADD45β in breast cancer or stimulation of the pathway at the receptor levels via HER1/EGFR.[42–44] ERK1/2 signaling is essential for activation of the G2/M cell cycle checkpoint in response to DNA damage by radiation[45] and also associated with transcriptional upregulation of DNA repair genes, such as ERCC1 and XRCC1.[46] Furthermore, radiation-induced ERK1/2 signaling can activate DNA-PKcs, which plays a critical role in non-homologous end joining-mediated DSB repair.[31] These findings indicate that radiation-induced ERK signaling might represent a specific cellular adaptation to overcome DNA damage, which can be pharmacologically targeted. However, this effect of radiation was not observed in all of our investigated CRC models, indicating that this adaptive response may be subtype specific.

Both MEKi and PARPi have been tested separately in early clinical studies as enhancers and sensitizers of radiation therapy in rectal cancers. A phase 1 trial has been conducted in patients with rectal cancer to determine the maximum tolerated dose of trametinib added to 5-FU-based chemoradiation.[47] A pathological complete response rate of 25% was observed at the maximum tolerated dose, and the treatment was overall well tolerated. Due to the single-arm design, the radiosensitizing effect could only be estimated, but it was higher than the complete response rate of 15% of a matched historical cohort. PARPi can also increase radiosensitivity of CRC cells, particularly in the setting of XRCC deficiency.[48] A phase 1 clinical trial assessed the maximum tolerated dose of the PARPi veliparib combined with neoadjuvant chemoradiation with capecitabine. The combination treatment was well tolerated with no dose-limiting grade III or IV adverse effects and achieved a pathological complete response rate of 28%.[49] Future (clinical) studies may also reveal if a specific subgroup of rectal cancers can particularly benefit from adding MEK-PARP inhibition to radiation therapy. Specifically, whether MEKi-PARPi combinations will potentially increase the radiosensitizing effect of currently used chemotherapeutic agents such as 5-FU remains to be explored by clinical trials.

Limitations of the study

For screening experiments, we chose 2–4 Gy as the screening radiation doses. While varying radiation doses for all lines could potentially have yielded different results in drug-radiation combination screening experiments, preliminary experiments with 4 Gy revealed considerable radiation effects while not being lethal to most cells as a single fraction, thereby allowing a good dynamic range in our experiments leading to the identification of relevant candidate combinations. Additionally, 4 Gy represents a clinically relevant, single-fraction dose in the context of radiation research and is commonly used in preclinical studies.[29,31] Organoid lines with a strong intrinsic sensitivity to radiation were irradiated with a reduced dose of 2 Gy to allow a good dynamic range of drug-radiation combination tests. Our experimental results are based on different preclinical tumor models, including patient-derived organoids and murine xenograft models of common CRC cell lines. A major concern for clinical translation is the potential intestinal toxicity of the MEKi/PARPi-radiation combination. Although we observed only a minor weight loss in our murine xenograft models during combination treatment, the subcutaneous engraftment does not allow us to assess potential adverse effects on more radiation-sensitive tissues such as the intestinal mucosa adjacent to the tumor. Furthermore, resistance mechanisms to RAS-MAPK pathway inhibition could be mediated by the tumor microenvironment, including cancer-associated fibroblasts.[50] These factors are not properly captured by our cancer organoid models, and therefore, the synergistic effect that we observed could be overestimated. Clinical trials will be needed to sufficiently address both questions of toxicity and efficacy of MEKi/PARPi-radiation combinations in rectal cancer. Lastly, the mechanism by which MEKi reduces RAD51 remains not exactly defined. Our experimental results suggest that mRNA levels of RAD51 are not reduced, and no active degradation of RAD51 was observed in the cycloheximide chase assays. Therefore, we suggest that future research should focus on translational regulation of RAD51 by MAPK-RAS signaling by using, for instance, polysome profiling.

In conclusion, we used an organoid platform to discover a strong synergy effect of PARP-MEKi combination with radiotherapy in rectal cancer. We provide molecular explanations for the radiosensitizing effects of MEKi, indicating a convergence of the two inhibitors on the DNA repair pathway. Given that both PARPi and MEKi show promising results in phase 1 neoadjuvant radiation trials with low levels of toxicity, our study advocates combining both agents with radiation in future clinical trials for rectal cancer.

RESOURCE AVAILABILITY

Lead contact

Further information and requests for resources and reagents should be directed to the lead contact, Johannes Betge (j.betge@dkfz.de).

Materials availability

Requests for materials or reagents should be directed to the lead contact.

Data and code availability

- Expression profiling data were deposited in Gene Expression Omnibus (GEO:https://www.ncbi.nlm.nih.gov/geo/ with project number

(B–H) Two-way ANOVA with Tukey's multiple comparisons test was used to test statistical significance. $^{*}p < 0.05$, $^{**}p < 0.01$, $^{***}p < 0.001$.

(I) Putative mechanisms of interaction of MEK-PARP-radiation combination therapy. Radiation leads to DNA damage, which induces the cellular DNA damage repair machinery to enable cancer cell survival. Additionally, the RAS-MAPK pathway is upregulated. MEKi block radiation-induced RAS-MAPK signaling and downregulate RAD51, a core protein of the DNA DSB homologous recombination repair pathway. Addition of PARPi further enhances the effect by targeting DNA damage response via a different target.

GSE294953). Proteomics data were deposited in the PRIDE repository and are available in ProteomeXchange: PXD063024. Next-generation sequencing data of organoids can be made available through the European Genome Phenome Archive (EGA: https://ega-archive.org under the accession number EGAD00001004313) and the German Human Genome Phenome Archive (GHGA: https://data.ghga.de/ under the accession number GHGAS14639338878282). Data access requests for sequence data will be evaluated and transferred upon completion of a data transfer agreement and authorization by the data access committee at the University Medical Center Mannheim and DKFZ under the premise of adhering to EU General Data Protection Regulation.

- No nonstandard code was used to generate, analyze, or plot the data presented in this study. Codes to analyze the data and generate the plots are available from the corresponding authors upon reasonable request.
- Any additional information required to reanalyze the data reported in this work paper is available from the lead contact upon reasonable request.

ACKNOWLEDGMENTS

We thank the NGS Core Facility of the German Cancer Research Center for help with exome sequencing and the Omics-IT Facility of DKFZ for help with sequencing data analysis. We thank the DKFZ Microarray core facility for performing expression profiling experiments. We thank Rosemarie Euler-Lange, Miriam Bierbaum, and Adriana Grbenicek for their help with radiation experiments with organoids and cells. We thank Dr. Junyan Lu for discussions on data analysis. We acknowledge funding from the German Research Foundation grant SFB1324 (R.I., N.V., J.K., E.E., M.B., and T.Z.), the German Research Foundation grant GRK2727, B1.3 (E.B. and M.P.E.), a DKFZ-Hector Cancer Institute seed funding grant (T.Z. and J. Betge), the China Scholarship Council Program (Q.X., X.Y., and L.W.), the DKFZ International PhD Program (P.A.), the Oversea study program of the Guangzhou Elite Project (Z.L.), the German National Academic Foundation (J.E.R.), and the Hector Foundation II (J. Betge).

AUTHOR CONTRIBUTIONS

Conceptualization, Q.X., J.E.R., T.Z., and J. Betge; methodology, Q.X., J.E.R., T. Mulholland, P.A., E.V., M.B., M.P.E., T.Z., and J. Betge; investigation, Q.X., J.E.R., T. Mulholland, Z.L., J. Buchloh, P.A., X.Y., M.L., N.V., O.S., A.K., E.E., L.W., S.B., N.S., D.S., K.T., K.E.B., Y.P., T. Miersch, E.B., K.C., Y.Z., Y.M., C.H., M.R.V., C.B., R.I., J.K., and I.K.; visualization, Q.X., J.E.R., T. Mulholland, T.Z., and J. Betge; funding acquisition, T.Z. and J. Betge; project administration, T.Z. and J. Betge; supervision, T.Z. and J. Betge; writing – original draft, Q.X., J.E.R., T. Mulholland, T.Z., and J. Betge; writing – review and editing, Z.L., J. Buchloh, P.A., X.Y., M.L., N.V., O.S., A.K., K.E.B., E.B., K.C., Y.Z., Y.M., C.H., M.R.V., C.B., R.I., J.K., I.K., M.B., and M.P.E.

DECLARATION OF INTERESTS

The authors declare no competing interests.

STAR★METHODS

Detailed methods are provided in the online version of this paper and include the following:

- KEY RESOURCES TABLE
- EXPERIMENTAL MODEL AND STUDY PARTICIPANT DETAILS
  - Colorectal cancer patient-derived organoids
  - Human cell lines and cell culture
  - Mice and xenograft tumor models
- METHOD DETAILS
  - Patient-derived cancer organoid culture
  - DNA sequencing of cancer organoids
  - RNA microarrays of cancer organoids
  - Drug-radiation screens in cancer organoids
  - Drug libraries
  - Drug-drug-radiation combinations
  - Drug treatment
  - Radiation treatment
  - Viability readout
  - Radiation treatment of cell lines
  - Cell line viability assay
  - Cell line colony formation assay
  - Organoid proliferation assay
  - γH2AX foci assay
  - Quantitative PCR
  - Immunoblot
  - RNA interference
  - Cycloheximide chase assay
  - Mass spectrometry (MS)
  - Data analysis for mass spectrometry
- QUANTIFICATION AND STATISTICAL ANALYSIS
  - General statistics and reproducibility
  - Quality controls in drug-radiation screens
  - Dose-response curves and area under the curve
  - Doubling time calculation
  - Growth rate inhibition metrics
  - Drug-drug and drug-radiation combination analysis

SUPPLEMENTAL INFORMATION

Supplemental information can be found online at https://doi.org/10.1016/j.xcrm.2025.102284.

Received: August 26, 2024
Revised: April 23, 2025
Accepted: July 11, 2025
Published: August 8, 2025

REFERENCES

1. Siegel, R.L., Miller, K.D., Fuchs, H.E., and Jemal, A. (2021). Cancer Statistics, 2021. CA Cancer J. Clin. *71*, 7–33. https://doi.org/10.3322/caac.21654.

2. Glynne-Jones, R., Wyrwicz, L., Tiret, E., Brown, G., Rödel, C., Cervantes, A., and Arnold, D.; ESMO Guidelines Committee (2017). Rectal cancer: ESMO Clinical Practice Guidelines for diagnosis, treatment and follow-up. Ann. Oncol. *28*, iv22–iv40. https://doi.org/10.1093/annonc/mdx224.

3. Sauer, R., Liersch, T., Merkel, S., Fietkau, R., Hohenberger, W., Hess, C., Becker, H., Raab, H.-R., Villanueva, M.-T., Witzigmann, H., et al. (2012). Preoperative Versus Postoperative Chemoradiotherapy for Locally Advanced Rectal Cancer: Results of the German CAO/ARO/AIO-94 Randomized Phase III Trial After a Median Follow-Up of 11 Years. J. Clin. Oncol. *30*, 1926–1933. https://doi.org/10.1200/jco.2011.40.1836.

4. Bahadoer, R.R., Dijkstra, E.A., van Etten, B., Marijnen, C.A.M., Putter, H., Kranenbarg, E.M.-K., Roodvoets, A.G.H., Nagtegaal, I.D., Beets-Tan, R.G.H., Blomqvist, L.K., et al. (2021). Short-course radiotherapy followed by chemotherapy before total mesorectal excision (TME) versus preoperative chemoradiotherapy, TME, and optional adjuvant chemotherapy in locally advanced rectal cancer (RAPIDO): a randomised, open-label, phase 3 trial. Lancet Oncol. *22*, 29–42. https://doi.org/10.1016/s1470-2045(20)30555-6.

5. Rödel, C., Fokas, E., and Gani, C. (2017). Complete response after chemoradiotherapy for rectal cancer: what is the reasonable approach? Innov. Surg. Sci. *3*, 47–53. https://doi.org/10.1515/iss-2017-0041.

6. Dijkstra, E.A., Hospers, G.A.P., Kranenbarg, E.M.-K., Fleer, J., Roodvoets, A.G.H., Bahadoer, R.R., Guren, M.G., Tjalma, J.J.J., Putter, H., Crolla, R.M.P.H., et al. (2022). Quality of life and late toxicity after short-course radiotherapy followed by chemotherapy or chemoradiotherapy for locally advanced rectal cancer – The RAPIDO trial. Radiother. Oncol. *171*, 69–76. https://doi.org/10.1016/j.radonc.2022.04.013.

7. Wu, Y., Song, Y., Wang, R., and Wang, T. (2023). Molecular mechanisms of tumor resistance to radiotherapy. Mol. Cancer *22*, 96. https://doi.org/10.1186/s12943-023-01801-2.

8. Roeder, F., Meldolesi, E., Gerum, S., Valentini, V., and Rödel, C. (2020). Recent advances in (chemo-)radiation therapy for rectal cancer: a comprehensive review. Radiat. Oncol. *15*, 262. https://doi.org/10.1186/s13014-020-01695-0.

9. Chatila, W.K., Kim, J.K., Walch, H., Marco, M.R., Chen, C.-T., Wu, F., Omer, D.M., Khalil, D.N., Ganesh, K., Qu, X., et al. (2022). Genomic and transcriptomic determinants of response to neoadjuvant therapy in rectal cancer. Nat. Med. *28*, 1646–1655. https://doi.org/10.1038/s41591-022-01930-z.

10. Betge, J., and Jackstadt, R. (2023). From organoids to bedside: Advances in modeling, decoding and targeting of colorectal cancer. Int. J. Cancer *152*, 1304–1313. https://doi.org/10.1002/ijc.34297.

11. Ganesh, K., Wu, C., O'Rourke, K.P., Szeglin, B.C., Zheng, Y., Sauvé, C.-E. G., Adileh, M., Wasserman, I., Marco, M.R., Kim, A.S., et al. (2019). A rectal cancer organoid platform to study individual responses to chemoradiation. Nat. Med. *25*, 1607–1614. https://doi.org/10.1038/s41591-019-0584-2.

12. Park, M., Kwon, J., Kong, J., Moon, S.M., Cho, S., Yang, K.Y., Jang, W.I., Kim, M.S., Kim, Y., and Shin, U.S. (2021). A Patient-Derived Organoid-Based Radiosensitivity Model for the Prediction of Radiation Responses in Patients with Rectal Cancer. Cancers *13*, 3760. https://doi.org/10.3390/cancers13153760.

13. Yao, Y., Xu, X., Yang, L., Zhu, J., Wan, J., Shen, L., Xia, F., Fu, G., Deng, Y., Pan, M., et al. (2020). Patient-Derived Organoids Predict Chemoradiation Responses of Locally Advanced Rectal Cancer. Cell Stem Cell *26*, 17–26. https://doi.org/10.1016/j.stem.2019.10.010.

14. Muzny, D.M., Bainbridge, M.N., Chang, K., Dinh, H.H., Drummond, J.A., Fowler, G., Kovar, C.L., Lewis, L.R., Morgan, M.B., Newsham, I.F., et al. (2012). Comprehensive molecular characterization of human colon and rectal cancer. Nature *487*, 330–337. https://doi.org/10.1038/nature11252.

15. Betge, J., Rindtorff, N., Sauer, J., Rauscher, B., Dingert, C., Gaitantzi, H., Herweck, F., Srour-Mhanna, K., Miersch, T., Valentini, E., et al. (2022). The drug-induced phenotypic landscape of colorectal cancer organoids. Nat. Commun. *13*, 3135. https://doi.org/10.1038/s41467-022-30722-9.

16. Lawrence, T.S., Blackstock, A.W., and McGinn, C. (2003). The mechanism of action of radiosensitization of conventional chemotherapeutic agents. Semin. Radiat. Oncol. *13*, 13–21. https://doi.org/10.1053/srao.2003.50002.

17. Chalmers, A., Johnston, P., Woodcock, M., Joiner, M., and Marples, B. (2004). PARP-1, PARP-2, and the cellular response to low doses of ionizing radiation. Int. J. Radiat. Oncol. Biol. Phys. *58*, 410–419. https://doi.org/10.1016/j.ijrobp.2003.09.053.

18. Bashi, A.C., Coker, E.A., Bulusu, K.C., Jaaks, P., Crafter, C., Lightfoot, H., Milo, M., McCarten, K., Jenkins, D.F., van der Meer, D., et al. (2024). Large-scale Pan-cancer Cell Line Screening Identifies Actionable and Effective Drug Combinations. Cancer Discov. *14*, 846–865. https://doi.org/10.1158/2159-8290.cd-23-0388.

19. Cao, C., Shinohara, E.T., Subhawong, T.K., Geng, L., Kim, K.W., Albert, J. M., Hallahan, D.E., and Lu, B. (2006). Radiosensitization of lung cancer by nutlin, an inhibitor of murine double minute 2. Mol. Cancer Ther. *5*, 411–417. https://doi.org/10.1158/1535-7163.mct-05-0356.

20. Dent, P., Yacoub, A., Fisher, P.B., Hagan, M.P., and Grant, S. (2003). MAPK pathways in radiation responses. Oncogene *22*, 5885–5896. https://doi.org/10.1038/sj.onc.1206701.

21. Liberzon, A., Birger, C., Thorvaldsdóttir, H., Ghandi, M., Mesirov, J.P., and Tamayo, P. (2015). The Molecular Signatures Database Hallmark Gene Set Collection. Cell Syst. *1*, 417–425. https://doi.org/10.1016/j.cels.2015.12.004.

22. Schubert, M., Klinger, B., Klünemann, M., Sieber, A., Uhlitz, F., Sauer, S., Garnett, M.J., Blüthgen, N., and Saez-Rodriguez, J. (2018). Perturbation-response genes reveal signaling footprints in cancer gene expression. Nat. Commun. *9*, 20. https://doi.org/10.1038/s41467-017-02391-6.

23. Scully, R., Panday, A., Elango, R., and Willis, N.A. (2019). DNA double-strand break repair-pathway choice in somatic mammalian cells. Nat. Rev. Mol. Cell Biol. *20*, 698–714. https://doi.org/10.1038/s41580-019-0152-0.

24. Morgan, M.A., and Lawrence, T.S. (2015). Molecular Pathways: Overcoming Radiation Resistance by Targeting DNA Damage Response Pathways. Clin. Cancer Res. *21*, 2898–2904. https://doi.org/10.1158/1078-0432.ccr-13-3229.

25. Grundy, M.K., Buckanovich, R.J., and Bernstein, K.A. (2020). Regulation and pharmacological targeting of RAD51 in cancer. NAR Cancer *2*, zcaa024. https://doi.org/10.1093/narcan/zcaa024.

26. Britten, C.D. (2013). PI3K and MEK inhibitor combinations: examining the evidence in selected tumor types. Cancer Chemother. Pharmacol. *71*, 1395–1409. https://doi.org/10.1007/s00280-013-2121-1.

27. Munshi, A., and Ramesh, R. (2013). Mitogen-Activated Protein Kinases and Their Role in Radiation Response. Genes Cancer *4*, 401–408. https://doi.org/10.1177/1947601913485414.

28. Estrada-Bernal, A., Chatterjee, M., Haque, S.J., Yang, L., Morgan, M.A., Kotian, S., Morrell, D., Chakravarti, A., and Williams, T.M. (2015). MEK inhibitor GSK1120212-mediated radiosensitization of pancreatic cancer cells involves inhibition of DNA double-strand break repair pathways. Cell Cycle *14*, 3713–3724. https://doi.org/10.1080/15384101.2015.1104437.

29. Chung, E.J., Brown, A.P., Asano, H., Mandler, M., Burgan, W.E., Carter, D., Camphausen, K., and Citrin, D. (2009). In vitro and In vivo Radiosensitization with AZD6244 (ARRY-142886), an Inhibitor of Mitogen-activated Protein Kinase/Extracellular Signal-regulated Kinase 1/2 Kinase. Clin. Cancer Res. *15*, 3050–3057. https://doi.org/10.1158/1078-0432.ccr-08-2954.

30. Carter, S., Auer, K.L., Reardon, D.B., Birrer, M., Fisher, P.B., Valerie, K., Schmidt-Ullrich, R., Mikkelsen, R., and Dent, P. (1998). Inhibition of the mitogen activated protein (MAP) kinase cascade potentiates cell killing by low dose ionizing radiation in A431 human squamous carcinoma cells. Oncogene *16*, 2787–2796. https://doi.org/10.1038/sj.onc.1201802.

31. Marampon, F., Gravina, G.L., Di Rocco, A., Bonfili, P., Di Staso, M., Fardella, C., Polidoro, L., Ciccarelli, C., Festuccia, C., Popov, V.M., et al. (2011). MEK/ERK Inhibitor U0126 Increases the Radiosensitivity of Rhabdomyosarcoma Cells In vitro and In vivo by Downregulating Growth and DNA Repair Signals. Mol. Cancer Ther. *10*, 159–168. https://doi.org/10.1158/1535-7163.mct-10-0631.

32. Maertens, O., Kuzmickas, R., Manchester, H.E., Emerson, C.E., Gavin, A. G., Guild, C.J., Wong, T.C., De Raedt, T., Bowman-Colin, C., Hatchi, E., et al. (2019). MAPK pathway suppression unmasks latent DNA repair defects and confers a chemical synthetic vulnerability in BRAF, NRAS, and NF1 mutant melanomas. Cancer Discov. *9*, 526–545, CD-18-0879. https://doi.org/10.1158/2159-8290.cd-18-0879.

33. Ward, A., Khanna, K.K., and Wiegmans, A.P. (2015). Targeting homologous recombination, new pre-clinical and clinical therapeutic combinations inhibiting RAD51. Cancer Treat Rev. *41*, 35–45. https://doi.org/10.1016/j.ctrv.2014.10.006.

34. Cruz, C., Castroviejo-Bermejo, M., Gutiérrez-Enríquez, S., Llop-Guevara, A., Ibrahim, Y.H., Gris-Oliver, A., Bonache, S., Morancho, B., Bruna, A., Rueda, O.M., et al. (2018). RAD51 foci as a functional biomarker of homologous recombination repair and PARP inhibitor resistance in germline BRCA-mutated breast cancer. Ann. Oncol. *29*, 1203–1210. https://doi.org/10.1093/annonc/mdy099.

35. Liu, Y., Burness, M.L., Martin-Trevino, R., Guy, J., Bai, S., Harouaka, R., Brooks, M.D., Shang, L., Fox, A., Luther, T.K., et al. (2017). RAD51 Mediates Resistance of Cancer Stem Cells to PARP Inhibition in

Triple-Negative Breast Cancer. Clin. Cancer Res. *23*, 514–522. https://doi.org/10.1158/1078-0432.ccr-15-1348.

36. Sun, C., Fang, Y., Yin, J., Chen, J., Ju, Z., Zhang, D., Chen, X., Vellano, C. P., Jeong, K.J., Ng, P.K.-S., et al. (2017). Rational combination therapy with PARP and MEK inhibitors capitalizes on therapeutic liabilities in RAS mutant cancers. Sci. Transl. Med. *9*, eaal5148. https://doi.org/10.1126/scitranslmed.aal5148.
37. Malka, M.M., Eberle, J., Niedermayer, K., Zlotos, D.P., and Wiesmüller, L. (2021). Dual PARP and RAD51 Inhibitory Drug Conjugates Show Synergistic and Selective Effects on Breast Cancer Cells. Biomolecules *11*, 981. https://doi.org/10.3390/biom11070981.
38. Tang, D., Wu, D., Hirao, A., Lahti, J.M., Liu, L., Mazza, B., Kidd, V.J., Mak, T.W., and Ingram, A.J. (2002). ERK Activation Mediates Cell Cycle Arrest and Apoptosis after DNA Damage Independently of p53. J. Biol. Chem. *277*, 12710–12717. https://doi.org/10.1074/jbc.m111598200.
39. Ahmed, K.M., Nantajit, D., Fan, M., Murley, J.S., Grdina, D.J., and Li, J.J. (2009). Coactivation of ATM/ERK/NF-κB in the low-dose radiation-induced radioadaptive response in human skin keratinocytes. Free Radic. Biol. Med. *46*, 1543–1550. https://doi.org/10.1016/j.freeradbiomed.2009.03.012.
40. Williams, T.M., Flecha, A.R., Keller, P., Ram, A., Karnak, D., Galbán, S., Galbán, C.J., Ross, B.D., Lawrence, T.S., Rehemtulla, A., and Sebolt-Leopold, J. (2012). Cotargeting MAPK and PI3K Signaling with Concurrent Radiotherapy as a Strategy for the Treatment of Pancreatic Cancer. Mol. Cancer Ther. *11*, 1193–1202. https://doi.org/10.1158/1535-7163.mct-12-0098.
41. Wang, T., Hu, Y.-C., Dong, S., Fan, M., Tamae, D., Ozeki, M., Gao, Q., Gius, D., and Li, J.J. (2005). Co-activation of ERK, NF-κB, and GADD45β in Response to Ionizing Radiation. J. Biol. Chem. *280*, 12593–12601. https://doi.org/10.1074/jbc.m410982200.
42. Hein, A.L., Ouellette, M.M., and Yan, Y. (2014). Radiation-induced signaling pathways that promote cancer cell survival. Int. J. Oncol. *45*, 1813–1819, (Review). https://doi.org/10.3892/ijo.2014.2614.
43. Dittmann, K., Mayer, C., Fehrenbacher, B., Schaller, M., Raju, U., Milas, L., Chen, D.J., Kehlbach, R., and Rodemann, H.P. (2005). Radiation-induced Epidermal Growth Factor Receptor Nuclear Import Is Linked to Activation of DNA-dependent Protein Kinase. J. Biol. Chem. *280*, 31182–31189. https://doi.org/10.1074/jbc.m506591200.
44. Dent, P., Reardon, D.B., Park, J.S., Bowers, G., Logsdon, C., Valerie, K., and Schmidt-Ullrich, R. (1999). Radiation-induced Release of Transforming Growth Factor α Activates the Epidermal Growth Factor Receptor and Mitogen-activated Protein Kinase Pathway in Carcinoma Cells, Leading to Increased Proliferation and Protection from Radiation-induced Cell Death. Mol. Biol. Cell *10*, 2493–2506. https://doi.org/10.1091/mbc.10.8.2493.
45. Yan, Y., Black, C.P., and Cowan, K.H. (2007). Irradiation-induced G2/M checkpoint response requires ERK1/2 activation. Oncogene *26*, 4689–4698. https://doi.org/10.1038/sj.onc.1210268.
46. Yacoub, A., McKinstry, R., Hinman, D., Chung, T., Dent, P., and Hagan, M.P. (2003). Epidermal Growth Factor and Ionizing Radiation Up-regulate the DNA Repair Genes XRCC1 and ERCC1 in DU145 and LNCaP Prostate Carcinoma through MAPK Signaling. Radiat. Res. *159*, 439–452. https://doi.org/10.1667/0033-7587(2003)159[0439:egfair]2.0.co;2.
47. Wu, C., Williams, T.M., Robb, R., Webb, A., Wei, L., Chen, W., Mikhail, S., Ciombor, K.K., Cardin, D.B., Timmers, C., et al. (2020). Phase I Trial of Trametinib with Neoadjuvant Chemoradiation in Patients with Locally Advanced Rectal Cancer. Clin. Cancer Res. *26*, 3117–3125. https://doi.org/10.1158/1078-0432.ccr-19-4193.
48. Qin, C., Ji, Z., Zhai, E., Xu, K., Zhang, Y., Li, Q., Jing, H., Wang, X., and Song, X. (2022). PARP inhibitor olaparib enhances the efficacy of radiotherapy on XRCC2-deficient colorectal cancer cells. Cell Death Dis. *13*, 505. https://doi.org/10.1038/s41419-022-04967-7.
49. Czito, B.G., Deming, D.A., Jameson, G.S., Mulcahy, M.F., Vaghefi, H., Dudley, M.W., Holen, K.D., DeLuca, A., Mittapalli, R.K., Munasinghe, W., et al. (2017). Safety and tolerability of veliparib combined with capecitabine plus radiotherapy in patients with locally advanced rectal cancer: a phase 1b study. Lancet Gastroenterol. Hepatol. *2*, 418–426. https://doi.org/10.1016/s2468-1253(17)30012-2.
50. Nicolas, A.M., Pesic, M., Engel, E., Ziegler, P.K., Diefenhardt, M., Kennel, K.B., Buettner, F., Conche, C., Petrocelli, V., Elwakeel, E., et al. (2022). Inflammatory fibroblasts mediate resistance to neoadjuvant therapy in rectal cancer. Cancer Cell *40*, 168–184. https://doi.org/10.1016/j.ccell.2022.01.004.
51. Patel, U.B., Taylor, F., Blomqvist, L., George, C., Evans, H., Tekkis, P., Quirke, P., Sebag-Montefiore, D., Moran, B., Heald, R., et al. (2011). Magnetic Resonance Imaging–Detected Tumor Response for Locally Advanced Rectal Cancer Predicts Survival Outcomes: MERCURY Experience. J. Clin. Oncol. *29*, 3753–3760. https://doi.org/10.1200/jco.2011.34.9068.
52. Reisinger, E., Genthner, L., Kerssemakers, J., Kensche, P., Borufka, S., Jugold, A., Kling, A., Prinz, M., Scholz, I., Zipprich, G., et al. (2017). OTP: An automatized system for managing and processing NGS data. J. Biotechnol. *261*, 53–62. https://doi.org/10.1016/j.jbiotec.2017.08.006.
53. Jones, D.T.W., Hutter, B., Jäger, N., Korshunov, A., Kool, M., Warnatz, H.-J., Zichner, T., Lambert, S.R., Ryzhova, M., Quang, D.A.K., et al. (2013). Recurrent somatic alterations of FGFR1 and NTRK2 in pilocytic astrocytoma. Nat. Genet. *45*, 927–932. https://doi.org/10.1038/ng.2682.
54. Wang, K., Li, M., and Hakonarson, H. (2010). ANNOVAR: functional annotation of genetic variants from high-throughput sequencing data. Nucleic Acids Res. *38*, e164. https://doi.org/10.1093/nar/gkq603.
55. Irizarry, R.A., Hobbs, B., Collin, F., Beazer-Barclay, Y.D., Antonellis, K. J., Scherf, U., and Speed, T.P. (2003). Exploration, normalization, and summaries of high density oligonucleotide array probe level data. Biostatistics *4*, 249–264. https://doi.org/10.1093/biostatistics/4.2.249.
56. Gautier, L., Cope, L., Bolstad, B.M., and Irizarry, R.A. (2004). affy—analysis of Affymetrix GeneChip data at the probe level. Bioinformatics *20*, 307–315. https://doi.org/10.1093/bioinformatics/btg405.
57. Ritchie, M.E., Phipson, B., Wu, D., Hu, Y., Law, C.W., Shi, W., and Smyth, G.K. (2015). limma powers differential expression analyses for RNA-sequencing and microarray studies. Nucleic Acids Res. *43*, e47. https://doi.org/10.1093/nar/gkv007.
58. Korotkevich, G., Sukhov, V., Budin, N., Shpak, B., Artyomov, M.N., and Sergushichev, A. (2021). Fast gene set enrichment analysis. bioRxiv, 060012. https://doi.org/10.1101/060012.
59. Veldwijk, M.R., Seibold, P., Botma, A., Helmbold, I., Sperk, E., Giordano, F.A., Gürth, N., Kirchner, A., Behrens, S., Wenz, F., et al. (2019). Association of CD4+ Radiation-Induced Lymphocyte Apoptosis with Fibrosis and Telangiectasia after Radiotherapy in 272 Breast Cancer Patients with >10-Year Follow-up. Clin. Cancer Res. *25*, 562–572. https://doi.org/10.1158/1078-0432.ccr-18-0777.
60. Callister, S.J., Barry, R.C., Adkins, J.N., Johnson, E.T., Qian, W.J., Webb-Robertson, B.-J.M., Smith, R.D., and Lipton, M.S. (2006). Normalization Approaches for Removing Systematic Biases Associated with Mass Spectrometry and Label-Free Proteomics. J. Proteome Res. *5*, 277–286. https://doi.org/10.1021/pr050300l.
61. Zhan, T., Ambrosi, G., Wandmacher, A.M., Rauscher, B., Betge, J., Rindtorff, N., Häussler, R.S., Hinsenkamp, I., Bamberg, L., Hessling, B., et al. (2019). MEK inhibitors activate Wnt signalling and induce stem cell plasticity in colorectal cancer. Nat. Commun. *10*, 2197. https://doi.org/10.1038/s41467-019-09898-0.
62. Bamberg, L.V., Heigwer, F., Wandmacher, A.M., Singh, A., Betge, J., Rindtorff, N., Werner, J., Josten, J., Skabkina, O.V., Hinsenkamp, I., et al. (2022). Targeting euchromatic histone lysine methyltransferases

sensitizes colorectal cancer to histone deacetylase inhibitors. Int. J. Cancer *151*, 1586–1601. https://doi.org/10.1002/ijc.34155.

63. Zhang, J.-H., Chung, T., and Oldenburg, K.R. (1999). A Simple Statistical Parameter for Use in Evaluation and Validation of High Throughput Screening Assays. J. Biomol. Screen *4*, 67–73. https://doi.org/10.1177/108705719900400206.

64. Hafner, M., Niepel, M., Chung, M., and Sorger, P.K. (2016). Growth rate inhibition metrics correct for confounders in measuring sensitivity to cancer drugs. Nat. Methods *13*, 521–527. https://doi.org/10.1038/nmeth.3853.

65. Zheng, S., Wang, W., Aldahdooh, J., Malyutina, A., Shadbahr, T., Tanoli, Z., Pessia, A., and Tang, J. (2022). SynergyFinder Plus: Toward Better Interpretation and Annotation of Drug Combination Screening Datasets. Genom. Proteom. Bioinform. *20*, 587–596. https://doi.org/10.1016/j.gpb.2022.01.004.

STAR★METHODS

KEY RESOURCES TABLE

REAGENT or RESOURCE	SOURCE	IDENTIFIER
Antibodies		
Rabbit anti-p44/42 MAPK (Erk1/2)	Cell Signaling Technology	Cat# 9102; RRID: AB_330744
Rabbit anti-phospho-p44/42 MAPK (Erk1/2, Thr202/Tyr204)	Cell Signaling Technology	Cat# 4370; RRID: AB_2315112
Rabbit anti-Rad51 (D4B10)	Cell Signaling Technology	Cat# 8875; RRID: AB_2721109
Rabbit anti-Phospho-Histone H2A.X (Ser139)	Cell Signaling Technology	Cat# 2577; RRID: AB_2118010
Mouse anti-β-actin (C4) HRP	Santa Cruz Biotechnologies	Cat# sc-47778 HRP; RRID: AB_626632
Goat anti-rabbit IgG, HRP-linked	Cell Signaling Technology	Cat# 7074; RRID: AB_2099233
Horse anti-mouse IgG, HRP-linked	Cell Signaling Technology	Cat# 7076; RRID: AB_330924
Mouse anti-H2A.X (phospho SER139)	Abcam	Cat# ab26350; RRID: AB_470861
Goat anti-Mouse IgG Highly Cross-Adsorbed Secondary Antibody, Alexa Fluor™ Plus 488	Thermo Fisher Scientific	Cat# A32723; RRID: AB_2633275
Biological samples		
CRC PDO line D004T	Betge et al.[15]	N/A
CRC PDO line D007T	Betge et al.[15]	N/A
CRC PDO line D027T	Betge et al.[15]	N/A
CRC PDO line D030T	Betge et al.[15]	N/A
CRC PDO line D046T	Betge et al.[15]	N/A
CRC PDO line D052T	Betge et al.[15]	N/A
CRC PDO line D073T	This paper	N/A
CRC PDO line D080T	This paper	N/A
CRC PDO line D082T	This paper	N/A
CRC PDO line D086T	This paper	N/A
CRC PDO line D104T	This paper	N/A
CRC PDO line D114T	This paper	N/A
CRC PDO line D134T	This paper	N/A
CRC PDO line D147T	This paper	N/A
CRC PDO line D157T	This paper	N/A
CRC PDO line D160T	This paper	N/A
Chemicals, peptides, and recombinant proteins		
Liberase™ Termolysin High (TH)	Roche	Cat# LIBTH-Ro
Matrigel®	Corning	Cat# 356231
Cultrex® Basement Membrane Extract (BME)	bio-techne	Cat# 3432-005-01
Cultrex® Reduced Growth Factor BME, Type 2	bio-techne	Cat# 3533-005-02
Advanced DMEM/F12	Thermo Fisher Scientific	Cat# 12634010
Gibco™ Penicillin/Streptomycin (Pen/Strep)	Thermo Fisher Scientific	Cat# 15140-122
Gibco™ GlutaMAX™	Thermo Fisher Scientific	Cat# 35050061
Gibco™ HEPES (1M)	Thermo Fisher Scientific	Cat# 15630080
PreproTech® Recombinant Human Noggin	Thermo Fisher Scientific	Cat# 120-10c
B27™ Supplement (50x), serum free	Thermo Fisher Scientific	Cat# 17504044
N-acetyl-L-cysteine	Sigma Aldrich	Cat# A9165

(*Continued on next page*)

Continued

REAGENT or RESOURCE	SOURCE	IDENTIFIER
Nicotinamide	Sigma Aldrich	Cat# N0636
Gastrin I Human	Sigma Aldrich	Cat# G9145
PreproTech® recombinant Human EGF	Thermo Fisher Scientific	Cat# AF-100-15
PreproTech® recombinant Human FGF-10	Thermo Fisher Scientific	Cat# 100-26
A83-01	Biocat	Cat# T3031
Prostaglandin E2 (PGE2)	Santa Cruz Biotechnology	Cat# sc-201225
Primocin®	InvivoGen	Cat# ant-pm
Y-27632	Selleck Chemicals	Cat# S1049
Gibco™ RPMI 1640 Medium	Thermo Fisher Scientific	Cat# 11875085
Gibco™ McCoy's 5A (Modified) Medium	Thermo Fisher Scientific	Cat# 16600082
Gibco™ Fetal Bovine Serum (FBS)	Thermo Fisher Scientific	Cat# A5670701
Gibco™ L-Glutamine	Thermo Fisher Scientific	Cat# 25030081
Gibco™ TrypLE Express Enzyme	Thermo Fisher Scientific	Cat# 12604013
Kinase Inhibitor drug library	Selleck Chemicals	See Table S2
Clinical anticancer drug library	Selleck Chemicals	See Table S3
Trametinib	Selleck Chemicals	Cat# S2673
Talazoparib	Selleck Chemicals	Cat# S7048
MK-8776	Selleck Chemicals	Cat# S2735
Taselisib	Selleck Chemicals	Cat# S7103
Dacomitinib	Selleck Chemicals	Cat# S2727
Dimethylsulfoxid (DMSO)	Sigma Aldrich	Cat# D8418
Bortezomib	Selleck Chemicals	Cat# S1013
Nutlin-3a	Selleck Chemicals	Cat# S8059
Gibco™ phosphate buffered saline (PBS)	Thermo Fisher Scientific	Cat# 10010023
Crystal violet solution	Sigma Aldrich	Cat# V5265
VECTASHIELD® Antifade Mounting Medium with DAPI	Vector Laboratories	Cat# H-1200-10
RIPA lysis and extraction buffer	Thermo Fisher Scientific	Cat# 89900
cOmplete™ Protease inhibitor	Roche/Merck	Cat# 11697498001
Phosphatase inhibitor cocktail 1	Sigma Aldrich	Cat# P2850
Phosphatase inhibitor cocktail 2	Sigma Aldrich	Cat# P5726
SuperSignal™ West Pico PLUS Chemiluminescent Substrate	Thermo Fisher Scientific	Cat# 34579
Lipofectamine™ RNAiMAX	Thermo Fisher Scientific	Cat# 13778075
GDC-0994	Selleck Chemicals	Cat# S7554
BI-3406	Selleck Chemicals	Cat# S8916
RI-1	Selleck Chemicals	Cat# S8077
MG132	Selleck Chemicals	Cat# S2619
Trypsin (sequencing grade)	Promega	Cat# V5111
**Critical commercial assays**		
DNeasy blood and tissue kit	Qiagen	Cat# 69504
RNeasy Kit	Qiagen	Cat# 74104
Affymetrix Human Genome U133 Plus 2.0 Assay	Affymetrix	N/A
CellTiter-Glo® Luminescent Cell Viability Assay	Promega	Cat# G7572
peqGOLD Total RNA Isolation Kit	VWR Chemicals	Cat# 13-6834-02
Verso cDNA synthesis Kit	Thermo Fisher Scientific	Cat# AB1453A
Pierce™ BSA protein assay kits	Thermo Fisher Scientific	Cat# 23225
4-15% precast Mini-PROTEAN® TGX™	Bio-Rad Laboratories	Cat# 4561081

(*Continued on next page*)

Continued

REAGENT or RESOURCE	SOURCE	IDENTIFIER
Amersham^{TM} Protran® Nitrocellulose Membrane	Sigma Aldrich	Cat# GE10600002
Deposited data		
Expression Profiling	This paper	Gene Expression Omnibus: GSE294953
Next generation sequencing data of organoids	This paper and Betge et al.[15]	European Genome Phenome Archive: EGAD00001004313; German Human Genome Phenome Archive: GHGAS14639338878282
Proteomics	This paper	ProteomeXchange: PXD063024
Experimental models: Cell lines		
HCT116	American Type Culture Collection (ATCC)	CCL-247
DLD1	ATCC	CCL-221
SW480	ATCC	CCL-228
Experimental models: Organisms/strains		
Mouse: BALB/c athymic nude mice	Silaike Jingda Laboratory Animal Co Ltd., Hunan, China	*N*-0007
Oligonucleotides		
Primers for qPCR	This paper, see Table S4	N/A
Dharmacon^{TM} siGENOME^{TM} RAD51 siRNA SMARTPool	Horizon	Cat# M-003530-04-0005
Dharmacon^{TM} siGENOME^{TM} siGENOME Non-Targeting siRNA Control Pool #2	Horizon	Cat# D-001206-14-05
Software and algorithms		
ImageJ	National Institute of Health	https://imagej.net/ij/
SpectronautTM v18	Biognosys	https://biognosys.com/
GraphPad Prism v8.0	GraphPad software	https://www.graphpad.com/
R 4.4.0	CRAN	https://cran.rstudio.com/
Other		
pluriStrainer, 40 μm pore size	pluriSelect	Cat# 43-50040-51
Multidrop^{TM} Combi Reagent Dispenser	Thermo Fisher Scientific	Cat# 5840330
Biomek NX^{P} Liquid Handling Automation	Beckmann Coulter	N/A
Biomek FX^{P} Liquid Handling Automation	Beckmann Coulter	N/A
MultiRad225 Irradiation System	Precision X-ray	N/A
PlateLoc Thermal Microplate Sealer	Agilent	Cat# G5585BA
Mithras LB 940 multimode plate reader	Berthold Technologies	N/A
Versa HD^{TM} clinical linear accelerator	Elekta Synergy	N/A
Infinite® M200 Microplate Reader	Tecan	N/A
Cellstar®, 6-well plate	Greiner Bio-One	Cat# 657160
Axio Observer Z1/Apotome microscope	Zeiss	N/A
MicroAmp^{TM} 96-well reaction plate	Thermo Fisher Scientific	Cat# N8010560
StepOne Plus Real-Time PCR instrument	Thermo Fisher Scientific	Cat# 4376600
FUSION-SL-Advance imaging system	PeqLab	N/A
BioRuptor Pico sonication device	Diagenode	Cat# B01080010
EASY-nLC 1200 system	Thermo Fisher Scientific	N/A
Q Exactive HF Orbitrap mass spectrometer	Thermo Fisher Scientific	N/A
nanoEase M/Z peptide BEH C18 column	Waters	Cat# 186008794
HotSleeve+ column oven	Analytical Sales and Services	Cat# HSI-25L
Dionex UltiMate 3000 UHPLC system	Thermo Fisher Scientific	N/A
Orbitrap Exploris 480 mass spectrometer	Thermo Fisher Scientific	N/A

(*Continued on next page*)

Continued

REAGENT or RESOURCE	SOURCE	IDENTIFIER
Vanquish *Neo* UHPLC system	Thermo Fisher Scientific	N/A
Orbitrap Astral mass spectrometer	Thermo Fisher Scientific	N/A
Aurora Ultimate 25 × 75 C18 UHPLC column	IonOpticks	Cat# AUR3-25075C18
Column Heater	IonOpticks	Cat# COLHTR01
Heater Controller	IonOpticks	Cat# IOHEATCON1

EXPERIMENTAL MODEL AND STUDY PARTICIPANT DETAILS

Colorectal cancer patient-derived organoids

All patients were recruited at University Hospital Mannheim, Heidelberg University, Mannheim, Germany. We included patients diagnosed with rectal cancer in this study and obtained biopsies from their primary tumors via endoscopy. Additionally, two organoid lines from patients with primary colon cancer were used in mechanistic studies. Exclusion criteria were active HIV, HBV or HCV infections. Clinical data, tumor characteristics and molecular tumor data were pseudonymized and collected in a database. The clinical data of the participants of this paper can be found in Table S1. The research was approved by the Medical Ethics Committee II of the Medical Faculty Mannheim, Heidelberg University (Reference no. 2014-633N-MA and 2016-607N-MA). All patients gave written informed consent before tumor biopsy was performed. Magnetic resonance image-based treatment response was assessed by magnetic resonance imaging tumor regression grade (mrTRG) according to Patel et al.[51] and by analyzing tumor length before and after treatment. MrTRG 1 refers to the absence of any tumor signal and represents complete regression, whereas mrTRG 5 refers to only tumor signal without any fibrosis event and represents no regression. Patients with mrTRG between 1 and 3 were classified as "responders", patients with mrTRG of 4 or 5 as non-responders. MRI images were assessed by one radiologist (MFF) with long-standing experience in MRI assessment, who was blinded to organoid response. Additionally, tumor regression grade after chemoradiation was assessed by pathological assessment according to Dworak regression grading. Grade 4 represents complete response in this system, grade 0 no response. We classified tumors with grades 3 and 4 as responders and tumors with grades between 0 and 2 as non-responders.

Human cell lines and cell culture

HCT116, SW480, and DLD1 cells were obtained from the American Type Culture Collection (ATCC). DLD1 and SW480 cells were cultured in RPMI 1640 medium (Gibco), and HCT116 cells were cultured in McCoy's 5A medium (Gibco). 2D cell culture media were supplemented with 10% fetal bovine serum (FBS, Gibco), 1% L-glutamine (Gibco) and 1% penicillin/streptomycin (Gibco). Absence of mycoplasma was confirmed by regular PCR-based testing.

Mice and xenograft tumor models

All animal procedures were approved by the Central South University Animal Ethics Committee (CSU-2024-0219). Xenograft models were established by subcutaneously injecting DLD1 and SW480 colorectal cancer cells (5×10^6/200 μL) into the hindlimbs of 4-week-old female athymic BALB/c nude mice (Silaike Jingda Laboratory Animal Co Ltd.). Mice were randomized into five groups ($n = 5$) when tumor volumes reached 100 mm^3: A) Untreated control; B) 4 Gy irradiation (Day 3) C) Talazoparib (0.1 mg/kg/day p.o., Days 1–4) + 4 Gy; D) Trametinib (0.5 mg/kg/day p.o., Days 1–4) + 4Gy; E) Triple therapy Trametinib (0.5 mg/kg/day p.o., Days 1–4) + Talazoparib (0.1 mg/kg p.o., Days 1–4) + 4 Gy. Body weight was measured using an electric scale and tumor dimensions were monitored using a Vernier caliper. Tumor volume was calculated using the formula volume (v) = length (a) * width (b) * width (b) * 0.52. Measurements were performed every 48 h. All animals underwent euthanasia on Day 27 for tumor excision.

METHOD DETAILS

Patient-derived cancer organoid culture

Organoid cultures were extracted from tumor biopsies as reported previously.[15] In short, biopsies were washed and digested with Liberase TH (Roche) before embedding into Matrigel (Corning) or BME (Cultrex). Advanced DMEM/F12 (Thermo Fisher Scientific) medium with Pen/Strep (Gibco), Glutamax (Gibco) and HEPES (Gibco) was supplemented with 100 ng/mL Noggin (PeproTech), 1 x B27 (Thermo Fisher Scientific), 1.25 mM n-Acetyl Cysteine (Sigma), 10 mM Nicotinamide (Sigma), 50 ng/mL human EGF (Peprotech), 10 nM Gastrin (Peprotech), 500 nM A83-01 (Biocat), 10 nM Prostaglandin E2 (Santa Cruz Biotechnology), and 100 mg/mL Primocin (Invivogen). 10 μM Y-27632 (Selleck chemicals) were added after thawing and passaging. Organoids were passaged every 7–10 days and medium was refreshed every 2–3 days.

DNA sequencing of cancer organoids

Hot-spot mutations in cancer-related genes were analyzed as previously described by amplicon sequencing in previously published lines,[15] or by exome sequencing using DKFZ-OTP.[52,53] DNA was isolated using the DNeasy blood and tissue kit (Qiagen). Variants were annotated with ANNOVAR[54] and only exonic or splicing mutations classified as "frameshift deletion", "frameshift insertion", "nonsynonymous SNV", "stopgain" or "stoploss", with an allele frequency >0.1 present in COSMIC in hot-spot genes APC, RAS genes, TP53 and PI3CA were considered for further analysis.

RNA microarrays of cancer organoids

Organoid RNA was isolated with the RNeasy kit (Qiagen) following the manufacturer's instructions. Organoids were pelleted by centrifugation and frozen in RLT buffer containing 1% β-mercaptoethanol before RNA isolation. Samples were hybridized on Affymetrix Human Genome U133 plus 2.0 arrays (Affymetrix). Data were analyzed as previously reported.[15] In short, raw microarray data were normalized using the robust multi-array average (RMA) method[55] followed by quantile normalization as implemented in the "affy" R/Bioconductor package.[56] Differential gene expression analyses were performed using a moderated t-test as implemented in the R/Bioconductor package "limma".[57] Gene set enrichment analyses were performed as implemented in the "fgsea" R/Bioconductor package for ranked gene lists.[58]

Drug-radiation screens in cancer organoids

Cell seeding

For cell seeding, organoids were first incubated with TrypLE Express (Gibco) at 37°C until small clusters and single cells were obtained. Chemical separation was supported by mechanical shearing using a 1000 μL pipette and digestion was visually controlled by light microscopy. Organoid fragments were filtered through a 40 μm strainer (pluriSelect) to prevent large organoid clusters and afterward quantified as previously described.[15] Culture medium was supplemented with Y-27632 and growth factor-reduced BME type 2 was added to a concentration of 0.75 mg/mL (seeding medium). For seeding, the required number of organoids was resuspended in seeding medium and 50 μL of organoid suspension were seeded into each well using a multidrop dispenser (Thermo Fisher Scientific), before centrifugation for 10 min at 1000g at room temperature. Additional organoids were seeded in a proliferation plate running in parallel to determine the proliferation during the incubation period of radiation treatment.

Drug libraries

For the drug-radiation combination screen two libraries were used: A kinase inhibitor library with 224 compounds (Table S2) and a clinical library of 140 drugs of which the majority was clinically approved, supplemented with selected inhibitors of interest for enhancing radiation (Table S3). The clinical library contains a comprehensive selection of FDA-approved cancer-targeting small molecule drugs, which can be modeled in our platform. Antibodies, antibody-drug-conjugates as well as drugs mainly targeting the immune system or tumor microenvironment were excluded. The kinase library was used at a maximum concentration of 10 μM and three 10-fold dilution steps to finally screen 4 different drug concentrations. Drugs within the clinical library and their maximum concentrations were selected individually based on literature review of 2D and 3D cell culture assays, as well as own previous experiments. Each compound was screened in five different concentrations after each 5-fold dilutions. Thus, a total of 1596 drug perturbations (considering all drugs and concentrations) were tested in our assays. 5 μM bortezomib was used as positive control, DMSO as negative control. Both libraries were arranged in a random layout using a Biomek NX^P robotic system (Beckman Coulter). All drugs were purchased from Selleck Chemicals.

Drug-drug-radiation combinations

For testing drug-drug-radiation combinations, trametinib was used in combination with talazoparib, MK-8776, taselisib and dacomitinib. Each was used in seven concentrations; each 4-fold diluted. Including DMSO, each trametinib concentration was combined with all other drugs in 8 concentrations in an 8 x 8 combination matrix. DMSO was used as negative, bortezomib in 5 μM as positive control.

Drug treatment

Drug treatment was performed on day 3 after seeding. Medium was aspirated, discarded and 45 μL fresh medium was added, drugs were pre-diluted in medium and 5 μL of diluted drugs were added. All pipetting steps were performed by a Biomek FX^P robotic device (Beckman Coulter). Plates were covered with plastic lids for radiation treatment.

Radiation treatment

Radiation was performed about two hours after drug treatment. Organoids were irradiated using a MultiRAD 225 irradiation system (Precision X-ray) with a voltage of 200 kV, a current of 17.8 mA and a 0.5 mm copper filter. X-ray dose rate was 2.151 Gy/min. The dose rate was regularly controlled and re-calibrated by the department for radiation protection and radiological dosimetry (DKFZ). After radiation, plates were sealed using PlateLoc Thermal Microplate Sealer (Agilent) and incubated for 6 days at 37°C and 5% CO_2.

Viability readout

Viability was measured on day 9 after seeding. Medium was aspirated and discarded before 30 μL undiluted CellTiter-Glo solution (Promega) was added to each well. After 30 min of incubation at room temperature, luminescence was measured by a Mithras reader (Berthold Technologies).

Radiation treatment of cell lines

In vitro irradiation of cell lines was conducted using 6 MV X-rays emitted by a Versa HD clinical linear accelerator (Elekta Synergy) at a dose rate of 6.67 Gy/min with a 40 × 40 cm^2 irradiation field. Cells were irradiated in cell-culture plates at source-surface distance of 100 cm while using 15 mm water-equivalent material for dose build-up and 8 cm for backscatter, as described by Veldwijk et al.[59] The dosimetry was regularly performed by medical physicists from the Department of Radiotherapy, University Medical Center Mannheim.

Cell line viability assay

Cells were seeded at a concentration of 2000 cells per well in 96-well plates. Twenty-four hours post seeding, cells were irradiated or sham irradiated (same experimental procedure without applying radiation). Following radiation, cells were treated with either DMSO or drug. Cellular viability was analyzed 5 to 6 days after drug treatment, depending on individual growth rates of CRC cell lines. Cellular viability was determined using CellTiter-Glo assay (Promega) according to the manufacturer's protocol. Readout was performed using a Infinite M200 microplate reader (Tecan).

Cell line colony formation assay

Cells were seeded at a concentration of 1000–4000 cells per well in six-well plates. Twenty-four hours post seeding, cells were irradiated. Following radiation, cells were treated with either DMSO or different concentrations of small molecule inhibitors and incubated in standard conditions of temperature and humidity for 11 days. After this time, plates were washed with PBS, fixed with a methanol and acetic acid solution and stained with 0.05% crystal violet solution (Sigma Aldrich). Plates were scanned and the complete scans of each well of standard 6-well plates (Greiner Bio-one, 9.6 cm^2 per well) are shown in the figures without cropping, if not otherwise specified.

Organoid proliferation assay

Luminescence of the organoids was measured on day 3 after seeding using CellTiter-Glo assay as described above. Luminescence was compared to untreated controls of the radiation response assay to determine the doubling time of each organoid line between the day of treatment and the day of readout.

γH2AX foci assay

Cells were seeded on coverslips in six-well plates and treated with radiation and/or trametinib. One hour and twenty-four hours post radiation, cells were fixed with 3.7% paraformaldehyde in PBS for 20 min, and then blocked with 0.5% Triton X-100 with 1% BSA in PBS for 1 h. The fixed cells were incubated with anti-γH2AX antibodies (Abcam, ab26350, dilution 1:200) for 1 h at room temperature, and then incubated overnight at 4°C with Alexa Fluor 488-labeled secondary antibody (Thermo Fisher Scientific, A32723, 1:200), and mounted with DAPI (Vector Laboratories). Images were acquired using a Axio Observer Z1/Apotome microscope (Zeiss). The cell numbers in regions of interest were counted manually in DAPI-stained images with the "multi-point" tool in ImageJ. Foci counting were determined in the matched γH2AX-stained images using the "find maxima" tool in ImageJ, with a fixed prominence set and manual correction for all images under the same condition. A total of 100 cells per condition were analyzed to determine the average number of foci per cell.

Quantitative PCR

Total RNA was isolated from cells using the peqGOLD Total RNA Isolation Kit (VWR Chemicals). cDNA was synthesized using the Verso cDNA synthesis kit (Thermo Fisher Scientific) with 1 μg of purified total RNA as input. Quantitative PCR was performed in a MicroAmp 96-well reaction plate (Thermo Fisher Scientific) on a StepOne Plus Real-Time PCR instrument (Thermo Fisher Scientific). UBC was used as housekeeping gene for relative quantification. Primer sequences used for quantitative PCR are listed in Table S4.

Immunoblot

Protein extraction was performed using RIPA lysis and extraction buffer (Thermo Fisher Scientific) supplemented with protease inhibitor tablets (Roche) and phosphatase inhibitor cocktails 1–2 (Sigma Aldrich). Protein concentration was measured by BCA protein assay (Thermo Fisher Scientific). Fifteen to thirty micrograms of lysates were separated on 4–15% precast Mini-PROTEAN TGX gels (Bio Rad) and transferred to nitrocellulose membrane (Amersham). Membranes were detected by chemiluminescence staining protocol with SuperSignal chemiluminescent substrate (Thermo Fisher Scientific). Images were acquired using the FUSION-SL-Advance imaging system (PeqLab). All antibodies used are listed in the key resources table.

RNA interference

SW480 and DLD1 cells were seeded on six-well plates (Greiner) at a density of 1×10^5 cells per well. Twenty-four hours after seeding, cells were transfected with Dharmacon siGenome smartPool RAD51 or siGenome non-targeting control 2 siRNAs (both from Horizon) and Lipofectamine RNAiMAX (Thermo Fisher Scientific) with a final concentration of 5 nM siRNA. For subsequent expression analysis of target genes and proteins, cells were harvested 48 h post transfection. For further treatment of transfected cells, the medium containing siRNAs was removed 48 h after transfection and cells were re-seeded into six-well plates or 96-well plates. Twenty-four hours after cell re-seeding, drug and/or radiation treatment was performed.

Cycloheximide chase assay

Cancer cells (5×10^5 per well) were seeded in 6-well plates. Twenty-four hours post seeding, cells were treated with either DMSO or 100 nM trametinib for 8 h, followed by addition of cycloheximide (100 μg/mL) or DMSO. Cells were harvested at 0, 4, 8, and 12 h after addition of cycloheximide, washed with ice-cold PBS, and lysed in saponin-containing buffer (20 mM Tris-HCl pH 7.4, 130 mM NaCl, 2 mM EDTA, 10 mM β-mercaptoethanol, 0.05% saponin) supplemented with protease and phosphatase inhibitors. Lysates were incubated on ice for 10 min, shaken for 30 min at 4°C, and clarified by centrifugation (20.000 × g, 30 min, 4°C). Protein concentration was determined using the BCA assay (Thermo Fisher Scientific), and equal amounts were analyzed by immunoblot.

Mass spectrometry (MS)

HCT116, DLD1 and SW480 cells were seeded on 6-well plates at a density of 20.000 cells/cm^2. Twenty-four hours after seeding, cells were treated with 100 nM trametinib or DMSO as control for 24 h. Cells were then washed twice with ice-cold PBS and harvested on wet ice using a cell scraper with 200 μL of ice-cold PBS containing protease inhibitors (Roche). The cell suspension was then pelleted by centrifugation at 1000 rpm at 4°C. The supernatant was discarded, and the cells were stored at −80°C until further processing. For MS analysis, cell pellets were lysed in 100 μL of lysis buffer containing 6 M guanidine hydrochloride (GuHCl), 5 mM tris-(2-carboxyethyl)phosphine and 10 mM chloroacetamide, boiled for 10 min at 99°C, briefly cooled down on ice and sonicated using a BioRuptor (Diagenode) set to high intensity with 10 cycles (30 s ON/30 s OFF) at 4°C. After sonication, the lysates were centrifuged at 15.000 g for 10 min at 4°C and the supernatants transferred into new tubes. The volume containing the equivalent of 20 μg of total proteins from each sample was transferred into new tubes and diluted to a final concentration of maximum 2 M GuHCl with 25 mM Tris-HCl buffer pH 8.5. The proteins were then digested by trypsin (Promega) at 1:50 ratio at 37°C overnight. After overnight incubation, the samples were acidified by adding formic acid at 1% final concentration to stop the digestion. Prior to MS analysis, a peptide clean-up procedure was performed for each sample using SP3 method, as described elsewhere.[53]

For the proteomics analysis of HCT116 cells, the quantitative measurements were carried out using an EASY-nLC 1200 system (Thermo Fisher Scientific) coupled to a Q Exactive HF Orbitrap mass spectrometer (Thermo Fisher Scientific). The peptides were separated by reverse-phase liquid chromatography with 0.1% formic acid (solvent A) and 80% acetonitrile supplemented with 0.1% formic acid (solvent B) as mobile phases, using a stepped gradient from 4% to 80% solvent B in 120 min on a nanoEase M/Z peptide BEH C18 column (Waters, 250 mm × 75 μm 1/PK, 130 Å, 1.7 μm) heated to 55°C using a HotSleeve+ column oven (Analytical Sales & Services). The peptides were eluted with a constant flow rate of 300 nL/min.

The Q Exactive HF Orbitrap mass spectrometer was operated in data-independent mode (DIA) with a scan range of 350–1650 m/z, orbitrap resolution 240000 FWHM, 3e6 AGC target and maximum injection time (max. IT) 20 ms for MS1 scan. For MS2 scan the parameters were set as follows: orbitrap resolution 30000 FWHM, AGC target 1e6, max. IT 40 ms and the precursors were analyzed in a sequence of 26 windows of variable width with an overlapping region of 0.5 Da from both sides. The normalized collision energy for the fragmentation of precursor ions was set to 27 and a fixed first mass of 250 m/z was set for the acquisition of the MS/MS spectra.

For the proteomics analysis of DLD1 cells, the quantitative measurements were carried out using Dionex UltiMate 3000 UHPLC system (Thermo Fisher Scientific) coupled to an Exploris 480 Orbitrap mass spectrometer (Thermo Fisher Scientific). The peptides were separated by reverse-phase liquid chromatography with 0.1% formic acid (solvent A) and 100% acetonitrile supplemented with 0.1% formic acid (solvent B) as mobile phases, using a stepped gradient from 4% to 80% solvent B in 60 min on a nanoEasy M/Z peptide BEH C18 column (Waters, 250 mm × 75 μm 1/PK, 130 Å, 1.7 μm) mounted in the integrated column compartment of the UltiMate 3000 system heated to 55°C. The peptides were eluted with a constant flow of 300 nL/min.

The Exploris 480 Orbitrap mass spectrometer was operated in DIA mode with a scan range of 350–1400 m/z, orbitrap resolution 120000, normalized AGC target 300%, maxIT set to Auto mode and the precursors were analyzed in a sequence of 19 windows of variable width. The normalized HCD collision energy for the fragmentation of precursor ions was set to 28.

For the proteomics analysis of SW480 cells, the quantitative measurements were carried out using Vanquish *Neo* UHPLC system (Thermo Fisher Scientific) coupled to an Orbitrap Astral mass spectrometer with installed FAIMS interface (Thermo Fisher Scientific). The peptides were separated by reverse-phase liquid chromatography with 0.1% formic acid (solvent A) and 80% acetonitrile supplemented with 0.1% formic acid (solvent B) as mobile phases, using a stepped gradient from 2% to 80% solvent B in 60 min on an Aurora Ultimate C18 column (IonOpticks, 250 mm × 75 μm ID, 1.7 μm) heated to 50°C using a column heater (IonOpticks) powered by Heat Controller (IonOpticks). The peptides were eluted with a constant flow 300 nL/min.

The Orbitrap Astral mass spectrometer was operated in DIA mode with a scan range of 350–980 m/z, orbitrap resolution 240000, normalized AGC target 800%, and maxIT 18 ms. The precursors were analyzed in a sequence of 80 windows of variable width with an

overlapping region of 0.5 Da from both sides. The normalized collision energy for the fragmentation of precursor ions was set to 27. The FAIMS interface was operated in standard resolution mode with CV fixed to −48V and a carrier gas flow 3.5 L/min.

Data analysis for mass spectrometry

The files containing spectral data were analyzed using Spectronaut (version 18) software using a directDIA workflow against a nonredundant UniProt Human Proteome FASTA database from 30.01.2020 with the identification settings as follows: precursor Q-value cutoff 0.01, precursor posterior error probability (PEP) cutoff 0.2, protein Q-value cutoff (experiment-wise) 0.01, protein Q-value cutoff (run-wise) 0.05, protein PEP cutoff 0.75. For the quantification, the data were normalized based on a retention time-dependent local regression model as described,[60] with precursor filtering based on identified Q-value, and maxLFQ quantification method based on inter-run peptide ratios. The proteins were grouped by protein group ID and peptides were grouped by a stripped peptide sequence of the identified precursors.

QUANTIFICATION AND STATISTICAL ANALYSIS

General statistics and reproducibility

The sample size (n), replication and statistical test used for each experiment are specified in the figure legends and methods for each experiment. Power calculations were not performed to determine the sample size before each experiment. Sample sizes were chosen on the basis of experience with the given experiments.[15,61,62] Two-tailed unpaired Student's t test or Welch's t-test were used to analyze statistical significance between two groups. two-way ANOVA and Tukey's multiple comparison test were used to compare multiple groups. Statistical analyses were performed using R (version 4.4.0) or GraphPad Prism (version 8.0). p values < 0.05 were considered as statistically significant. Statistical significance was indicated with asterisks: *$p < 0.05$, **$p < 0.01$, ***$p < 0.001$. Error bars represent standard deviation (SD) of multiple biological replicates as denoted in the figure legends.

Clinical response evaluation was blinded to organoid radiation response. Data collection and outcome assessment for other experiments were not blinded.

Two rectal cancer organoids established for radiation testing were excluded from analyses and further experiments due to potential cross-contamination. One plate of each D030T, D134T, D052T and D160T from drug-drug-radiation experiments, as well as one replicate of D080T in the drug-radiation-screen with the clinical drug library were excluded from further analysis due to exceeding Z′-factor or CV cut-offs. Six individual wells had to be excluded from analysis of drug-drug and drug-drug-radiation experiments of organoid lines D052T, D080T and D160T due to pipetting errors in one of the master drug plates, including 3 wells containing DMSO controls (A07, A21, C22) and there 3 wells containing drug combinations (Trametinib x Taselisib, B20; Trametinib x Talazoparib B22; Trametinib x Talazoparib, E21)

Quality controls in drug-radiation screens

Pearson correlation between replicates, as well as descriptive statistics of positive and negative controls were calculated. The coefficient of variation (CV) was calculated using the standard deviation of negative controls (sd(−)) and the mean of negative controls (mean(−)) as a measurement of the negative controls' distribution.

$$CV = \frac{sd(-)}{mean(-)}$$

If possible, Z′-Factor was calculated as an additional parameter for the distribution of positive and negative controls in drug-radiation screens. For its calculation the standard deviation of positive (sd(+)) and negative controls (sd(−)) as well as the mean of positive (mean(+)) and negative controls (mean(−)) were used.

$$Z' = 1 - \frac{3\,(sd(+)+sd(-))}{abs(mean(-) - mean(-))}$$

Replicates with a Z′-Factor <0.25 or a CV > 0.25 were excluded from analysis.[18,63]

Dose-response curves and area under the curve

Raw luminescence data were normalized to the mean of the radiation-specific DMSO-controls of the corresponding plate. Relative viability values were plotted against the radiation doses to obtain dose-response curves. To obtain comparable AUC values in drug-radiation assays, the logarithmic breaks were projected on a linear axis with the highest concentration being projected to the value 1 and the lowest concentration to the value 0. The other concentration values were equally distributed between 0 and 1 to obtain uniform breaks. AUC was calculated using trapezoid integration implied in the pracma package in R (https://cran.r-project.org/web/packages/pracma/index.html).

Doubling time calculation

Doubling Time (Td) was calculated during the incubation period after radiation treatment. The starting point was the day of treatment (day 3 = t_1) and the endpoint was the day of readout (day 9 = t_2). Additionally, the luminescence values of treatment day (lum_1) and readout day (lum_2) were used for calculation.

$$Td = (t_2 - t_1) * \frac{ln(2)}{ln(lum_2 / lum_1)}$$

Growth rate inhibition metrics

For growth rate adjusted response analysis of the organoids' response to radiation, the method by Hafner et al.[64] was used, to calculate the growth rate inhibition (GR) for each radiation dose (d). The luminescence at the day of treatment (lum_0) and the luminescence at the day of readout after treatment with the respective dose (lum_d) as well as the luminescence without treatment (lum_{ctrl}) were used for calculation.

$$GR(d) = 2^{\frac{log_2(lum_d / lum_0)}{log_2(lum_{ctrl} / lum_0)}} - 1$$

For the radiation and drug combination screens, viability was calculated by dividing the luminescence of each condition by the mean luminescence of DMSO controls. The drug concentration was plotted on a logarithmic x axis. For AUC calculation based on drug concentration, the x axis was divided by uniform breaks between 0 and 1 as described above, while the radiation dose has a linear format.

Drug-drug and drug-radiation combination analysis

Analysis of drug combinations in different organoid lines were done by calculating Bliss synergy to determine excess over the Bliss model as marker for Synergy, similar to recently published work.[18] For Bliss excess, "the single-agent activities of drug A and drug B must be expressed as a probability between 0 and 1:

$$(0 \leq EA \leq 1 \text{ and } 0 \leq EB \leq 1)$$

The observed effect of the combination is also expressed as a probability:

$$(0 \leq EAB \leq 1)$$

This means that the expected Bliss additive effect can be expressed as:

$$\mathrm{EA + EB(1 - EA) = EA + EB - EAEB}$$

A positive "excess" over the expected Bliss additive effect defines a synergistic response".[18] Expected responses according to the Bliss model were compared to the observed response at each dose to identify synergistic dose regions. For synergy calculation of drug-radiation combinations, the Bliss model was used in a similar way to obtain expected response for each drug dose with radiation combination, each normalized to non-irradiated DMSO controls. To analyze synergy of an additional perturbation to an established combination (i.e., radiation added to MEKi + PARPi and PARPi added to radiation + MEKi), we treated the previously established combinations as one factor and the added perturbation as the second factor in the Bliss formula:

$$EC + EAB(1 - EC) = EC + EAB - ECEAB$$

The drug-drug combinations in high density drug concentration matrices were further analyzed with the SynergyFinder Plus package to test further synergy models and visualize inhibition and synergy scores in surface plots.[65] Growth inhibition [%] as well as its standard error (SE) were estimated by bootstrapping of the included replicates. 2 biological replicates were analyzed for D030T, D157T, 3 replicates were analyzed for D007T, D052T, D134T and D160, and 4 replicates were analyzed for D080T. Four different synergy scores were calculated for each drug combination: The Bliss model, as described above, the highest single agent (HSA), Loewe and Zero interaction potency (ZIP) score as implemented in the SynergyFinder Plus package. Metrics are reported as the second highest value found across the entire dose matrices to enable identification of dose-specific maxima of synergy that may be "canceled out" when considering the average values of the full dose matrix, but avoiding overestimation of outliers as previously reported.[18]

Cell Reports Medicine, Volume *6*

Supplemental information

Combined MEK and PARP inhibition enhances radiation response in rectal cancer

Qiyun Xiao, Julian E. Riedesser, Theresa Mulholland, Zhenchong Li, Jonas Buchloh, Philipp Albrecht, Xinchen Yang, Moying Li, Nachiyappan Venkatachalam, Olga Skabkina, Anna Klupsch, Ella Eichhorn, Li Wang, Sebastian Belle, Nadine Schulte, Daniel Schmitz, Matthias F. Froelich, Kyrhatii Trikhirhisthit, Erica Valentini, Kim E. Boonekamp, Yvonne Petersen, Thilo Miersch, Elke Burgermeister, Carsten Herskind, Marlon R. Veldwijk, Christoph Brochhausen, Robert Ihnatko, Jeroen Krijgsveld, Ina Kurth, Yuxing Zhu, Yanni Ma, Ke Cao, Michael Boutros, Matthias P. Ebert, Tianzuo Zhan, and Johannes Betge

Supplementary Figures

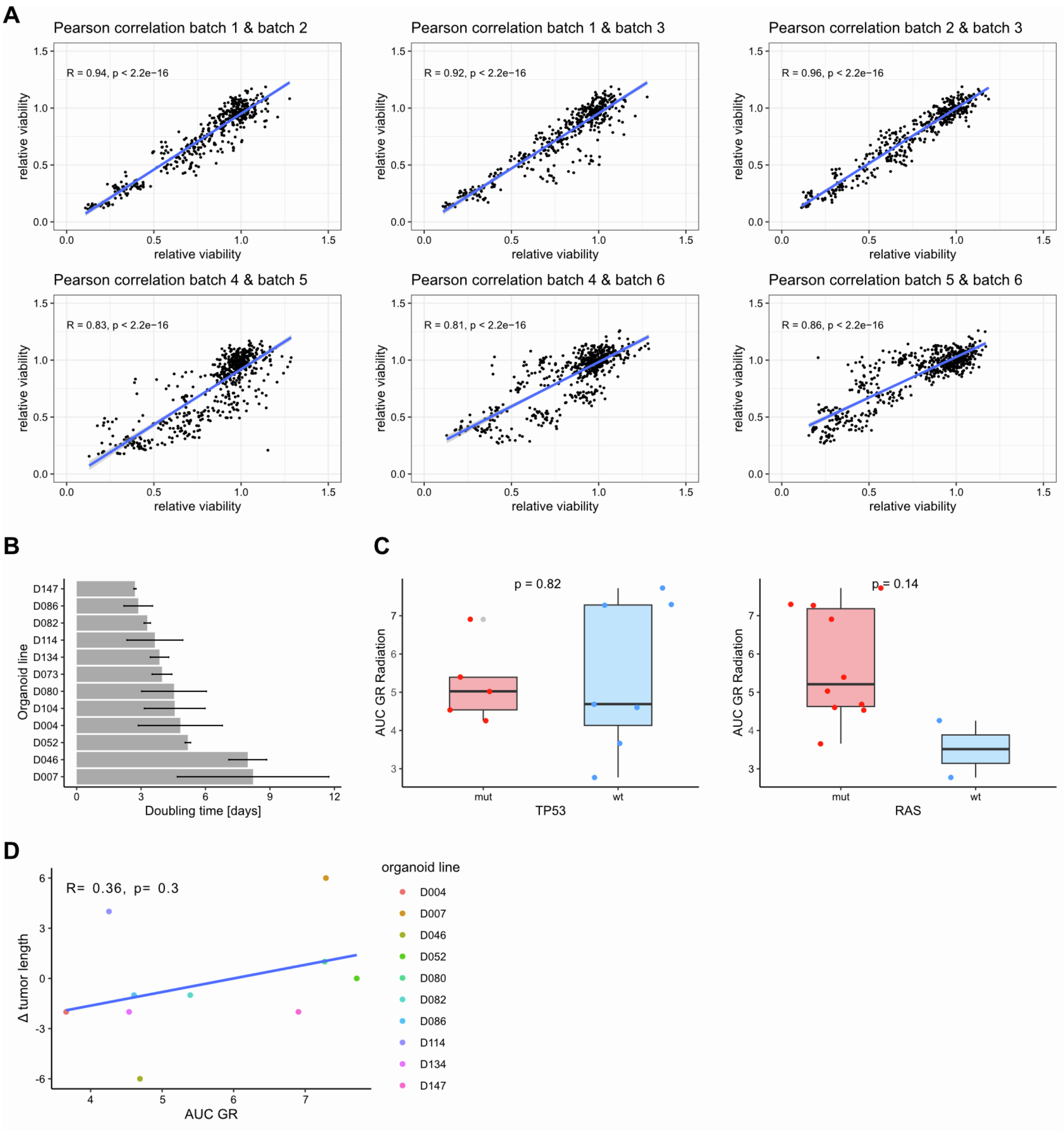

Figure S1: An organoid platform recapitulates clinical responses of rectal cancer to radiation, related to Figure 1. A, Quality controls for the organoid radiation assay, Pearson correlation of biological replicates of the radiation assay are shown in different batches. **B,** Doubling time of organoid lines used in the radiation response assay. Mean ± sd of three biological replicates are presented. **C,** Associations of the response to radiation and TP53 or RAS mutation status, two-tailed t-test. **D,** Pearson correlation of organoid response to radiation (AUC GR) and Δ tumor length before and after radiation therapy, measured in MRI images.

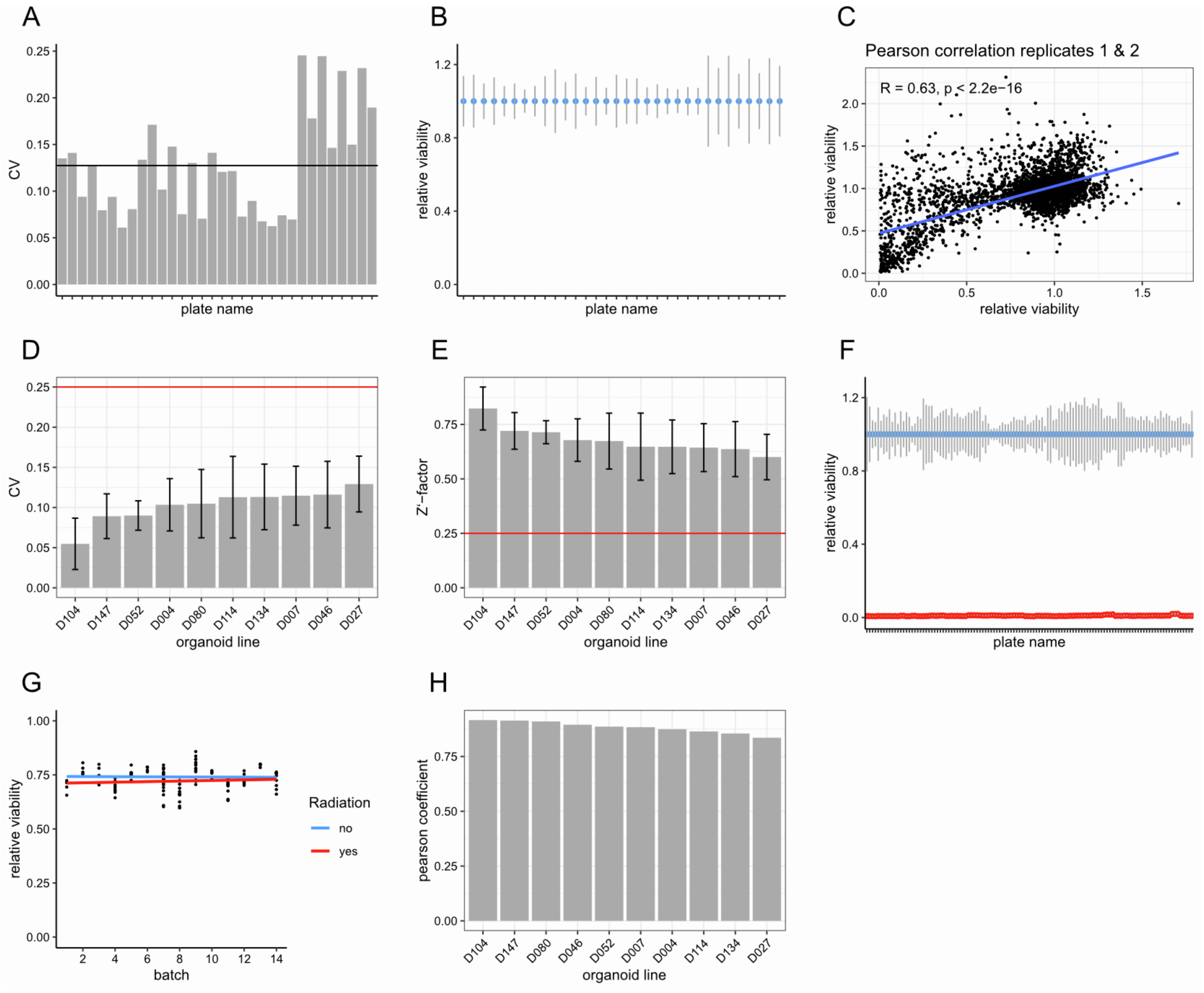

Figure S2: Quality control of drug-radiation screening experiments, related to Figure 2. A-H, Quality controls for the drug-radiation synergism screens. A kinase library of 224 drugs in 4 concentrations was tested in 2 organoid lines. 10 organoid lines were screened with a clinical drug library containing 140 compounds in 5 concentrations. DMSO was used as negative control while high-concentrated bortezomib was the positive control in the clinical library. For each line 2-4 replicates were analyzed. **A,** CV values of the DMSO controls in the kinase library combination screen are plotted for each plate. All CV values were < 2.5. **B,** Normalized luminescence values of DMSO controls for each plate are plotted as mean ± standard deviation. **C,** Pearson correlation of replicates 1 and 2 in the kinase library combination screen. **D,** Mean CV values of the DMSO controls in the clinical library combination screen are plotted as mean ± standard deviation of 2-4 biological replicates. All mean CV values were < 0.25. **E,** Z'-factor was calculated of the raw luminescence values for each plate using the formula described in the Methods section. Mean Z`-factors ± standard deviation of 2-4 biological replicates are shown. F, Distribution of normalized luminescence values of positive and negative controls in the clinical library combination screen. **G,** Mean viability for each plate for different batches. No tendencies were detected. **H,** Average Pearson correlation coefficient of normalized values of 2-4 biological replicates is plotted for each line.

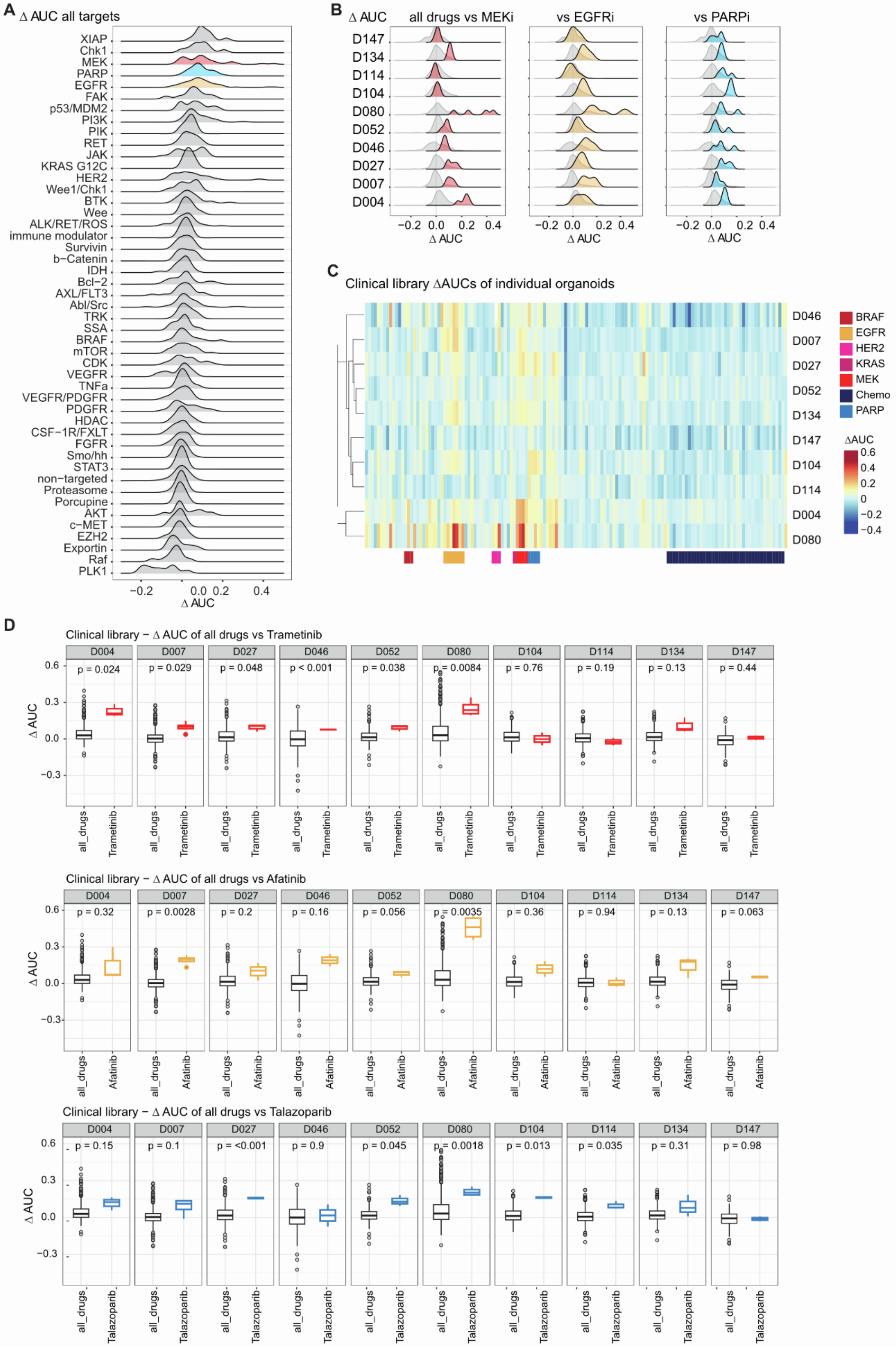
A
Δ AUC all targets
XIAP
Chk1
MEK
PARP
EGFR
FAK
p53/MDM2
PI3K
PIK
RET
JAK
KRAS G12C
HER2
Wee1/Chk1
BTK
Wee
ALK/RET/ROS
immune modulator
Survivin
b−Catenin
IDH
Bcl−2
AXL/FLT3
Abl/Src
TRK
SSA
BRAF
mTOR
CDK
VEGFR
TNFa
VEGFR/PDGFR
PDGFR
HDAC
CSF−1R/FXLT
FGFR
Smo/hh
STAT3
non−targeted
Proteasome
Porcupine
AKT
c−MET
EZH2
Exportin
Raf
PLK1
−0.2
0.0 0.2
0.4
Δ AUC
B
Δ AUC
all drugs vs MEKi
vs EGFRi
vs PARPi
D147
D134
D114
D104
D080
D052
D046
D027
D007
D004
−0.2 0.0 0.2 0.4
Δ AUC
C
Clinical library ΔAUCs of individual organoids
D046
D007
D027
D052
D134
D147
D104
D114
D004
D080
BRAF
EGFR
HER2
KRAS
MEK
Chemo
PARP
ΔAUC
0.6
0.4
0.2
0
−0.2
−0.4
D
Clinical library − Δ AUC of all drugs vs Trametinib
D004 D007 D027 D046 D052 D080 D104 D114 D134 D147
p = 0.024
p = 0.029
p = 0.048
p < 0.001
p = 0.038
p = 0.0084
p = 0.76
p = 0.19
p = 0.13
p = 0.44
Δ AUC
0.6
0.3
0.0
-0.3
all_drugs
Trametinib
Clinical library − Δ AUC of all drugs vs Afatinib
D004 D007 D027 D046 D052 D080 D104 D114 D134 D147
p = 0.32
p = 0.0028
p = 0.2
p = 0.16
p = 0.056
p = 0.0035
p = 0.36
p = 0.94
p = 0.13
p = 0.063
all_drugs
Afatinib
Clinical library − Δ AUC of all drugs vs Talazoparib
D004 D007 D027 D046 D052 D080 D104 D114 D134 D147
p = 0.15
p = 0.1
p = <0.001
p = 0.9
p = 0.045
p = 0.0018
p = 0.013
p = 0.035
p = 0.31
p = 0.98
all_drugs
Talazoparib

Figure S3: Screening for synergistic effects between drugs and radiation, related to Figure 2. A, Distribution of ΔAUCs of inhibitors stratified by mechanism of action of tested drugs in the clinical library. **B,** Distribution of ΔAUCs of MEKi, EGFRi and PARPi in individual organoid lines. **C,** Heatmap of ΔAUCs from the clinical library screen with ten rectal cancer organoids. **D,** Comparisons of ΔAUC of trametinib (MEK inhibitor) with ΔAUCs of all drugs in ten tested organoid lines (top), comparisons of ΔAUC of afatinib (EGFR inhibitor) with ΔAUCs of all drugs in ten tested organoid lines (middle) and comparisons of ΔAUC of talazoparib (PARP inhibitor) with ΔAUCs of all drugs in ten tested organoid lines (top). T-tests were done to assess statistical significance.

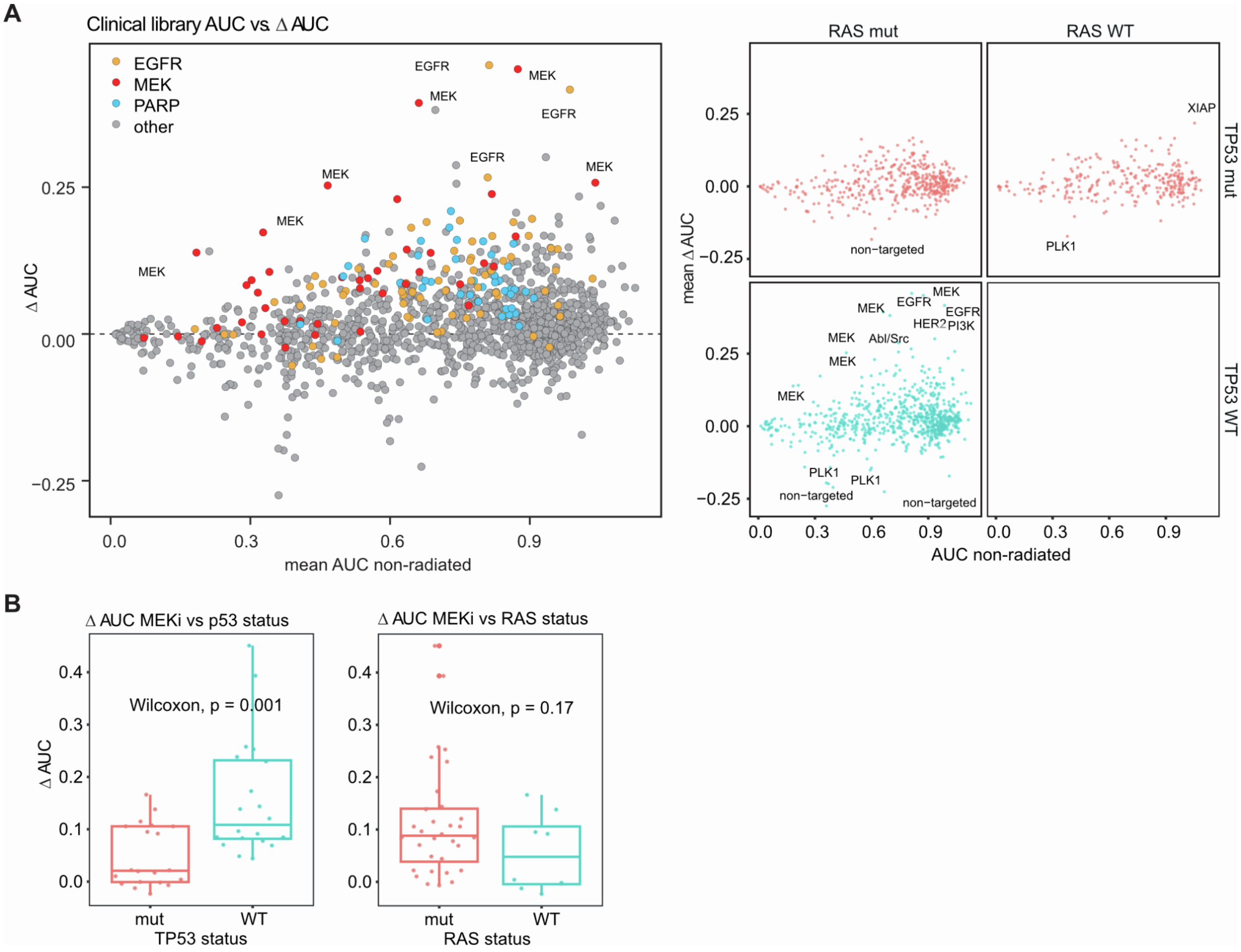

Figure S4: Influence of mutations on drug-radiation-combinations, related to Figure 2. A, Mean ΔAUCs vs. non-radiated AUCs of all lines and drugs tested in the clinical library screen with ten organoid lines according to TP53 and RAS status. **B,** Association of RAS and TP53 mutation status with MEKi ΔAUCs.

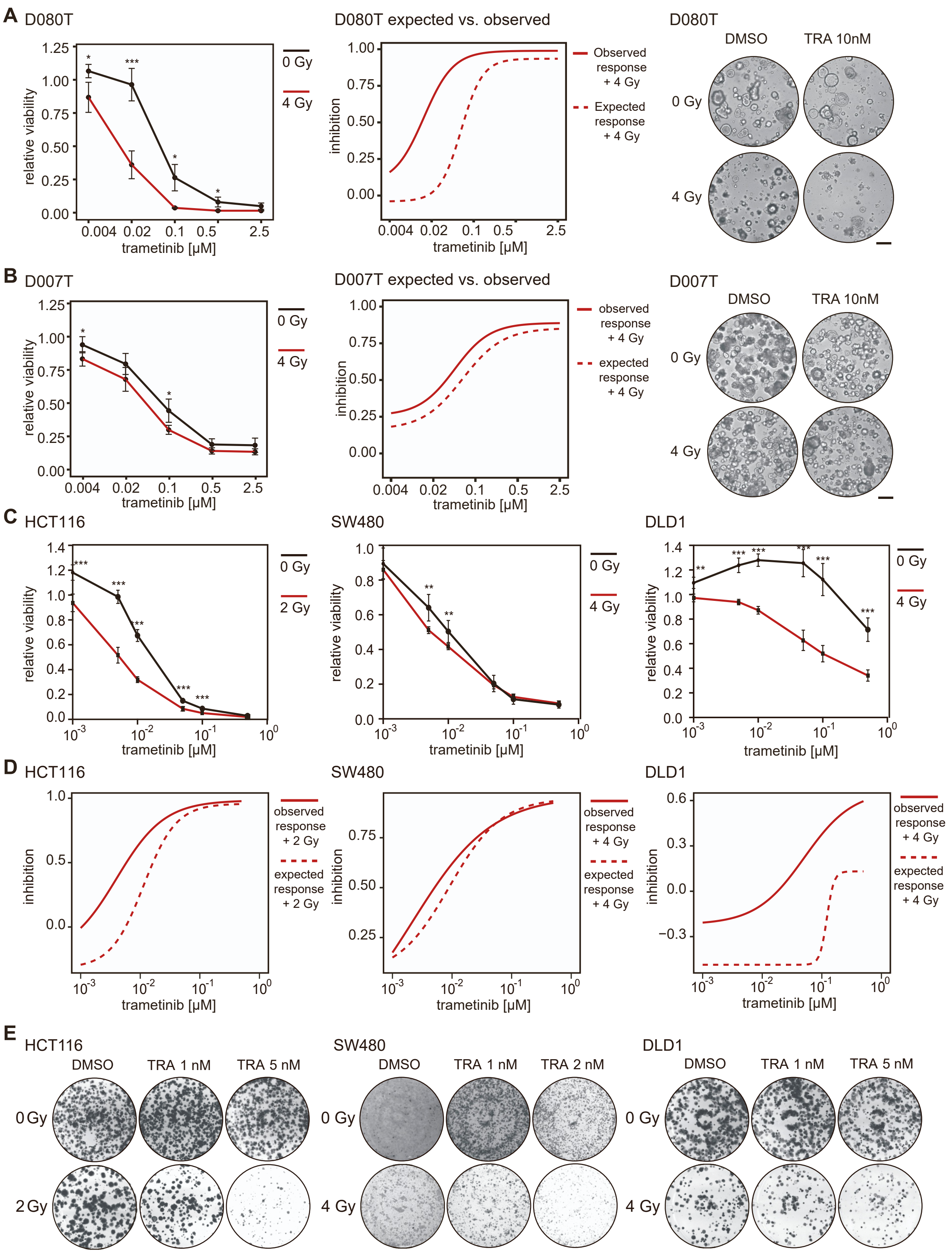

A D080T
D080T expected vs. observed
D080T
DMSO
TRA 10nM
0 Gy
4 Gy
Observed response + 4 Gy
Expected response + 4 Gy
relative viability
inhibition
trametinib [µM]
B D007T
D007T expected vs. observed
D007T
observed response + 4 Gy
expected response + 4 Gy
C HCT116
SW480
DLD1
2 Gy
D HCT116
SW480
DLD1
observed response + 2 Gy
expected response + 2 Gy
E HCT116
SW480
DLD1
TRA 1 nM
TRA 5 nM
TRA 2 nM

Figure S5: MEK inhibition is synergistic with radiation in colorectal cancer cell lines and organoids, related to Figure 2. A, Left: viability assay of organoid line D080T treated with increasing concentrations of MEK inhibitor trametinib with and without radiation, data of the irradiated and non-irradiated plates were normalized to DMSO controls on the same plates in this analysis, respectively, to visualize the additional effect of trametinib in the irradiated condition. Middle: Dose-inhibition relationship of the same data. A Bliss' independence model of trametinib and radiation was used for each concentration to calculate the expected inhibition. For this analysis, all treatments were normalized to non-irradiated DMSO controls. Right: example images of organoids, scale bar: 50 µm. **B,** Left: viability assay of organoid line D007T treated with increasing concentrations of MEK inhibitor trametinib with- and without radiation, data of the irradiated and non-irradiated plates were normalized to DMSO controls on the same plates in this analysis, respectively, to visualize the additional effect of trametinib in irradiated condition. Middle: Dose-inhibition relationship of the same data. A Bliss' independence model of trametinib and radiation was used for each concentration to calculate the expected inhibition. For this analysis, all treatments were normalized to non-irradiated DMSO controls. Right: example images of organoids, scale bar: 50 µm. **C,** Viability assays of CRC cell lines treated with increasing concentrations of MEK inhibitor trametinib with and without radiation. Cell viability was determined after 5-6 days of treatment by CellTiter-Glo. Data of the irradiated and non-irradiated plates were normalized to DMSO controls on the same plates in this analysis, respectively, to visualize the additional effect of trametinib in irradiated condition. **D,** Dose-inhibition relationship of cell lines treated with increasing concentrations of trametinib and radiation. A Bliss' independence model of trametinib and radiation was used for each concentration to calculate the expected inhibition. For this analysis, all treatments were normalized to non-irradiated DMSO controls. **E,** Colony forming assay of CRC cell lines treated with trametinib (TRA) with and without radiation for 10-12 days. Scans of complete wells of standard six-well plates (9.6 cm^2 per well) are shown. A-B, E, representative images of at least three independent biological replicates are shown. A-D, Data from three (cell lines) and four (organoids) biological replicates are presented as mean ± SD. *$p < 0.05$, **$p < 0.01$, ***$p < 0.001$, two-tailed t-test.

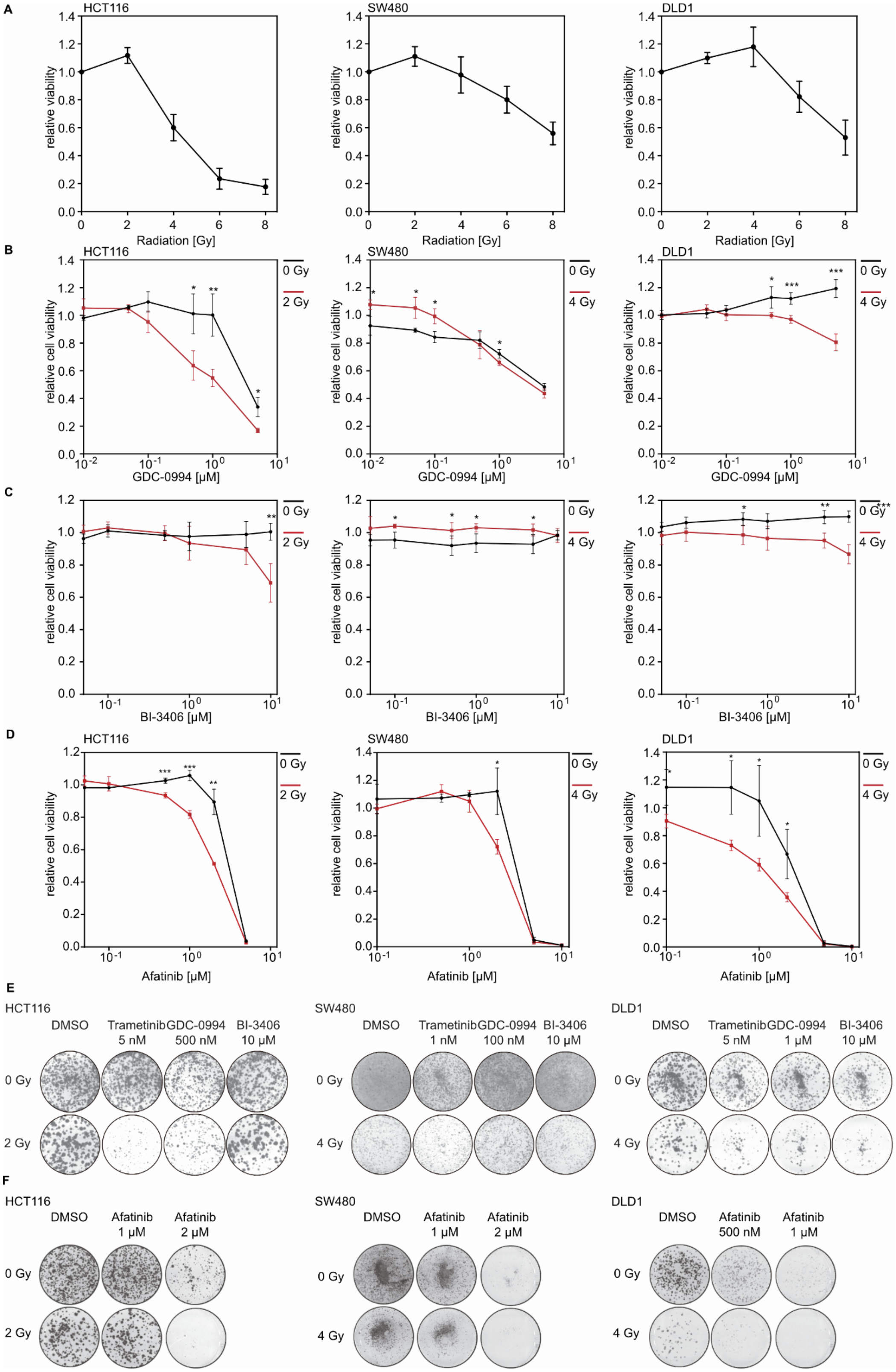
A
HCT116
SW480
DLD1
relative viability
Radiation [Gy]
B
HCT116
SW480
DLD1
relative cell viability
GDC-0994 [µM]
0 Gy
2 Gy
4 Gy
C
relative cell viability
BI-3406 [µM]
0 Gy
2 Gy
4 Gy
D
HCT116
SW480
DLD1
relative cell viability
Afatinib [µM]
0 Gy
2 Gy
4 Gy
E
HCT116
DMSO
Trametinib 5 nM
GDC-0994 500 nM
BI-3406 10 µM
0 Gy
2 Gy
SW480
DMSO
Trametinib 1 nM
GDC-0994 100 nM
BI-3406 10 µM
0 Gy
4 Gy
DLD1
DMSO
Trametinib 5 nM
GDC-0994 1 µM
BI-3406 10 µM
0 Gy
4 Gy
F
HCT116
DMSO
Afatinib 1 µM
Afatinib 2 µM
0 Gy
2 Gy
SW480
DMSO
Afatinib 1 µM
Afatinib 2 µM
0 Gy
4 Gy
DLD1
DMSO
Afatinib 500 nM
Afatinib 1 µM
0 Gy
4 Gy

Figure S6: Effect of KRAS:SOS1, ERK1/2 and EGFR inhibitors on radiosensitivity of CRC cell lines, related to Figure 2. A, Intrinsic radiosensitivity of CRC cell lines. **B-D,** Viability assay of CRC cell lines treated with increasing concentrations of ERK inhibitor GDC-0994 (B), KRAS:SOS1 inhibitor BI-3406 (C) and EGFR inhibitor afatinib (D) with and without radiation. Cell viability was determined after 5-6 days treatment by CellTiter-Glo. **E,** Colony forming assay of CRC cell lines treated with trametinib, GDC-0994 or BI-3406 -/+ radiation. Scans of complete wells of standard six-well plates are shown (9.6 cm^2 per well). **F,** Colony forming assay of CRC cell lines treated with afatinib -/+ radiation. Scans of complete wells of standard six-well plates are shown (9.6 cm^2 per well).

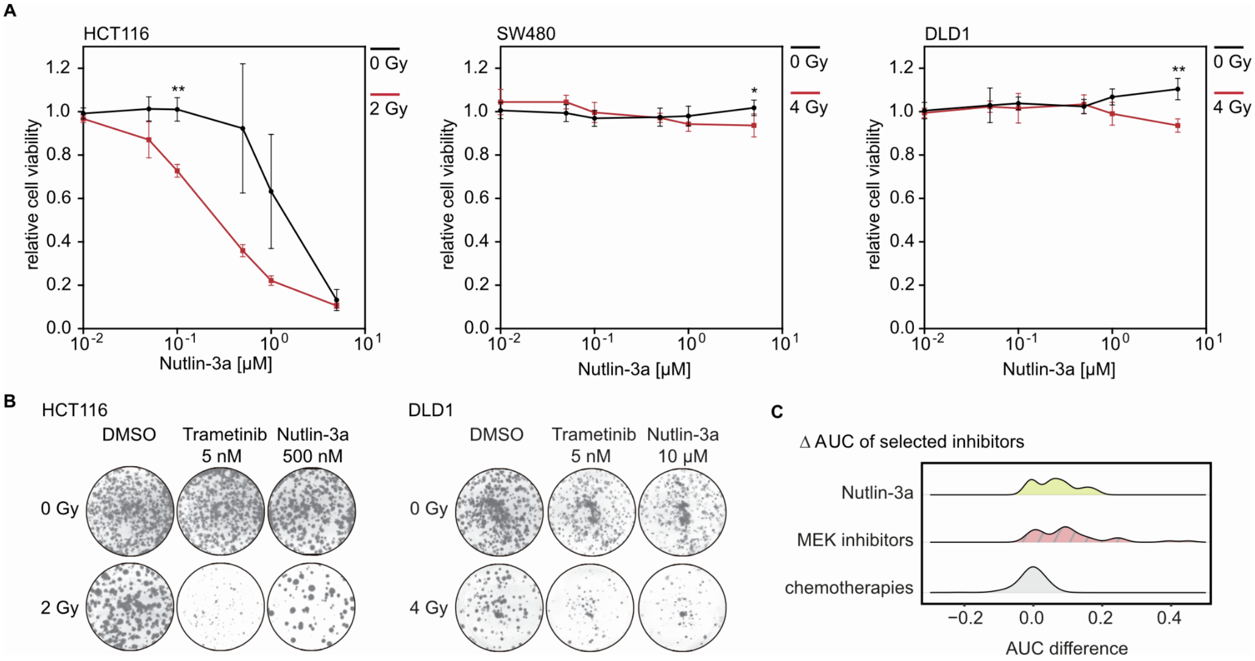

Figure S7: Effect of MDM2 inhibitor Nutlin-3a on radiosensitivity of CRC cell lines, related to Figure 2. A, Viability assay of CRC cell lines treated with increasing concentrations of MDM2 inhibitor Nutlin-3a with and without radiation. Cell viability was determined after 5-6 days of treatment by CellTiter-Glo. **B,** Colony forming assay of CRC cell lines treated with Nutlin-3a or trametinib -/+ radiation. Scans of complete wells of standard six-well plates are shown (9.6 cm² per well) **C,** Comparison of distribution of ΔAUC between Nutlin-3a, MEK inhibitors and chemotherapy drugs in organoid drug-radiation screen with the clinical library (Fig 2).

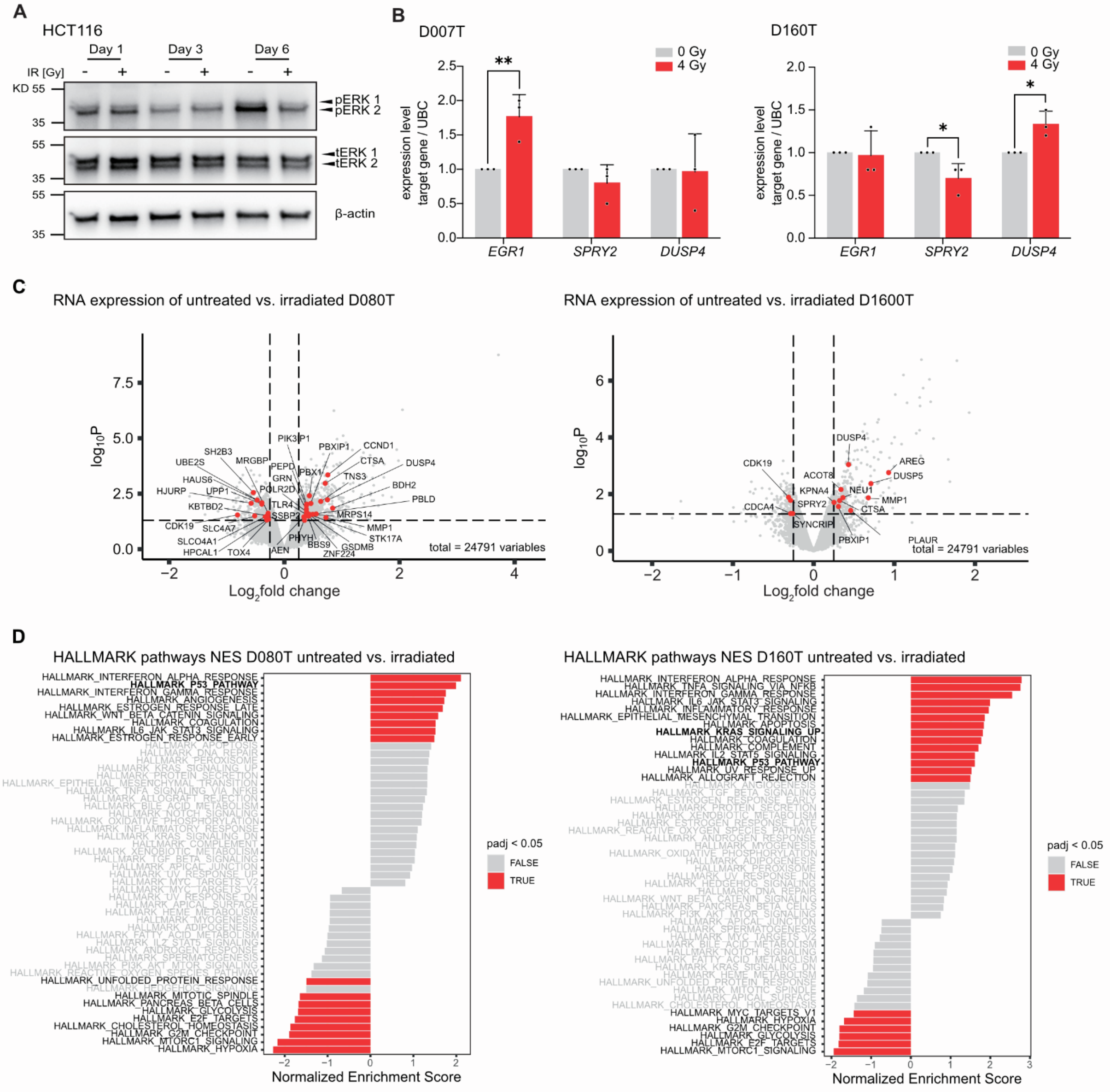
A
HCT116
Day 1
Day 3
Day 6
IR [Gy]
KD 55
35
pERK 1
pERK 2
tERK 1
tERK 2
β-actin
B
D007T
D160T
0 Gy
4 Gy
expression level target gene / UBC
EGR1
SPRY2
DUSP4
C
RNA expression of untreated vs. irradiated D080T
RNA expression of untreated vs. irradiated D1600T
log10P
Log2fold change
total = 24791 variables
D
HALLMARK pathways NES D080T untreated vs. irradiated
HALLMARK pathways NES D160T untreated vs. irradiated
padj < 0.05
FALSE
TRUE
Normalized Enrichment Score
HALLMARK_INTERFERON_ALPHA_RESPONSE
HALLMARK_P53_PATHWAY
HALLMARK_INTERFERON_GAMMA_RESPONSE
HALLMARK_ANGIOGENESIS
HALLMARK_ESTROGEN_RESPONSE_LATE
HALLMARK_WNT_BETA_CATENIN_SIGNALING
HALLMARK_COAGULATION
HALLMARK_IL6_JAK_STAT3_SIGNALING
HALLMARK_ESTROGEN_RESPONSE_EARLY
HALLMARK_UNFOLDED_PROTEIN_RESPONSE
HALLMARK_MITOTIC_SPINDLE
HALLMARK_PANCREAS_BETA_CELLS
HALLMARK_GLYCOLYSIS
HALLMARK_E2F_TARGETS
HALLMARK_CHOLESTEROL_HOMEOSTASIS
HALLMARK_G2M_CHECKPOINT
HALLMARK_MTORC1_SIGNALING
HALLMARK_HYPOXIA
HALLMARK_TNFA_SIGNALING_VIA_NFKB
HALLMARK_IL6_JAK_STAT3_SIGNALING
HALLMARK_INFLAMMATORY_RESPONSE
HALLMARK_EPITHELIAL_MESENCHYMAL_TRANSITION
HALLMARK_APOPTOSIS
HALLMARK_KRAS_SIGNALING_UP
HALLMARK_COMPLEMENT
HALLMARK_IL2_STAT5_SIGNALING
HALLMARK_UV_RESPONSE_UP
HALLMARK_ALLOGRAFT_REJECTION
HALLMARK_MYC_TARGETS_V1

Figure S8: Radiation-induced effects on signaling pathways, related to Figure 3. A, Phosphorylation of ERK1/2 in CRC line HCT116 at different time points after irradiation [IR]. **B,** Target gene expression of RAS-MAPK pathway after radiation in CRC organoid lines D007T and D160T determined by quantitative PCR. Data from three independent experiments are presented as mean ± SD. $^{*}p < 0.05$, two-tailed Student's t-test. **C,** RNA expression profiling of rectal cancer organoid lines D080T and D160T 96 h after radiation treatment with 4 Gy. Volcano plot of differentially expressed genes in irradiated vs. non-irradiated organoids. Target genes of the EGFR signaling pathway according to PROGENY are highlighted. D, Gene set enrichment analysis of HALLMARK gene sets in irradiated vs. non-irradiated organoids D080T and D160T. C-D, data from five (D080T) and four (D160T) biological replicates are shown.

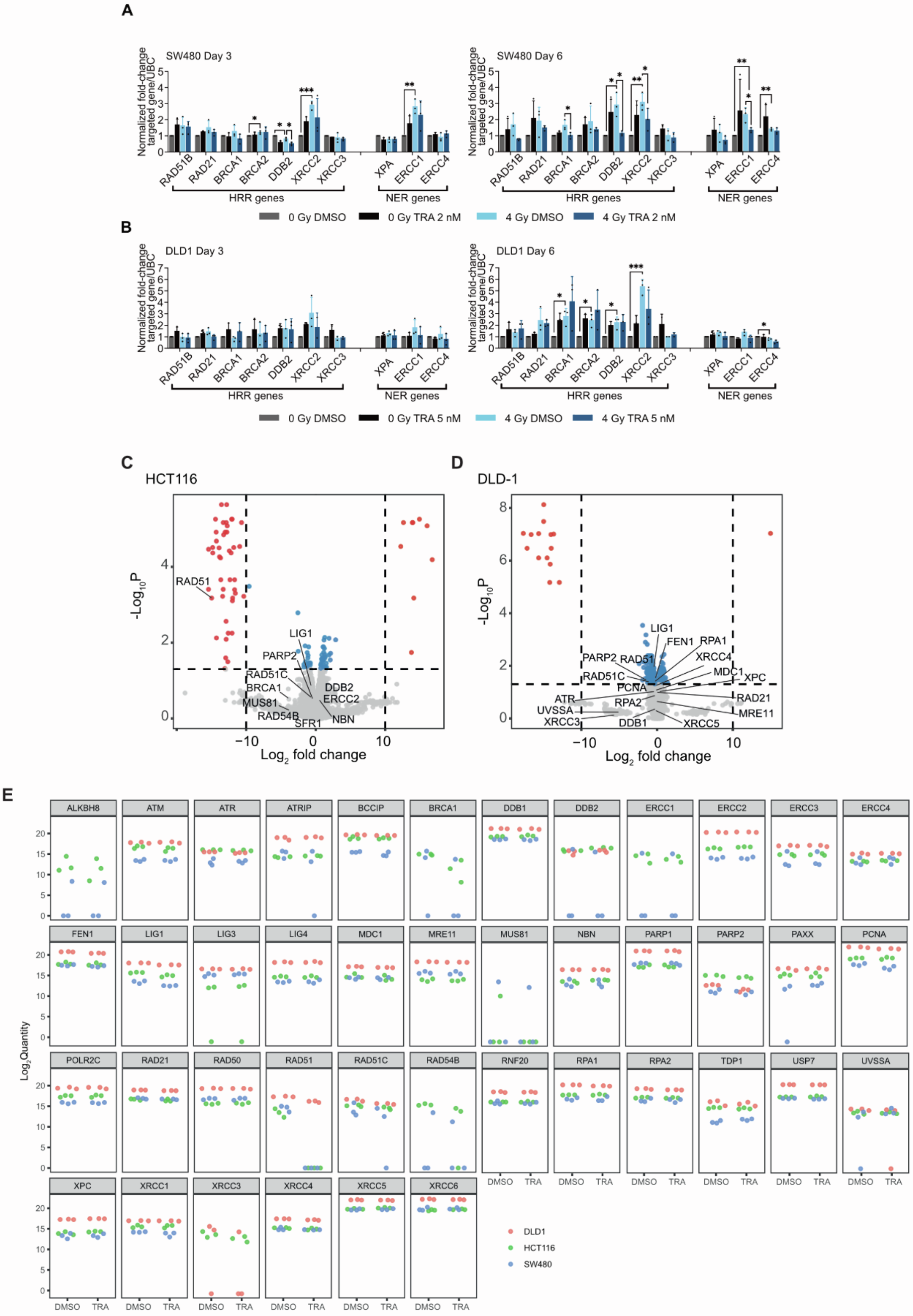
A
SW480 Day 3
SW480 Day 6
Normalized fold-change targeted gene/UBC
RAD51B
RAD21
BRCA1
BRCA2
DDB2
XRCC2
XRCC3
XPA
ERCC1
ERCC4
HRR genes
NER genes
0 Gy DMSO
0 Gy TRA 2 nM
4 Gy DMSO
4 Gy TRA 2 nM
B
DLD1 Day 3
DLD1 Day 6
0 Gy TRA 5 nM
4 Gy TRA 5 nM
C
HCT116
D
DLD-1
-Log10P
Log2 fold change
RAD51
LIG1
PARP2
RAD51C
BRCA1
MUS81
RAD54B
SFR1
NBN
DDB2
ERCC2
FEN1
RPA1
XRCC4
MDC1
XPC
RAD21
MRE11
XRCC5
DDB1
XRCC3
UVSSA
ATR
RPA2
PCNA
E
Log2Quantity
ALKBH8
ATM
ATRIP
BCCIP
ERCC3
FEN1
LIG3
LIG4
PARP1
PAXX
POLR2C
RAD50
RNF20
TDP1
USP7
XRCC1
XRCC6
DMSO
TRA
DLD1
HCT116
SW480

Figure S9: Proteomics analysis of radiation-induced effects on DNA repair pathways in CRC, related to Figure 4. A-B, Radiation-induced transcriptional changes of homologous recombination repair (HRR) genes and nucleotide excision repair (NER) genes in CRC cell lines. Expression of genes is determined by qPCR. Data from three independent experiments are presented as mean ± SD. $^*p < 0.05$, $^{**}p < 0.01$, $^{***}p < 0.001$, two-tailed Student's t-test. **C,** Global proteome profiling by mass spectrometry of HCT116 cells after treatment with 100 nM trametinib vs. DMSO for 24 h. **D,** Global proteome profiling by mass spectrometry of DLD1 cells after treatment with 100 nM trametinib vs. DMSO for 24 h. **E,** Abundance of selected DNA damage response pathway proteins in the three tested cell lines in the proteomics profiling experiments. **C-D,** three biological replicates were analyzed for each cell line.

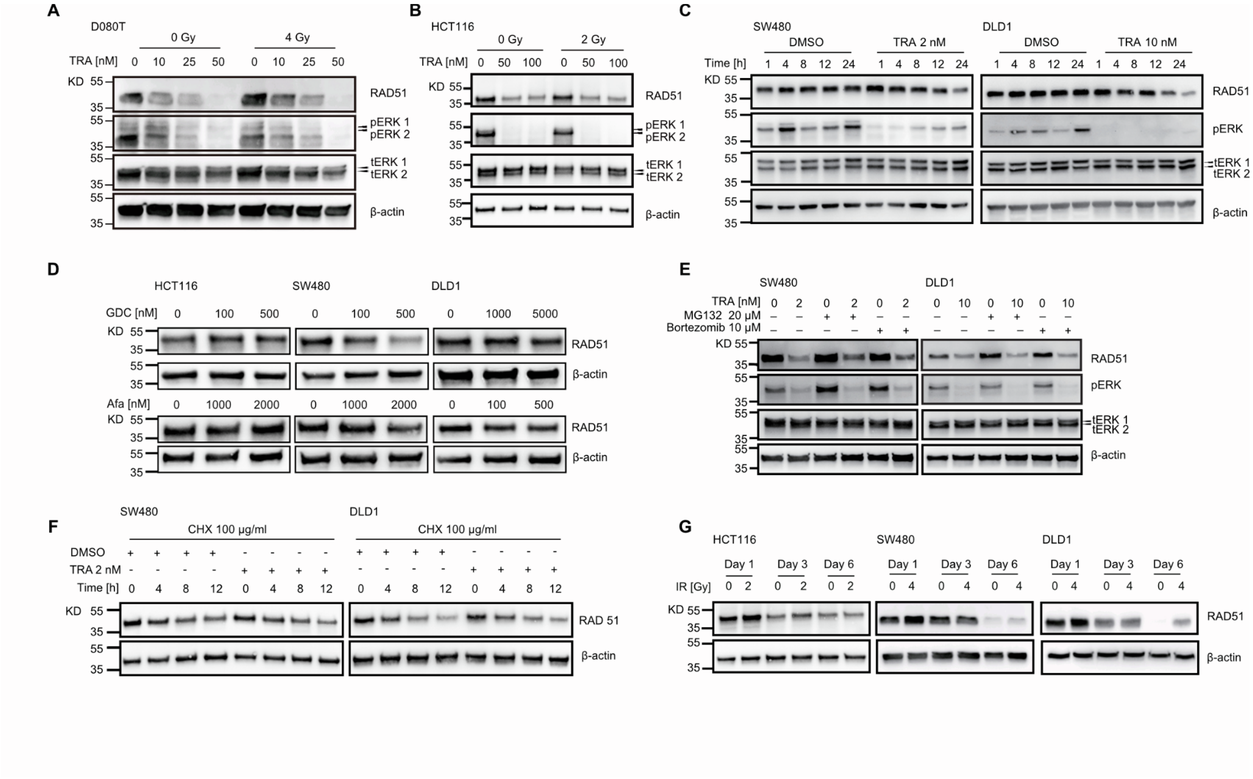

Figure S10: Radiation and MEK inhibition-induced effects on DNA repair pathway in CRC, related to Figure 4. A, Dose-dependent loss of RAD51 upon MEK inhibition with trametinib (TRA) after 48 h in the organoid line D080T. B, Dose-dependent loss of RAD51 upon MEK inhibition +/- radiation after 24 h in the CRC cell line HCT116. **C,** Temporal kinetics of RAD51 loss in SW480 and DLD1 cell lines after MEK inhibition. **D,** Differential effect of RAS-MAPK inhibitors (ERK1/2 inhibitor GDC-0994 [GDC] and EGFR inhibitor Afatinib [Afa]) on RAD51 levels in HCT116, SW480 and DLD1 cells. Cells were treated for 24 hours with the inhibitors. **E, I**nhibition of the proteasomal inhibitors bortezomib and MG132 does not prevent RAD51 loss upon MEK inhibition in the CRC cell lines SW480 and DLD1. CRC cell lines were pre-cultured for 24 h, and treated with trametinib or DMSO for 20 h, and then co-treated with proteasomal inhibitor (MG132 or bortezomib) or DMSO for 4 h. **F,** Cycloheximide chase assays with CRC cell lines. CRC cell lines were pre-incubated with trametinib for 8 h, following treatment with or without cycloheximide (CHX). No enhanced protein loss is observed upon treatment with the inhibitors. **G,** Radiation (IR) increases RAD51 protein levels in CRC cell lines at different time points. A-G, Representative images of three independent biological replicates are shown.

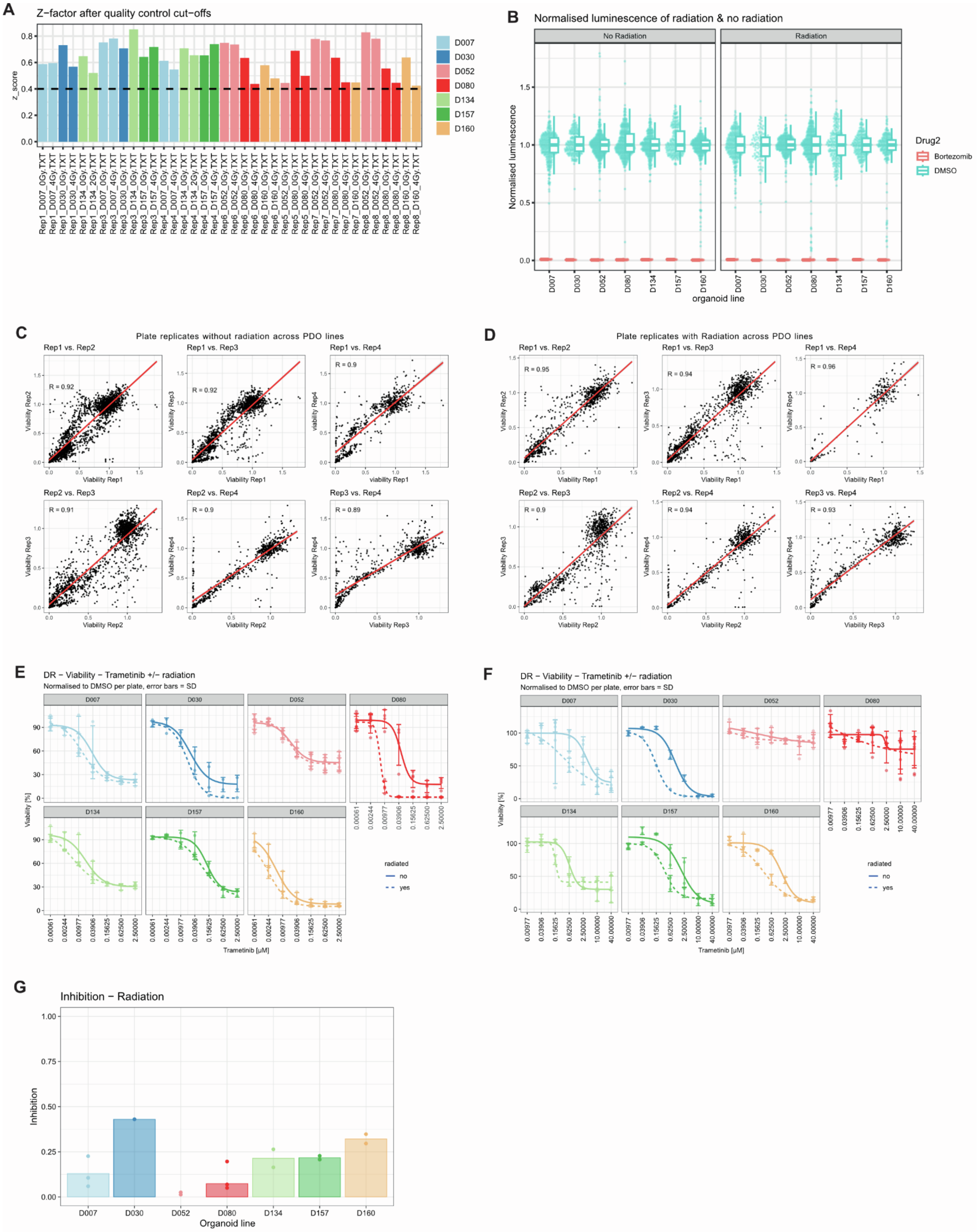
A
Z−factor after quality control cut−offs
z_score
D007
D030
D052
D080
D134
D157
D160
B
Normalised luminescence of radiation & no radiation
No Radiation
Radiation
Normalised luminescence
organoid line
Drug2
Bortezomib
DMSO
C
Plate replicates without radiation across PDO lines
Rep1 vs. Rep2
Rep1 vs. Rep3
Rep1 vs. Rep4
Rep2 vs. Rep3
Rep2 vs. Rep4
Rep3 vs. Rep4
R = 0.92
R = 0.92
R = 0.9
R = 0.91
R = 0.9
R = 0.89
D
Plate replicates with Radiation across PDO lines
Rep1 vs. Rep2
Rep1 vs. Rep3
Rep1 vs. Rep4
Rep2 vs. Rep3
Rep2 vs. Rep4
Rep3 vs. Rep4
R = 0.95
R = 0.94
R = 0.96
R = 0.9
R = 0.94
R = 0.93
E
DR − Viability − Trametinib +/− radiation
Normalised to DMSO per plate, error bars = SD
Viability [%]
Trametinib [µM]
radiated
no
yes
F
DR − Viability − Trametinib +/− radiation
Normalised to DMSO per plate, error bars = SD
Viability [%]
Trametinib [µM]
radiated
no
yes
G
Inhibition − Radiation
Inhibition
Organoid line
D007
D030
D052
D080
D134
D157
D160

Figure S11: Quality controls of drug-drug combination and drug-drug-radiation combination tests, related to Figures 5-6. A-D, Quality controls of drug-drug-radiation profiling experiments. **A,** z-score and of all tested plates after quality control cut-offs were applied: four plates (D030T Rep. 3, 4 Gy, D134T Rep. 3, 4 Gy, D052T Rep. 1, 4 Gy and D160T Rep. 2, 4 Gy) were excluded from further analysis. **B,** Normalized luminescence values of positive (bortezomib) and negative (DMSO) controls in radiation and non-radiation assays. **C-D,** Plate replicate correlations of drug-drug combination tests (C) and drug-drug-radiation tests (D). **E-G,** Single(/dual) response of tested organoid lines in drug-drug and drug-drug-radiation experiments. E, Response of all tested organoid lines to trametinib alone (with/without irradiation). **F,** Response of all tested organoid lines to talazoparib alone (with/without irradiation). **G,** Growth inhibition of all tested organoids after irradiation treatment alone. 2 biological replicates were analyzed for D030T, D157T, 3 replicates were analyzed for D007T, D052T, D134T and D160, and 4 replicates were analyzed for D080T.

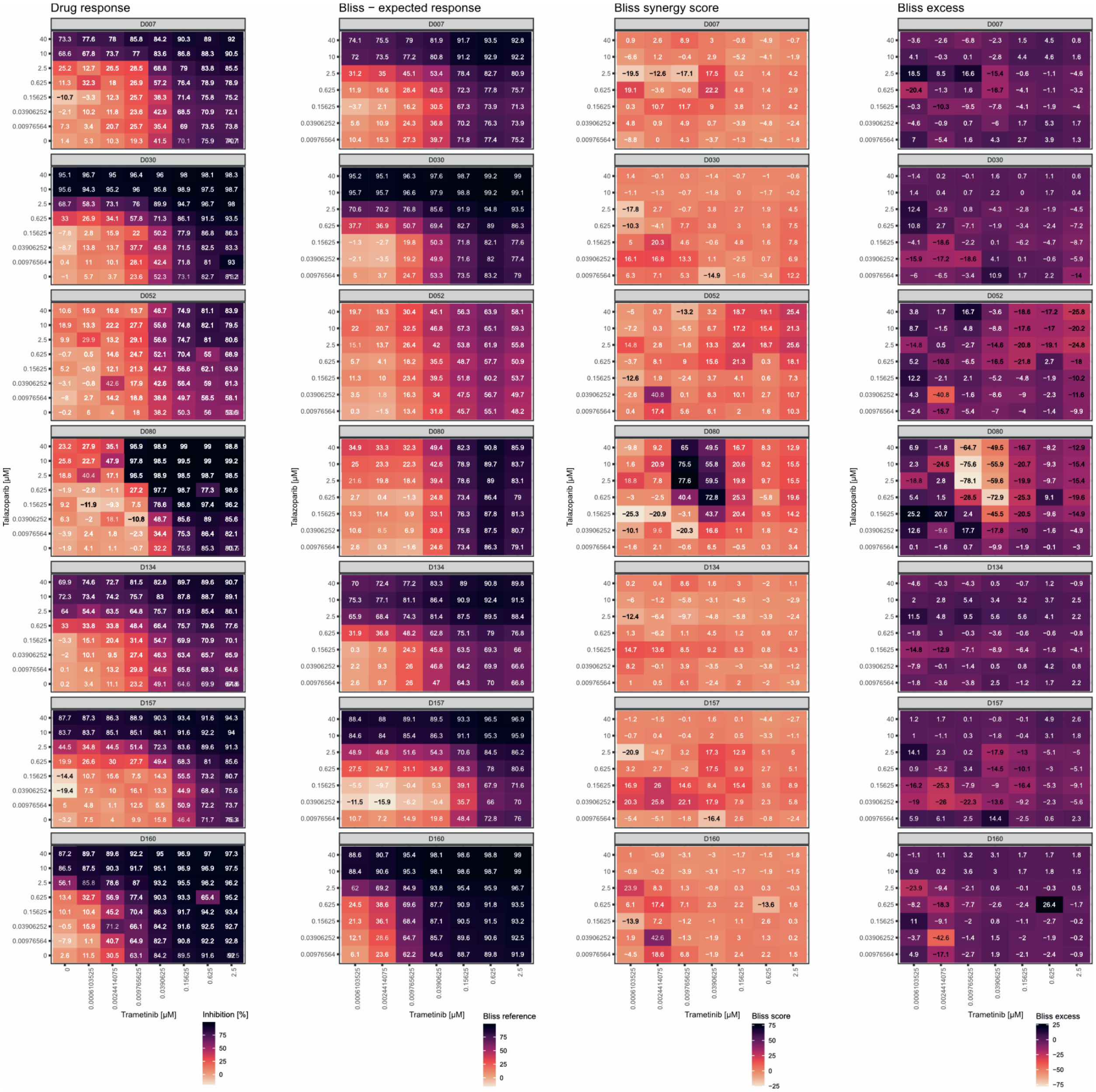
Drug response
Bliss − expected response
Bliss synergy score
Bliss excess
D007
D030
D052
D080
D134
D157
D160
Talazoparib [µM]
Trametinib [µM]
Inhibition [%]
Bliss reference
Bliss score
Bliss excess

Figure S12: PARP inhibitors synergize with MEK inhibitors in CRC models, related to Figure 5. Drug response, Bliss expected response, Bliss synergy score and Bliss excess calculated for all dose combinations of talazoparib and trametinib in all seven tested organoid lines. All data shown in this figure were obtained in absence of radiation treatment. 2 biological replicates were analyzed for D030T, D157T, 3 replicates were analyzed for D007T, D052T, D134T and D160, and 4 replicates were analyzed for D080T. Of note, due to a pipetting error in one of the master plates, six individual wells had to be excluded from analysis of organoid lines D052T, D080T and D160T, including 3 wells containing DMSO controls and 3 wells containing drug combinations. Missing values in matrices were imputed by bootstrapping within the synergyfinder package.

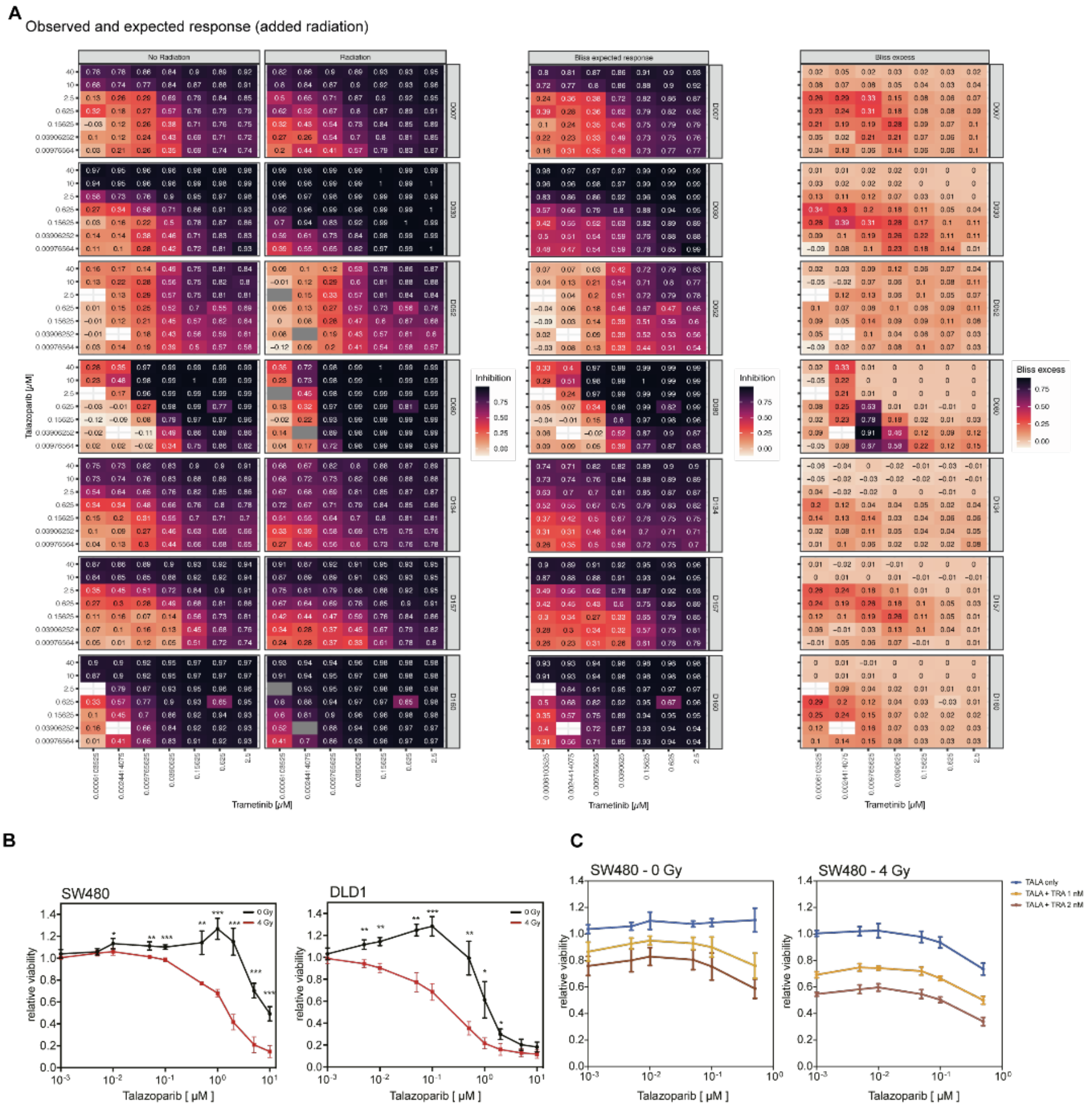
A
Observed and expected response (added radiation)
No Radiation
Radiation
Bliss expected response
Bliss excess
Inhibition
Bliss excess
Talazoparib [μM]
Trametinib [μM]
B
SW480
DLD1
0 Gy
4 Gy
relative viability
Talazoparib [μM]
C
SW480 - 0 Gy
SW480 - 4 Gy
TALA only
TALA + TRA 1 nM
TALA + TRA 2 nM
relative viability
Talazoparib [μM]

Figure S13: PARP inhibitors synergize with MEK inhibitors to enhance radiation response, related to Figure 6. A, Response/inhibition matrix derived from talazoparib - trametinib combinations for all seven tested organoid lines: Non-irradiated, irradiated, Bliss expected response, according to a model of added radiation to fixed combinations of trametinib and talazoparib, as well as Bliss excess (observed response - expected response) are shown. Data were normalized to non-irradiated DMSO controls. The complete matrices tested are shown for each drug. 2 biological replicates were analyzed for D030T, D157T, 3 replicates were analyzed for D007T, D052T, D134T and D160, and 4 replicates were analyzed for D080T. Of note, due to a pipetting error in one of the master plates, six individual wells had to be excluded from analysis of organoid lines D052T, D080T and D160T, including 3 wells containing DMSO controls and 3 wells containing drug combinations. **B,** Viability assays of CRC cell lines treated with increasing concentrations of PARP inhibitor talazoparib with and without radiation. Cell viability was determined after 60 hrs of treatment by CellTiter-Glo. **C,** Viability assays of CRC cell line SW480 treated with increasing concentrations of PARP inhibitor talazoparib in combination with selected low-dose trametinib treatments, with and without radiation. Cell viability was determined after 60 h of treatment by CellTiter-Glo. **B-C,** Data from three independent experiments are presented as mean ± SD. $^{*}p < 0.05$, $^{**}p < 0.01$, $^{***}p < 0.001$, two-tailed t-test.

Supplementary Tables

Table S1: Clinical characteristics of patient donors of colorectal cancer organoids, related to STAR Methods

Organoid	Sex	Location	Biopsy	T	N	M	Stage (UICC)	Grading (WHO)	Neoadjuvant Treatment	Dworak	mrTRG	Δ tumor length
D004T	f	rectum	primary	3	2	0	3	2	50,4 Gy + capecitabine	1	3	-2
D007T	m	rectum	primary	3	3	0	3	2	50,4 Gy + capecitabine	2	3	6
D027T	m	rectum	primary	4	1	1	4	2	N/A	N/A	N/A	N/A
D030T	f	colon	primary	3	0	0	2	2	N/A	N/A	N/A	N/A
D046T	f	rectum	primary	2	1	0	3	2	50,4 Gy + capecitabine	4	1	-6
D052T	m	rectum	primary	3	0	0	2	2	50,4 Gy + capecitabine	2	5	0
D073T	m	rectum	primary	3	+	0	3	N/A	50,4 Gy + FOLFOX	1	3	N/A
D080T	f	rectum	primary	3	3	0	3	1	50,4 Gy + capecitabine	1	5	1
D082T	f	rectum	primary	4	3	0	3	2	50,4 Gy + capecitabine	1	4	-1
D086T	f	rectum	primary	3	3	0	3	2	50,4 Gy + capecitabine	1	3	-1
D104T	f	rectum	primary	3	4	1	4	2	5 x 5 Gy	2	N/A	N/A
D114T	f	rectum	primary	3	3	0	3	2	50,4 Gy + capecitabine	4	2	4
D134T	f	rectum	primary	3	2	0	2	2	50,4 Gy + capecitabine	4	2	-2
D147T	m	rectum	metastasis	3	4	1	4	2	50,4 Gy + capecitabine	1	4	-2
D157T	f	colon	primary	3	4	1	4	2	N/A	N/A	N/A	N/A
D160T	m	rectum	primary	3	3	0	3	2	N/A	N/A	N/A	N/A

Abbr: T, Tumor; N, node; M, Metastasis; N/A, not available; Gy, Gray; +, positive; Dworak, Dworak pathological regression grade; mrTRG, MRI tumor regression grade.

Table S3: Library design of the clinical library, related to STAR Methods

Drug name	Category	Target	Cmax [µM]	Cmin [µM]	Cat#
5-FU	Chemotherapy	DNA-antimetabolite	100	0,160	S1209
Abemaciclib	Targeted	CDK4/6	10	0,016	S5716
Acalabrutinib	Targeted	BTK	50	0,080	S8116
Adagrasib	Targeted	KRAS G12C	50	0,080	S8884
Adavosertib	Targeted	Wee1	125	0,200	S1525
Afatinib	Targeted	EGFR, HER2, ErbB3, ERbB4	100	0,160	S1011
Alectinib	Targeted	ALK, RET	5	0,008	S2762
Alpelisib	Targeted	PI3Ka	125	0,200	S2814
Altretamine	Chemotherapy	DNA-crosslinks	50	0,080	S1278
Avapritinib	Targeted	PDGFRa D842V-mutation, c-Kit	50	0,080	S8553
Axitinib	Targeted	VEGFR1, VEGFR2, VEGFR3, PDGFR3, c-Kit	75	0,120	S1005
AZD 4547	Targeted	FGFR, VEGFR2 (KDR), IGFR, CDK2, p38	125	0,200	S2801
Belinostat	Targeted	HDAC	50	0,080	S1085
Binimetinib	Targeted	MEK1/2	25	0,040	S7007
Birinapant	Targeted	XIAP, cIAP1	25	0,040	S7015
Bleomycin	Chemotherapy	DNA-polymerase	50	0,080	S1214
Bortezomib	Targeted	proteasome	0,5	0,001	S1013
Bosutinib	Targeted	Abl/Src, PI3K/AKT/mTor, MAP/ERK, JAK/STAT3	50	0,080	S1014
Brigatinib	Targeted	ALK/ROS1, IGF-1R, FLT3, EGFR	5	0,008	S8229
Cabazitaxel	Chemotherapy	microtubules	2,5	0,004	S3022
Cabozantinib	Targeted	VEGFR2, c-Met, RET, Kit, Fit-1/3/4, Tie2, AXL	100	0,160	S1119
Capivasertib	Targeted	AKT	125	0,200	S8019
Capmatinib	Targeted	c-MET, RONb, EGFR, HER3, Wnt/b-catenin	12,5	0,020	S2788
Carboplatin	Chemotherapy	DNA-crosslinks	5	0,008	S1215
Carfilzomib	Targeted	proteasome	50	0,080	S2853
Carmustine	Chemotherapy	DNA-alkylating	75	0,120	S3669
Ceritinib	Targeted	ALK, IGF-1R, InsR, STK22d, FLT3	10	0,016	S7083
Cisplatin	Chemotherapy	DNA-crosslinks	75	0,120	S1166
Cobimetinib	Targeted	MEK1	50	0,080	S8041
Copanlisib	Targeted	PI3Ka/d	5	0,008	S2802
Crizotinib	Targeted	ALK/ROS1, c-MET	62,5	0,100	S1068
Cyclophosphamide	Chemotherapy	DNA-alkylating	75	0,120	S1217
Dabrafenib	Targeted	BRAFV600E	50	0,080	S2807
Dacarbazine	Chemotherapy	DNA-alkylating	50	0,080	S1221
Dacomitinib	Targeted	EGFR, ErbB2, ERbB4	50	0,080	S2727
Dactinomycin	Chemotherapy	DNA-Synthesis, Transcription, Topoisomerase II	75	0,120	S8964
Dasatinib	Targeted	Abl, Src, c-Kit	100	0,160	S1021
Defactinib	Targeted	FAK	37,5	0,060	S7654
Docetaxel	Chemotherapy	microtubules	2,5	0,004	S1148
Doxorubicin	Chemotherapy	DNA-topoisomerase II	50	0,080	S1208
Duvelisib	Targeted	PI3Kd/g	100	0,160	S7028
Enasidenib	Targeted	IDH-2	50	0,080	S8205
Encorafenib	Targeted	BRAFV600E	50	0,080	S7108
Entrectinib	Targeted	TRKA/B/C, ROS1, ALK	50	0,080	S7998
Epirubicin	Chemotherapy	DNA-topoisomerase	75	0,120	S1223
Erdafitinib	Targeted	FGFR, RET, CSF-1R, PDGFR-a/b, FLT4, KIT, VEGFR-2	100	0,160	S8401
Erlotinib	Targeted	EGFR	100	0,160	S7786
Etoposide	Chemotherapy	DNA-topoisomerase II	100	0,160	S1225
Everolimus	Targeted	mTor/FKBP12	25	0,040	S1120
Fedratinib	Targeted	JAK2, FLT3, Ret	50	0,080	S2736
Floxuridine	Chemotherapy	DNA-antimetabolite	50	0,080	S1299
Gedatolisib	Targeted	PI3Ka/g, mTor	5	0,008	S2628
Gefitinib	Targeted	EGFR	100	0,160	S1025
Gemcitabine	Chemotherapy	DNA-antimetabolite	12,5	0,020	S1714
Gilteritinib	Targeted	FLT3/AXL	15	0,024	S7754

Glasdegib	Targeted	Smoothened	100	0,160	S7160
Homoharringtonine	Targeted	STAT3, translation	50	0,080	S9015
Hydroxyurea	Chemotherapy	DNA-synthesis	75	0,120	S1896
Ibrutinib	Targeted	BTK	50	0,080	S2680
Idelalisib	Targeted	p110d	100	0,160	S2226
Ifosfamide	Chemotherapy	DNA-alkylating	75	0,120	S1302
Imatinib	Targeted	Abl, c-KIT, PDGFR	100	0,160	S2475
Ivosidenib	Targeted	IDH-1	50	0,080	S8206
Ixabepilone	Chemotherapy	microtubules	2,5	0,004	S7930
Ixazomib	Targeted	proteasome	2,5	0,004	S2180
Lapatinib	Targeted	EGFR, ErbB2 (HER2)	100	0,160	S2111
Larotrectinib	Targeted	TRKA/B/C	50	0,080	S5860
Lenalidomide	Targeted	CRBN, TNF-a	100	0,160	S1029
Lenvatinib	Targeted	VEGFR, FGFR, PDGFR, RET, c-Kit	75	0,120	S1164
LGK974	Targeted	PORCN	100	0,160	S7143
Lomustine	Chemotherapy	DNA-alkylating	75	0,120	S1840
Lorlatinib	Targeted	ALK/ROS1	50	0,080	S7536
Lurbinectedin	Chemotherapy	DNA-binding	5	0,008	S9603
Mechlorethamine	Chemotherapy	DNA-alkylating	75	0,120	S4252
Melphalan	Chemotherapy	DNA-alkylating	30	0,048	S8266
Methotrexat	Chemotherapy	DNA-antimetabolite	50	0,080	S1210
Midostaurin	Targeted	PKCa/b/g, Syk, c-Kit, c-Fgr, c-Src, FLT3, PDFRb, VEGFR1/2	50	0,080	S8064
Mitomycin C	Chemotherapy	DNA-crosslinks	12,5	0,020	S8146
Mitoxantrone	Chemotherapy	DNA-crosslinks, topoisomerase II	10	0,016	S1889
MK-8776	Targeted	Chk1	37,5	0,060	S2735
Napabucasin	Targeted	STAT3	25	0,040	S7977
Navitoclax	Targeted	Bcl-2, Bcl-xL, Bcl-w	75	0,120	S1001
Neratinib	Targeted	EGFR/HER2	25	0,040	S2150
Nilotinib	Targeted	Bcr-Abl	50	0,080	S1033
Niraparib	Targeted	PARP1/2	100	0,160	S2741
Nutlin3a	Targeted	p53/MDM2	100	0,160	S8059
Octreotide	Targeted	Somatostatine analogon	20	0,032	P1017
Olaparib	Targeted	PARP1/2	100	0,160	S1060
Osimertinib	Targeted	EGFR	100	0,160	S7297
Oxaliplatin	Chemotherapy	DNA-crosslinks	75	0,120	S1224
Paclitaxel	Chemotherapy	microtubules	2,5	0,004	S1150
Palbociclib	Targeted	CDK4/6	25	0,040	S1116
Panobinostat	Targeted	HDAC	2,5	0,004	S1030
Pazopanib	Targeted	VEGFR1/2/3, PDGFR, FGFR, c-Kit, c-Fms	100	0,160	S3012
PD0166285	Targeted	Wee1, Chk1	25	0,040	S8148
Pemetrexed	Chemotherapy	DNA-antimetabolite	50	0,080	S1135
Pemigatinib	Targeted	FGFR1/2/3/4	50	0,080	S0088
Pexidartinib	Targeted	CSF-1R, Kit, FLT3	100	0,160	S7818
Pomalidomide	Targeted	CRBN, TNF-a	100	0,160	S1567
Ponatinib	Targeted	Abl, PDGFR, VEGFR2, FGFR1, Src	125	0,200	S1490
Poziotinib	Targeted	HER1,2,4	50	0,080	S7358
Pralsetinib	Targeted	c-RET	50	0,080	S8716
PRI-724	Targeted	b-catenin/CBP/p300	75	0,120	S8968
Regorafenib	Targeted	VEGFR1/2/3, PDGFR, Kit, Ret, Raf-1	100	0,160	S1178
Ribociclib	Targeted	CDK4/6	25	0,040	S7440
Ripretinib	Targeted	PDGFR-a, c-Kit	75	0,120	S8757
Romidepsin	Targeted	HDAC1/2	75	0,120	S3020
Rucaparib	Targeted	PARP	100	0,160	S1098
Ruxolitinib	Targeted	JAK1/2	100	0,160	S1378
Selinexor	Targeted	CRM1/Exportin1	100	0,160	S7252
Selpercatinib	Targeted	c-RET	75	0,120	S8781
Selumetinib	Targeted	MEK1/2	12,5	0,020	S1008
SN-38 (Irinotecan)	Chemotherapy	DNA-topoisomerase I	5	0,008	S4908
Sonidegib	Targeted	Smoothened	100	0,160	S2151
Sorafenib	Targeted	BRAF, RAF, VEGFR2/3, PDGFR-b, Fit-3, c-Kit	100	0,160	S7397

Streptozocin	Chemotherapy	DNA-alkylating	75	0,120	S1312
Sunitinib	Targeted	VEGFR2, PDGFR-b	50	0,080	S7781
Talazoparib	Targeted	PARP1/2	50	0,080	S7048
TAS-102	Chemotherapy	DNA-antimetabolite	100	0,160	S8539
Taselisib	Targeted	PI3Ka/d/g	25	0,040	S7103
Tazemetostat	Targeted	EZH2	75	0,120	S7128
Temozolomide	Chemotherapy	DNA-alkylating	75	0,120	S1237
Temsirolimus	Targeted	mTOR	75	0,120	S1044
Thiotepa	Chemotherapy	DNA-alkylating	75	0,120	S1775
Topotecan	Chemotherapy	DNA-topoisomerase I	5	0,008	S9321
Trametinib	Targeted	MEK1/2	2,5	0,004	S2673
Tucatinib	Targeted	HER2	100	0,160	S8362
Valrubicin	Chemotherapy	DNA-topoisomerase II	20	0,032	S9522
Vandetanib	Targeted	VEGFR2, VEGFR3, EGFR, RET	50	0,080	S1046
Vemurafenib	Targeted	BRAFV600E	100	0,160	S1267
Venetoclax	Targeted	Bcl-2	25	0,040	S8048
Vinblastin	Chemotherapy	microtubules	0,5	0,001	S4505
Vinorelbine	Chemotherapy	microtubules	2,5	0,004	S4269
Vismodegib	Targeted	Smoothened	100	0,160	S1082
Vistusertib	Targeted	mTOR	25	0,040	S2783
Volasertib	Targeted	PLK1	5	0,008	S2235
Vorinostat	Targeted	HDAC	100	0,160	S1047
XAV-939	Targeted	Wnt/b-catenin	100	0,160	S1180
YM155	Targeted	Survivin	5	0,008	S1130
Zanubrutinib	Targeted	BTK	50	0,080	S8791

Table S4: Primers for quantitative PCR, related to STAR Methods

Target gene	Species	Forward primer	Reverse primer
BRCA1	human	TTGTTGATGTGGAGGAGCAA	GATTCCAGGTAAGGGGTTCC
BRCA2	human	GAAAATCAAGAAAAATCCTTAAAG GCT	GTAATCGGCTCTAAAGAA ACATGATG
EGR1	human	AGCCCTACGAGCACCTGAC	GGTTTGGCTGGGGTAACTG
DDB2	human	CTCCTCAATGGAGGGAACAA	GTGACCACCATTCGGCTACT
DUSP4	human	GGCGGCTATGAGAGGTTTTCC	TGGTCGTGTAGTGGGGTCC
RAD21	human	AATTTGGCTAGCGGCCCAT	TGTCCGTAATGCCATTTTCACC
RAD51	human	GGTGAAGGAAAGGCCATGTA	GGGTCTGGTGGTCTGTGTT
RAD51B	human	GCACAAAGGTCTGCTGATTTC	CCCATGTTGGTGGGTAATGT
SPRY2	human	CCTACTGTCGTCCCAAGACCT	GGGGCTCGTGCAGAAGAAT
UBC	human	CTGATCAGCAGAGGTTGATCT TT	TCTGGATGTAGTCAGACAGG
XRCC2	human	TCACCTGTGCATGGTGATATT	TTCCAGGCCACCTTCTGATT
